# Effect of Once-Weekly Azithromycin vs Placebo in Children With HIV-Associated Chronic Lung Disease: The BREATHE Randomized Clinical Trial

**DOI:** 10.1001/jamanetworkopen.2020.28484

**Published:** 2020-12-17

**Authors:** Rashida A. Ferrand, Grace McHugh, Andrea M. Rehman, Hilda Mujuru, Victoria Simms, Edith D. Majonga, Mark P. Nicol, Trond Flaegstad, Tore J. Gutteberg, Carmen Gonzalez-Martinez, Elizabeth L. Corbett, Sarah L. Rowland-Jones, Katharina Kranzer, Helen A. Weiss, Jon O. Odland

**Affiliations:** 1 Department of Clinical Research, London School of Hygiene and Tropical Medicine, London, United Kingdom; 2 Biomedical Research and Training Institute, Harare, Zimbabwe; 3 MRC International Statistics and Epidemiology Group, Department of Infectious Disease Epidemiology, London School of Hygiene and Tropical Medicine, London, United Kingdom; 4 Department of Paediatrics, University of Zimbabwe, Harare, Zimbabwe; 5 Division of Clinical Microbiology, University of Cape Town, Cape Town, South Africa; 6 School of Biomedical Sciences, University of Western Australia, Perth, Australia; 7 Faculty of Health Sciences, UiT, The Arctic University of Norway, Tromsø, Norway; 8 Department of Paediatrics, University Hospital of North Norway, Tromsø, Norway; 9 Department of Microbiology and Infection Control, University Hospital of North Norway, Tromsø, Norway; 10 Malawi-Liverpool-Wellcome Trust Clinical Research Programme, Blantyre, Malawi; 11 Department of Paediatrics and Child Health, University of Malawi College of Medicine, Blantyre, Malawi; 12 Nuffield Department of Medicine, University of Oxford, Oxford, United Kingdom; 13 School of Health Systems and Public Health, Faculty of Health Sciences, University of Pretoria, Pretoria, South Africa

## Abstract

**Importance:**

HIV-associated chronic lung disease (HCLD) in children is associated with small airways disease, is common despite antiretroviral therapy (ART), and is associated with substantial morbidity. Azithromycin has antibiotic and immunomodulatory activity and may be effective in treating HCLD through reducing respiratory tract infections and inflammation.

**Objective:**

To determine whether prophylactic azithromycin is effective in preventing worsening of lung function and in reducing acute respiratory exacerbations (AREs) in children with HCLD taking ART.

**Design, Setting, and Participants:**

This double-blind, placebo-controlled, randomized clinical trial (BREATHE) was conducted between 2016 and 2019, including 12 months of follow-up, at outpatient HIV clinics in 2 public sector hospitals in Malawi and Zimbabwe. Participants were randomized 1:1 to intervention or placebo, and participants and study personnel were blinded to treatment allocation. Participants included children aged 6 to 19 years with perinatally acquired HIV and HCLD (defined as forced expiratory volume in 1 second [FEV_1_] *z* score < −1) who were taking ART for 6 months or longer. Data analysis was performed from September 2019 to April 2020.

**Intervention:**

Once-weekly oral azithromycin with weight-based dosing, for 48 weeks.

**Main Outcomes and Measures:**

All outcomes were prespecified. The primary outcome was the mean difference in FEV_1_ *z* score using intention-to-treat analysis for participants seen at end line. Secondary outcomes included AREs, all-cause hospitalizations, mortality, and weight-for-age *z* score.

**Results:**

A total of 347 individuals (median [interquartile range] age, 15.3 [12.7-17.7] years; 177 boys [51.0%]) were randomized, 174 to the azithromycin group and 173 to the placebo group; 162 participants in the azithromycin group and 146 placebo group participants had a primary outcome available and were analyzed. The mean difference in FEV_1_ *z* score was 0.06 (95% CI, −0.10 to 0.21; *P* = .48) higher in the azithromycin group than in the placebo group, a nonsignificant difference. The rate of AREs was 12.1 events per 100 person-years in the azithromycin group and 24.7 events per 100 person-years in the placebo groups (hazard ratio, 0.50; 95% CI, 0.27 to 0.93; *P* = .03). The hospitalization rate was 1.3 events per 100 person-years in the azithromycin group and 7.1 events per 100 person-years in the placebo groups, but the difference was not significant (hazard ratio, 0.24; 95% CI, 0.06 to 1.07; *P* = .06). Three deaths occurred, all in the placebo group. The mean weight-for-age *z* score was 0.03 (95% CI, −0.08 to 0.14; *P* = .56) higher in the azithromycin group than in the placebo group, although the difference was not significant. There were no drug-related severe adverse events.

**Conclusions and Relevance:**

In this randomized clinical trial specifically addressing childhood HCLD, once-weekly azithromycin did not improve lung function or growth but was associated with reduced AREs; the number of hospitalizations was also lower in the azithromycin group but the difference was not significant. Future research should identify patient groups who would benefit most from this intervention and optimum treatment length, to maximize benefits while reducing the risk of antimicrobial resistance.

**Trial Registration:**

ClinicalTrials.gov Identifier: NCT02426112

## Key Points

**Question:**

What is the effect of weekly azithromycin on morbidity in children with HIV-associated chronic lung disease?

**Findings:**

In this randomized clinical trial that included 347 children aged 6 to 19 years, azithromycin did not improve lung function. The rate of acute respiratory exacerbations was 12.1 events per 100 person-years in the azithromycin group and 24.7 events per 100 person-years in the control group; the hospitalization rate was 1.3 events per 100 person-years in the azithromycin group and 7.1 events per 100 person-years in the placebo group.

**Meaning:**

These findings suggest that prophylactic azithromycin has no effect on lung function in children with HIV-associated chronic lung disease but it is associated with a lower rate of acute respiratory exacerbations.

## Teaser

This randomized clinical trial examines whether prophylactic azithromycin is effective in preventing worsening of lung function and in reducing acute respiratory exacerbations in children with HIV-associated chronic lung disease taking antiretroviral therapy.

## Introduction

Antiretroviral therapy (ART) and cotrimoxazole prophylaxis have resulted in a dramatic reduction in mortality among children with HIV globally.^1^ However, studies in the ART era in sub-Saharan Africa, where 90% of children with HIV live, have demonstrated that approximately 30% of perinatally HIV-infected older children and adolescents have chronic lung disease.^2,3^

In the pre-ART era, the most common cause of HIV-associated chronic lung disease (HCLD) was lymphoid interstitial pneumonitis, a condition that responds well to ART and is now rarely seen in clinical practice.^4,5^ HCLD remains highly prevalent among children in sub-Saharan Africa despite ART but is a clinical entity distinct from that in the pre-ART era. The typical clinical picture is that of chronic cough, hypoxia, breathlessness, and substantially reduced exercise tolerance.^3,6,7^ Lung function is commonly impaired, with an obstructive irreversible pattern, and abnormalities are subtle on radiographs.^3,8,9^ The predominant findings on computed tomography are mosaic decreased attenuation^9,10,11^ consistent with constrictive obliterative bronchiolitis (OB).^12,13^

No association has been observed between abnormal lung function and ART use or duration, and HCLD is therefore likely unresponsive to ART once established.^14,15^ Despite being common, there is no evidence base to guide management of childhood HCLD, often resulting in presumptive treatment for tuberculosis.^16^

Azithromycin (AZM) has bacteriostatic activity against the most common respiratory bacterial pathogens, as well as a robust immunomodulatory effect. Specifically, AZM has direct activity on airway epithelial cells to maintain their function and reduce mucus secretion.^17^ These characteristics have resulted in AZM being used to treat a variety of chronic lung diseases.^18,19,20^ OB most commonly occurs as a consequence of respiratory tract infections, with development closely associated with severe viral infections in the early years of life.^12,21^ HIV is associated with both a high incidence of respiratory infections and chronic systemic immune activation, despite ART.^22,23,24^ OB in the context of HIV infection results from inflammation either due to HIV or as a sequelae of respiratory infections, which consequently leads to aberrant fibroproliferative remodeling and fibrosis of the small airways.^12^ This provides the rationale for testing the efficacy of AZM in patients with HCLD. We conducted a randomized clinical trial to test the hypothesis that AZM is effective in preventing worsening of lung function and in reducing acute respiratory exacerbations (AREs) in children and adolescents with HCLD who are receiving ART.

## Methods

### Study Design

The Bronchopulmonary Function in Response to Azithromycin Treatment for Chronic Lung Disease in HIV-Infected Children (BREATHE) trial was a double-blind, placebo-controlled, randomized clinical trial conducted in Malawi and Zimbabwe. The trial protocol is available in Supplement 1, and baseline characteristics of the participants have been published elsewhere.^25,26^

Ethical approval was granted by the Malawi College of Medicine research ethics committee, the Medical Research Council of Zimbabwe, the Biomedical Research and Training Institute institutional review board in Zimbabwe, the London School of Hygiene and Tropical Medicine ethics committee, the University of Cape Town research ethics committee, and the Regional Ethics Committee for Medical and Health Research in Norway. Written informed consent was sought from the guardian, and age-appropriate assent was sought from the participant (for those aged <18 years); those aged 18 years and older consented independently. This study follows the Consolidated Standards of Reporting Trials (CONSORT) reporting guideline.

### Participants

Individuals aged 6 to 19 years attending outpatient HIV clinics at 2 public sector hospitals in Harare, Zimbabwe, and Blantyre, Malawi, were eligible for enrollment if mother-to-child HIV transmission was the most likely source of infection and if the following criteria were met: (1) patients had been taking first-line or second-line ART for at least 6 months; (2) patients had HCLD, defined as forced expiratory volume in 1 second (FEV_1_) *z* score less than −1.0 (after a protocol change in January 2017, to increase generalizability, from *z* score < −1.64 in the original protocol; individuals with an FEV_1_ *z* score between −1.0 and −1.64 before the protocol change were invited for rescreening) and lack of reversibility (<12% improvement in FEV_1_) with salbutamol (200 μg) inhaled using a spacer; (3) patients had a stable home address; (4) patients had been disclosed their HIV status (for those aged ≥12 years); and (5) patients had a guardian able to provide consent (for those aged <18 years). Exclusion criteria were having a condition that could be fatal during the study period, acute respiratory tract infection or tuberculosis at screening, pregnancy or breastfeeding, history of cardiac arrhythmia, a prolonged corrected QT (QTc) interval (>440 ms in male patients and >460 ms in female patients), creatinine clearance less than 30 ms/minute, alanine aminotransferase more than 2 times the upper limit of normal, known macrolide hypersensitivity, and use of drugs known to prolong the QTc interval. Tuberculosis screening was performed using the Xpert MTB/RIF assay (Cepheid) on 1 sputum sample obtained either spontaneously or through induction.

### Trial Procedures

Participants were randomly assigned in a 1:1 allocation ratio to receive either an oral weekly dose of AZM or placebo tablets of identical appearance for 48 weeks. Dosing was by participant weight: 10 to 19.9 kg, 250 mg; 20 to 29.9 kg, 500 mg; 30 to 39.9 kg, 750 mg; and 40 kg or more, 1250 mg. The randomization schedule and allocation list were generated by an independent statistician. Randomization was performed with block sizes ranging from 2 to 6 participants and was stratified by country. Participants and study personnel, including laboratory personnel and staff conducting outcome assessments, were blinded to treatment allocation.

After enrollment, participants were followed up at 2 weeks and every 12 weeks thereafter. At each visit, participants were asked about symptoms, adverse effects, use of antibiotics or other drugs, and adherence. The required number of tablets, with a buffer of 2 weeks, was dispensed by pharmacists at baseline and at the 2-, 12-, 24- and 36-week visits. We defined an adherent participant as not missing, on average, more than 2 of the 12 (13 in the first period) dispensed doses, as assessed by pill count, splitting time in the study into 4 12-week periods, as per visit and study medication dispensing schedule.

Participants were instructed to attend the clinic if they developed acute symptoms, which were graded using Division of AIDS (DAIDS) criteria. For diarrheal episodes of DAIDS severity grade 3, a rectal swab and *Clostridium difficile* rapid test was taken (*C. Diff* Quick Check Complete; Alere), and if the results were positive, the patient was treated with metronidazole for 7 days. AREs were defined as new or worsening respiratory symptoms with or without symptoms and signs of an infection as assessed by a clinician. Participants were specifically counseled to present to the study clinic if they developed respiratory symptoms for assessment and were encouraged to do so at every study visit.

Participants with a suspected ARE had sputum and nasal swabs taken and were treated with amoxicillin-clavulanate for 10 days; if there was no improvement, a chest radiograph was obtained and sputum culture for tuberculosis was performed. For febrile episodes, blood cultures and malaria testing (using microscopy or rapid diagnostic tests) were performed with management according to national guidelines. For other acute symptoms, management was at the discretion of the treating clinician.

The primary outcome was assessed at 48 weeks (window period, 44-52 weeks). The initial trial protocol stipulated follow-up to and measurement of FEV_1_ *z* score at 72 weeks as a secondary end point to examine the durability of the intervention’s effect. However, because of slow recruitment, the enrollment period was increased, and follow-up of all participants beyond 48 weeks was therefore not feasible.

### Outcomes

All outcomes were prespecified. The primary outcome was FEV_1_ *z* score at 48 weeks, calculated using the African American module of the Global Lung Function Initiative 2012 reference equations.^27^ The secondary outcomes were time to first ARE and number of AREs, death, all-cause hospitalization, infectious episodes (*Salmonella typhi*, gastroenteritis, or malaria), and weight-for-age *z* score (calculated using the British 1990 reference equations).^28^ All hospitalizations (defined as a period of stay in a hospital >24 hours) were recorded whether or not participants visited the study clinic. These were obtained through a combination of self-report and confirmation from patient-held records.

### Statistical Analysis

The study was designed to detect a standardized mean difference in FEV_1_ *z* score between trial groups of 0.32 SD with 80% statistical power and a significance level of *P* < .05, assuming a mean (SD) in the control group of −2.04 (0.82) and a difference in means of 0.26. Available data from 300 participant assessments at 48 weeks were required for this.

Analyses were performed using Stata statistical software version 16.0 (StataCorp) following a prespecified analysis plan (Supplement 1). All randomized participants were included, using intention-to-treat principles. All analyses were adjusted for trial site and for baseline factors agreed to be unbalanced between groups by trial investigators before data analysis. Linear regression was used to analyze quantitative outcomes as adjusted mean differences (AMDs), additionally adjusted for their comparable value at baseline. The normality of residuals was assessed with quantile-quantile and kernel density plots. Homoscedasticity was assessed visually using the Cook-Weisburg test, and robust SEs were used when there was evidence of heteroscedasticity.

Per-protocol analyses were conducted for participants who adhered to their randomized treatment. Prespecified subgroup and sensitivity analyses (exclusion of participants who were found after randomization but before data analysis, on recalculating enrollment FEV *z* score to account for anomalies in recorded height, to not meet the FEV_1_ inclusion criteria) and post hoc sensitivity analysis (multiple imputation for FEV_1_ *z* score for those with missing outcome data except deaths; see the eAppendix in Supplement 2) were performed for the primary outcome.

Event outcomes were analyzed using time-to-event methods. Participants were censored at date of death, date of withdrawal, date of last study visit (if lost to follow-up), or at 49 weeks after commencing study medication. Cumulative incidence curves were generated, and Cox regression and 2-sided Wald tests were used to compare trial groups, with robust SEs to account for multiple event data. The proportional hazards assumption was assessed using Schoenfeld residuals. Data analysis was performed from September 2019 to April 2020.

## Results

### Characteristics of Participants

**Figure 1.**
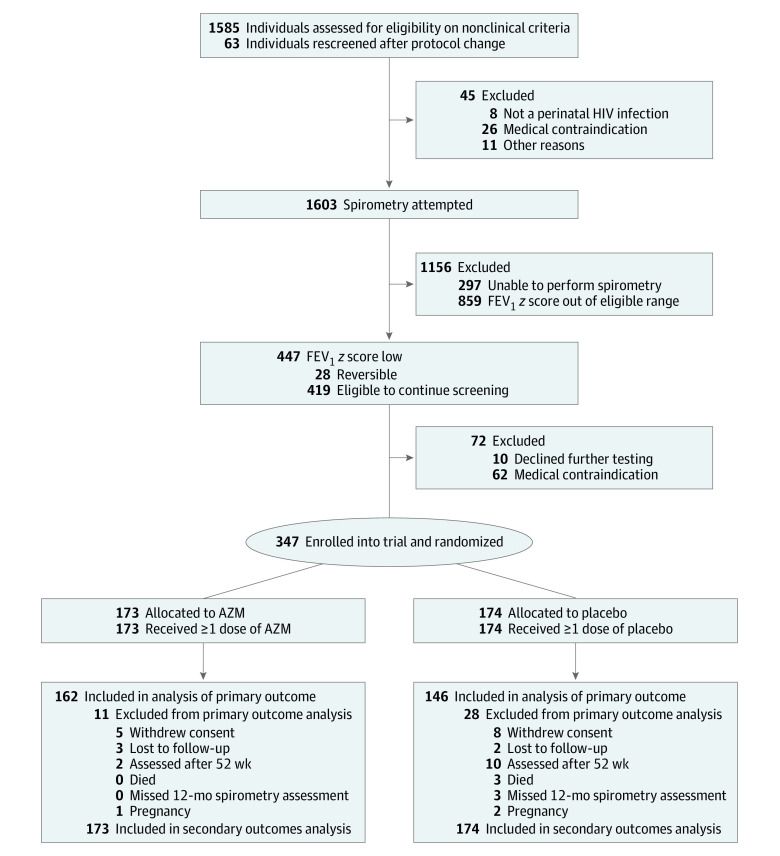
Participant Enrollment Flowchart AZM indicates azithromycin; and FEV_1_, forced expiratory volume in 1 second.

Between June 15, 2016, and September 4, 2018, 1585 individuals were assessed for eligibility, with 419 (26.4%) meeting lung function eligibility criteria. A total of 347 participants were enrolled and randomly assigned (173 to receive AZM and 174 to receive placebo) (Figure 1). We included in the analysis 11 participants (7 in the AZM group and 4 in the placebo group) found after randomization to not meet the FEV_1_ inclusion criteria.

Participants had a median (interquartile range [IQR]) age of 15.3 (12.7-17.7) years, and 177 (51.0%) were boys. The mean (SD) FEV_1_ *z* score at baseline was −2.00 (0.75) and was comparable between trial groups (−2.01 [0.76] in the AZM group vs −2.00 [0.74] in the placebo group). By chance, we observed differences between trial groups in the distributions of age, sex, and HIV viral load, and so all analyses estimating intervention effects were adjusted for these factors, with age-defined cutoffs based on O’Leary et al^29^ (Table 1).

**Table 1.** Characteristics of the Participants at Baseline

Characteristics	Participants, No. (%)	
	AZM group (n = 173)	Placebo group (n = 174)
Demographic characteristics		
Age, median (IQR), y	14.7 (12.6-16.8)	15.8 (13.0-18.1)
Female	80 (46.2)	90 (51.7)
Currently in school^a^	146 (84.4)	139 (79.9)
HIV characteristics		
Age at diagnosis, median (IQR), y	7.2 (3.5-9.9)	8.3 (5.2-11.1)
Cotrimoxazole prophylaxis	157 (90.7)	156 (89.7)
Duration taking antiretroviral therapy, median (IQR), y	5.9 (3.8-9.0)	6.4 (3.9-8.2)
HIV viral load log_10_ copies/mL, median (IQR)^a^	2.5 (1.6-4.0)	2.7 (1.7-4.1)
HIV viral load <1000 copies/mL^a^	100 (58.5)	94 (54.0)
CD4 cell count/mm^3^, median (IQR)	601 (417-784)	550 (325-779)
Lung function characteristics, mean (SD)		
FEV_1_ *z* score	−2.01 (0.76)	−2.00 (0.74)
FEV_1_, L	1.59 (0.50)	1.71 (0.53)
FEV_1_, %	73.3 (10.3)	73.6 (10.2)
FVC *z* score^a^	−1.77 (0.97)	−1.71 (0.89)
FVC, L	1.89 (0.59)	2.04 (0.63)
FVC, %^a^	77.8 (12.0)	78.4 (11.0)
FEV_1_:FVC ratio *z* score^a^	−0.66 (1.14)	−0.74 (1.13)
FEV_1_:FVC ratio^a^	0.85 (0.08)	0.84 (0.08)
Clinical characteristics		
Weight-for-age *z* score, mean (SD)	−2.23 (1.43)	−2.07 (1.50)
Underweight^b^	98 (56.7)	83 (47.7)
Height-for-age *z* score, mean (SD)	−2.16 (1.18)	−2.04 (1.24)
Stunted^b^	95 (54.9)	80 (46.0)
History of tuberculosis	58 (33.5)	39 (22.4)
Admitted for chest problems in last year	3 (1.7)	3 (1.7)
Current cough	13 (7.5)	18 (10.3)
Coughing up sputum^c^	7 (4.0)	17 (9.8)
Shortness of breath	5 (2.9)	1 (0.6)
Respiratory rate, mean (SD), breaths/min	22.2 (3.0)	22.6 (3.2)
Abnormal respiratory rate^d^	67 (38.7)	85 (48.9)
Oxygen saturation, mean (SD), %^a^	96.7 (3.0)	96.7 (2.4)
Oxygen saturation <92%	6 (3.5%)	11 (6.3%)
Heart rate, mean (SD), beats/min^a^	87.6 (12.5)	85.6 (11.6)
Abnormal heart rate^d^	6 (3.5%)	8 (4.6%)
Shuttle walk duration, mean (SD), min:s^a^	10:26 (1:56)	10:49 (2:03)

Abbreviations: AZM, azithromycin; FEV_1_, forced expiratory volume in 1 second; FVC, forced vital capacity; IQR, interquartile range.

^a^ Values were missing for currently attending school (1 patient in the AZM group and 3 patients in the placebo group), HIV viral load (2 patients in the AZM group), and FVC (3 patients in the AZM group and 5 patients in the placebo group).

^b^ Denotes a *z* score less than −2.

^c^ This question was asked for those with current cough only.

^d^ Age-defined cutoffs were based on data from O’Leary et al.^29^

### Study Medication

Study medication was stopped after a median (IQR) of 27 (10-38) weeks for 7 participants per protocol; 3 participants became pregnant, 3 participants had a prolonged QTc interval (2 in the placebo group and 1 in the AZM group; all 3 were asymptomatic with no history of cardiac disease), and 1 experienced a drug rash that was later determined to be unrelated to the trial drug. Adherence was higher in the AZM group than in the placebo group (127 participants [73.4%] vs 117 participants [67.2%]). AZM was not taken by any study participant during the study period, other than as a trial drug by participants in the intervention group.

### Follow-up and Outcomes

The primary outcome was assessed for 308 participants (88.8%), with fewer assessed in the placebo group than in the AZM group (146 participants [83.9%] vs 162 participants [93.6%]) (eTable 1 in Supplement 2). Baseline characteristics were similar between participants with and without primary outcome data, except that those without outcome data were more likely to be female and were slightly older (eTable 2 in Supplement 2).

The mean (SD) values of the primary outcome, FEV_1_ *z* score, were −1.90 (0.90) in the AZM group and −1.95 (0.91) in the placebo group. The AMD was 0.06 (95% CI using robust SEs, −0.10 to 0.21; *P* = .48) higher in the AZM group than in the placebo group, a nonsignificant difference. The test for the homoscedasticity assumption using the Cook-Weisburg test gave χ^2^_1_ = 6.4 and *P* = .01 before robust SEs were applied. On the prespecified per-protocol analysis, the AMD was 0.14 (95% CI, −0.02 to 0.29; *P* = .08); under prespecified sensitivity analysis excluding the 11 participants with baseline FEV_1_ *z* score greater than −1, the AMD was 0.07 (95% CI −0.08 to 0.23; *P* = .36). Under post hoc multiple imputation of outcome data, the AMD was 0.05 (95% CI, −0.11 to 0.20; *P* = .54).

**Figure 2.**
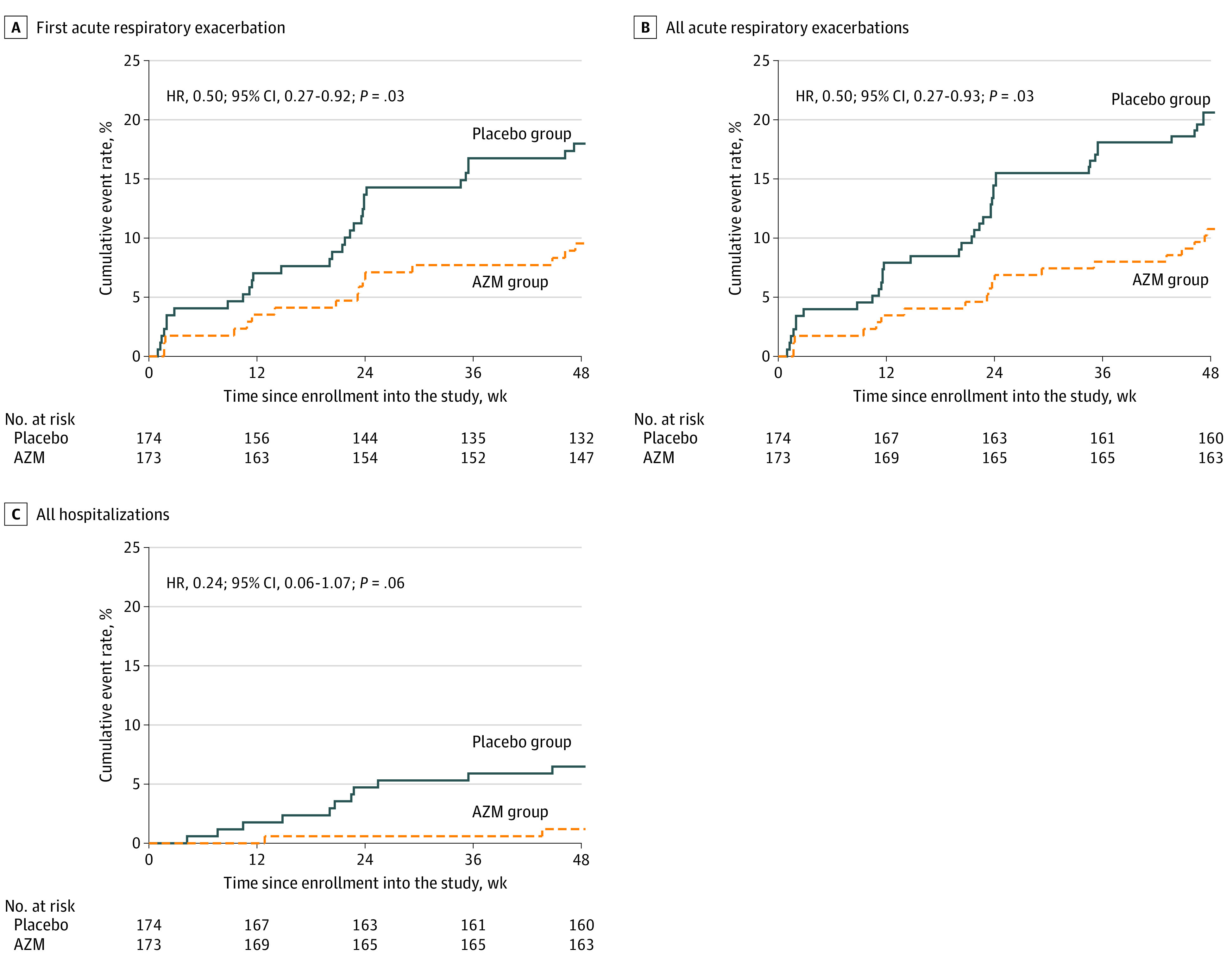
Cumulative Incidence of Time-to-Event Outcomes, Intention to Treat Analyses Graphs show data for first acute respiratory exacerbation (A), all acute respiratory exacerbations (B), and all-cause hospitalizations (C). AZM indicates azithromycin.

For assessment of secondary event outcomes, the median (IQR) follow-up period was 49 (49-49) weeks and the total follow-up time was 157 person-years in the AZM group and 154 person-years in the placebo group. AREs occurred in 16 participants (9.2%) in the AZM group (10.8 first events and 12.1 total events per 100 person-years) and 30 participants (17.2%) in the placebo group (21.7 first events and 24.7 total events per 100 person-years) (Figure 2A and B and Table 2). The hazard ratio in the AZM group compared with the placebo group for first ARE was 0.50 (95% CI, 0.27-0.92; *P* = .03) and the hazard ratio for all AREs was 0.50 (95% CI, 0.27-0.93; *P* = .03) (Table 2). The tests for the proportional-hazards assumptions with Schoenfeld residuals gave χ^2^_6_ = 7.0 and *P* = .32 for first ARE and χ^2^_6_ = 7.3 and *P* = .30 for all AREs.

**Table 2.** Outcome Measures (Primary and Secondary), Intention to Treat Analyses

End point	Participants, No./person-years, No.			*P* value	HR (95% CI)	*P* value
	AZM group (n = 173)^a^	Placebo group (n = 174)^b^	AMD (95% CI)			
Primary outcome, FEV_1_ *z* score at 48 wk, mean (SD)	−1.90 (0.90)	−1.95 (0.91)	0.06 (−0.10 to 0.21)	.48	NA	NA
Secondary outcomes^c^						
Total episodes of ARE	19/157	38/154	NA	NA	0.50 (0.27 to 0.93)	.03
First ARE	16/148	30/139	NA	NA	0.50 (0.27 to 0.92)	.03
Death	0/157	3/154	NA	NA	NA	NA
All-cause hospitalization	2/157	11/154	NA	NA	0.24 (0.06 to 1.07)	.06
*Salmonella typhi *infection	0/157	0/154	NA	NA	NA	NA
Gastroenteritis	1/157	2/154	NA	NA	NA	NA
Malaria	1/157	2/154	NA	NA	NA	NA
Weight-for-age *z* score, mean (SD)	−2.15 (1.38)	−1.94 (1.27)	0.03 (−0.08 to 0.14)	.56	NA	NA

Abbreviations: AMD, adjusted mean difference; ARE, acute respiratory exacerbation; AZM, azithromycin; FEV_1_, forced expiratory volume in 1 second; HR, hazard ratio; NA, not applicable.

^a^ Primary and secondary outcomes were assessed for 162 patients in the AZM group.

^b^ Primary and secondary outcomes were assessed for 146 patients in the placebo group.

^c^ Outcomes refer to number of participants per person-years of risk, unless stated otherwise. Formal comparison of trial groups was not undertaken if fewer than 10 events overall or no events in 1 trial group.

Hospitalizations occurred in 2 participants (1.2%) in the AZM group (1.3 total events per 100 person-years) and 9 participants (5.2%) in the placebo group (7.1 total events per 100 person-years) (Figure 2C and Table 2). The hazard ratio for hospitalizations was 0.24 (95% CI, 0.06-1.07; *P* = .06). Death, infectious episodes (*Salmonella typhi* and gastroenteritis), and malaria were rare (Table 2). There were 3 cases of tuberculosis, all in the placebo group (1 diagnosed through Xpert MTB/RIF, 1 diagnosed on chest radiography, and 1 patient with tuberculous meningitis who died). The mean (SD) values of weight-for-age *z* score were −2.15 (1.40) in the AZM group and −1.94 (1.27) in the placebo group. The AMD was 0.03 (95% CI using robust SE, −0.08 to 0.14; *P* = .56) higher in the AZM group compared with the placebo group.

**Figure 3.**
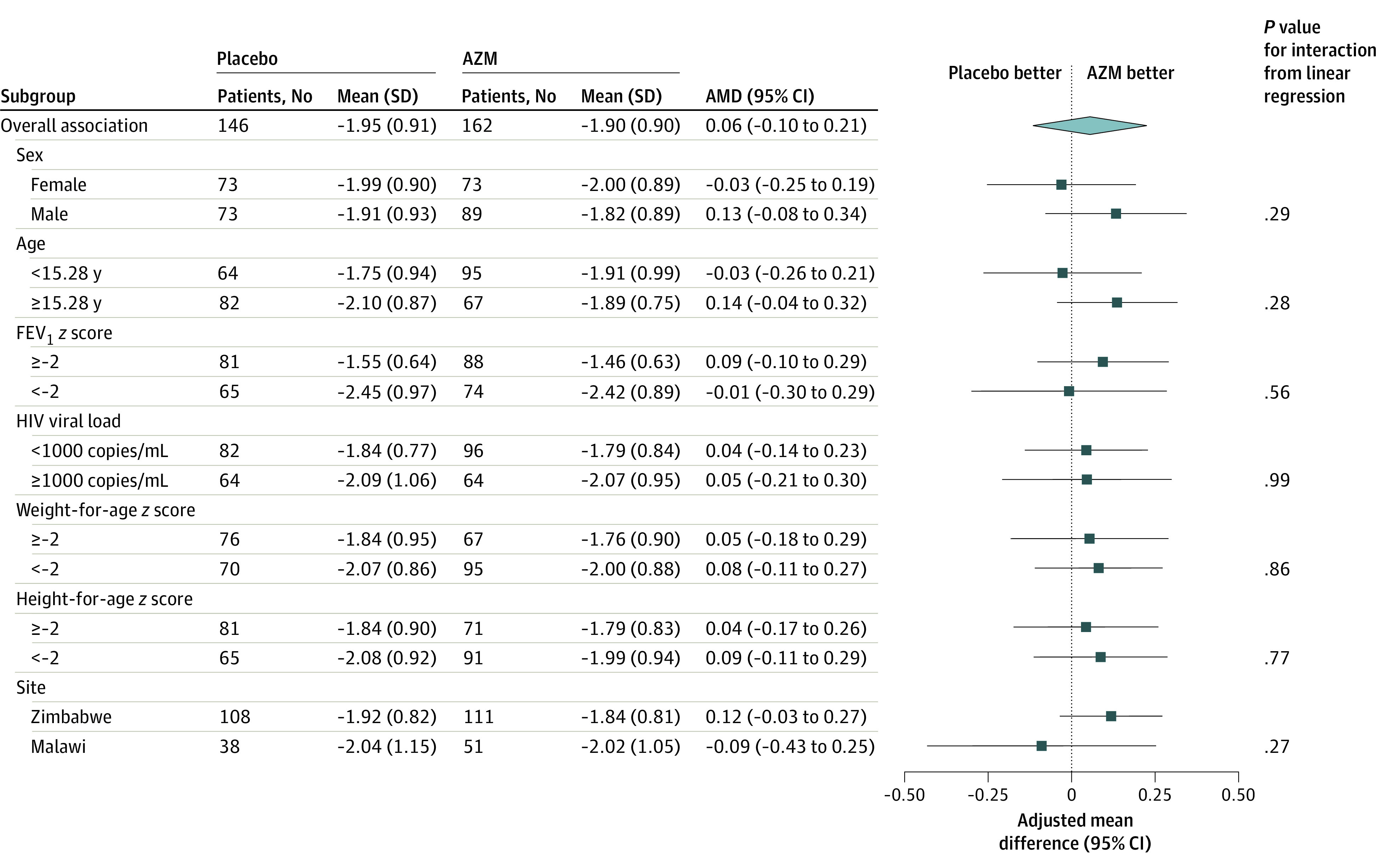
Intervention Effect (Adjusted Mean Difference [AMD]) for the Primary Outcome Overall and by Subgroups AZM indicates azithromycin; and FEV_1_, forced expiratory volume in 1 second.

The results of subgroup analyses for the primary outcome are shown in Figure 3 and in eTable 4 in Supplement 2. The results showed no evidence for treatment-by-subgroup interaction.

### Safety

There was a higher number of gastrointestinal symptoms in the AZM group compared with the placebo group, but they were all minor (DAIDS grade <3), transient, and resolved spontaneously. There were no cases of *C. difficile *infection*.* None of the severe adverse events was related to study medication (eTable 3 in Supplement 2).

## Discussion

This randomized clinical trial of an intervention to address HCLD in children found that once-weekly AZM had no effect on the primary outcome of pulmonary function, but it was associated with a reduced rate of AREs and increased time to first ARE compared with placebo. There were fewer hospitalizations in the AZM group vs the placebo group but the difference was not significant. We hypothesized that AZM would reduce systemic inflammation, which would translate into improvement of lung function, and/or have an antibiotic effect. Inflammatory and other biomarkers will be reported separately, but there was no evidence of improvement of pulmonary function in our trial. In previous studies,^30,31^ a decrease in airway inflammation with AZM was observed in 40% of patients with posttransplant OB, and inflammatory markers, including interleukin-8 and neutrophilia, were associated with improvements in FEV_1_. The effect of AZM on lung function has been variable, with no effect in patients with bronchiectasis (excluding cystic fibrosis) and an improvement at early time points (up to 6 months) but not beyond in studies of patients with cystic fibrosis.^18,32^

The prophylactic antimicrobial effect of AZM on ARE reported here for HCLD is consistent with trials of AZM in other chronic lung diseases among both children and adults, with a reduction in pulmonary exacerbations and less-frequent need for antibiotics compared with patients receiving placebo.^18,32^ Studies^2,3^ from sub-Saharan Africa in the ART era have shown that HCLD affects up to one-third of children; therefore, the present findings potentially have substantial implications in terms of reduced antimicrobial use. Given the lack of diagnostic facilities, children with chronic respiratory symptoms are often presumptively treated for tuberculosis, a common disease in high-HIV prevalence settings.^16,33^ Major indirect benefits, such as less interrupted schooling, improved quality of life, and reduced economic burden on the family, should also be evaluated.

Because of its broad-spectrum activity, AZM is also effective against pathogens that cause diarrhea and malaria, including *Salmonella* species, which are a major cause of death in children living with HIV.^34^ In trials evaluating mass drug administration of AZM for trachoma, there was a reduction in all-cause child mortality, likely through AZM’s cumulative effect on reducing the risk of serious infection from a range of pathogens.^35^ Data from our study showing reduced malaria, gastroenteritis, and deaths in the AZM group are consistent with a decreased risk of these diseases, but with low numbers of events. Because a mortality benefit is plausible, however, these data argue for additional studies with larger sample sizes and longer follow-up.

The number of severe adverse events was higher in the placebo group than in the AZM group and none was associated with AZM use. AZM was well-tolerated, with minor gastrointestinal adverse effects that resolved without specific treatment. Previous trials^18,19,20,36^ have also shown that AZM is remarkably safe, with nausea and diarrhea as the major adverse effects. A previous study^37^ reported a prolonged QTc interval among older patients with a high prevalence of cardiac disease, with potential for an estimated 0.047 additional deaths per 10 000 AZM courses. However, in the current study, of the 3 participants discontinued from study medication because of a prolonged QTc interval, 2 were in the placebo group and all 3 were asymptomatic with no history of cardiac disease. This reflects our conservative QTc interval threshold used for discontinuing study medication.

As well as being safe and well-tolerated, the pharmacokinetics of AZM are unique, with high intracellular uptake and slow hepatic excretion.^38,39^ The resulting high tissue concentrations made once-weekly dosing feasible, possibly reducing nonadherence.

A concern with AZM is the emergence of antimicrobial resistance in the airway microbiome. Studies^18,19,20,40^ have reported an increase in the proportion of macrolide-resistant commensal oropharyngeal organisms, including when AZM is given as a single dose as part of mass administration programs. Whether resistance persists and its clinical impact in individuals are not clear; notably, colonization with macrolide-resistant organisms has not been associated with increased exacerbations or a decline in pulmonary function.^41^ However, the potential of increase in macrolide AZM resistance in bystander organisms and increase in prevalence of macrolide-resistant organisms in the community is of public health concern.^41^ The potential for emergence of resistant organisms is under investigation and will be reported separately.

These concerns justify the need for a selective approach to treatment. Future trials should investigate AZM therapeutic effects against such factors as disease severity and pattern, functional impairment, and extent of immunocompromise to identify patients who would gain most from such an intervention.^42^ An additional question that remains to be answered is the sustainability of treatment effect. We had intended to investigate outcomes at 72 weeks to investigate the durability of treatment effect and persistence of resistance, but this was not feasible.

### Strengths and Limitations

The trial was well-powered, achieved high rates of follow-up, and had consistent findings across sensitivity analyses. The trial was conducted in public sector pediatric HIV clinics in 2 African countries, which contributes to generalizability. The intervention was delivered over a full year, thus reducing the seasonal factors associated with risk of ARE.

Limitations of the study include a higher loss to follow-up in the placebo than intervention group. However, multiple imputation was performed, and we adjusted for baseline factors associated with missing end line data. Spirometry is an operator-dependent procedure, and error in FEV_1_ measurement is possible. This was minimized through certified training and refresher training of research staff and quality control assessment of spirometric traces to ensure that American Thoracic Society standards were met. There was variance of FEV_1_ by age and potentially by trial group. Although robust SEs were used, heteroscedasticity may have affected primary outcome estimates. Furthermore, sustained adherence over a long period is challenging, particularly among the age group under study.

## Conclusions

Our trial found no difference in the primary outcome of lung function but showed that AZM is an effective intervention in reducing ARE events associated with HCLD in children and adolescents. AZM has previous proven efficacy for treating a variety of chronic lung diseases, is safe, and is well-tolerated. Future research should identify patient groups who would benefit most from this intervention, optimum treatment length and dosing schedules, and sustainability of treatment effect.

## Notes

Supplement 1. **Trial Protocol**

Supplement 2. **eAppendix.** Multiple Imputation Models **eTable 1.** Baseline Characteristics by Arm for Participants Analyzed for the Primary Outcome **eTable 2.** Baseline Characteristics Comparing Participants With and Without Primary Outcome **eTable 3.** Number of Adverse Events **eTable 4.** Intervention Effect (Adjusted Mean Difference) in Post Hoc Subgroup Analysis by Recruitment Date **eReference**

Supplement 3. **Data Sharing Statement**
