# Supplementary material for: Effect of Once-Weekly Azithromycin vs Placebo in Children With HIV-Associated Chronic Lung Disease: The BREATHE Randomized Clinical Trial
Source: JAMA Netw Open. 2020 Dec 17;3(12):e2028484. doi: 10.1001/jamanetworkopen.2020.28484 (PMC7747021; doi:10.1001/jamanetworkopen.2020.28484)
Supplement: Supplement 1. — Trial Protocol [file jamanetwopen-e2028484-s001.pdf]

UNIVERSITY OF TROMSO, LSHTM, LSTM, UNIVERSITY OF OXFORD, UNIVERSITY OF CAPE TOWN, BRTI, MLW CLINICAL RESEARCH PROGRAMME

Bronchopulmonary function in response to azithromycin treatment for chronic lung disease in HIV-infected children

(BREATHE)

Version 2.2 21st August 2017

General Information

Trial title: Bronchopulmonary function in response to azithromycin treatment for chronic lung disease in HIV-infected children.

Internal reference number/short title: BREATHE

Trial registration number: NCT02426112 (registered with clinicaltrials.gov)

Date and version number: 21st August 2017, Version 2.2

Chief Investigators: Jon Odland, Rashida Ferrand.

Investigators: Mark Nicol (Microbiologist), Sarah Rowland Jones (Immunologist), Helen Weiss, Elizabeth Corbett, Tore Guttenberg, Katharina Kranzer, Trond Flaegstad, Hilda Mujuru, Victoria Simms, Carmen Gonzalez

Sponsor: London School of Hygiene and Tropical Medicine (LSHTM)

Funder: Medical Research Council of Norway

Compliance:

The trial will be conducted in compliance with the protocol, ICH GCP Guidelines and other regulatory requirements applying in the countries in which the trial will be conducted.

Confidentiality:

This document contains confidential information that must not be disclosed to anyone other than the Sponsor, the Investigator Team, host organisation, and members of the Research Ethics Committee, unless authorised to do so.

Conflict interests:

We declare no conflict of interests.

Chief Investigator signatures:

Jon Odland **Rashida Ferrand**

Table of Contents

1 Main Trial Contacts 6

1.1 Site contacts 7

2 Executive summary 8

3 Synopsis 10

4 Abbreviations 12

5 Background and Rationale 13

5.1 Chronic Lung Disease in paediatric HIV infection 13

5.2 Pathogenesis of HIV-associated chronic lung disease 13

5.3 The use of azithromycin in treating chronic lung disease 14

5.4 Study Rationale 14

5.5 Hypothesis & Approach 15

6 Objectives and Outcome measures/Endpoints 15

7 Trial design 17

8 Participant identification 17

8.1 Trial participants 17

8.2 Inclusion criteria 17

8.3 Exclusion criteria 17

8.4 Enrolment of comparison group participants 18

9 Trial Procedures 18

9.1 Recruitment 18

9.2 Informed consent 19

9.3 Screening and eligibility assessment 19

9.4 Randomisation, blinding and code-breaking 19

9.5 Assessment of trial outcomes 20

9.5.1 Primary and secondary outcomes: 20

9.5.2 Laboratory sub-studies 20

9.5.3 Cardiac sub-study 21

9.6 Baseline assessments and follow up visits 22

9.6.1 Schedule of follow-up and investigations in trial participants 22

9.6.2 Project plan design 23

9.6.3 Investigation of acute exacerbations (or infections) and adverse events 24

9.6.3 Non-CLD Comparison group 24

9.7 Discontinuation/Withdrawal of participants from trial treatment 24
9.8 Definition of end of trial 25
10 Investigational Medicinal Product 25
10.1 IMP description 25
10.1.1 Azithromycin 25
10.1.2 Placebo 25
10.2 Storage IMP 25
10.3 Compliance of the trial treatment 25
10.4 Accountability of trial treatment 25
10.5 Dispensing 26
10.6 Concomitant medication 26
10.7 Post-trial treatment 26
11 Safety Reporting 26
11.1 Definitions 26
11.1.1 Known important/frequent Azithromycin side effects 27
11.2 Causality 29
11.3 Procedures for recording adverse events 29
11.4 Procedures for reporting serious adverse events 29
11.5 Safety Monitoring Committee 30
11.6 Pregnancy 30
12 Statistical Considerations 30
12.1 Sample size justification 30
12.2 Data analysis plan 32
12.3 Microbiome and Immunology analysis 32
13 Data Management 32
13.1 Source Data 32
13.2 Access to data 33
13.3 Data recording and record keeping 33
13.4 Data cleaning and validation 33
14 Trial Monitoring 33
15 Ethical Considerations and Approval 34
16 Regulatory approval 35
17 Inspection and Audits 35
18 Finance and Insurance 35

18.1 Funding 35
18.2 Insurance 35
19 Publication 35
20 References 37
21 Appendices 40
21.1 Toxicity Grading (DAIDS) 40

1 Main Trial Contacts

Chief Investigators

Jon Odland
Address: Department of Community Medicine Faculty of Health Sciences The Arctic University of Norway, N-9037 Tromsø
Telephone: +4790953887
Fax: +4777644831
Email: jon.oyvind.odland@uit.no

Rashida Ferrand
Address: London School of Hygiene and Tropical Medicine, Keppel Street, London WC1E 7HT
Telephone: +44 7708 655 043
Fax: +44 207 612 7860
Email: rashida.ferrand@lshtm.ac.uk

Sponsor

London School Of Hygiene and Tropical Medicine
Address: Keppel Street, London WC1E 7HT
Telephone: +44 207 927 2626
Email: Patricia.Henley@lshtm.ac.uk

Clinical Trials Unit

London School of Hygiene and Tropical Medicine
Address: Keppel Street, London WC1E 7HT
Telephone: +44 207 927 2626
Email: Patricia.Henley@lshtm.ac.uk

Statistician

Andrea Rehman
Address: London School of Hygiene and Tropical Medicine, Keppel Street London WC1E 7HT
Telephone: +44 207 927 2132
Email:andrea.rehman@lshtm.ac.uk

Committees

Data Safety and Monitoring Board (DSMB)

Professor Christopher Whitty (Chair)
London School of Hygiene & Tropical Medicine
Email: Christopher.whitty@lshtm.ac.uk

Professor Sarah Walker
University College London
Email: rmjlasw@ucl.ac.uk

Professor Steve Graham
University of Melbourne
Email:steve.graham@rch.org.au

Trial Steering Committee (TSC)

Professor Liz Molyneux (Chair)
College of Medicine, Blantyre
Email: emmolyneux@gmail.com

Professor Heather Zar
University of Cape Town
Email: heather.zar@uct.ac.za

Dr Lynda Stranix-Chibanda
University of Zimbabwe-UCSF collaboration
lynda@uz-ucsf.co.zw

Professor Catherine Hankins
Amsterdam Institute for Global Health & Development AND London School of Hygiene & Tropical Medicine
Email: Catherine.hankins@lshtm.ac.uk

Ms Amanda Madyadi
University of Zimbabwe
Email: amandamadyadi@gmail.com

1.1 Site contacts

**Zimbabwe**	
**PI**	**Rashida Ferrand**<br>*Address*: Biomedical Research and Training Institute, 10 Seagrave Road, Harare, Zimbabwe<br>*Telephone*: +263 (4) 333091<br>*Email*: rashida.ferrand@lshtm.ac.uk
**Trial coordinator**	**Ethel Dauya**<br>*Address*: Biomedical Research and Training Institute, 10 Seagrave Road, Harare, Zimbabwe<br>*Telephone*: +263 7744 52172<br>*Email*: edauya@brti.co.zw

**Malawi**	
**PI**	**Carmen Gonzalez**<br>*Address*: Malawi-Liverpool-Wellcome Trust Clinical Research Programme. P.O Box 30096, Chichiri, Blantyre 3. Malawi.<br>*Telephone*: +265 (0) 1876444<br>*Fax*: +265 (0) 1875774<br>*Email*: Carmen.Gonzalez@lstmed.ac.uk
**Trial coordinator**	**Carmen Gonzalez**<br>*Address*: Malawi-Liverpool-Wellcome Trust Clinical Research Programme. P.O Box 30096, Chichiri, Blantyre 3. Malawi.<br>*Telephone*: +265 (0) 1876444<br>*Fax*: +265 (0) 1875774<br>*Email*: Carmen.Gonzalez@lstmed.ac.uk

**Cape Town (Microbiology)**	
**PI**	**Mark Nicol**<br>*Address*: Faculty of Health Sciences, Division of Clinical Microbiology, Anzio Road, Falmouth building, Entrance 2, level 5, room 5.28. University of Cape Town, South Africa<br>*Telephone*: +27 21 4066083<br>*Fax*: +27 21 4066210<br>*Email*: Mark.Nicol@uct.ac.za

**Oxford (Immunology)**	
**PI**	**Sarah Rowland-Jones**<br>*Address*: NDM Research Building, Nuffield Department of Medicine, University of Oxford, Old Road Campus, Roosevelt Drive, Oxford, OX3 7FZ, UK.<br>*Telephone*: +44 (0) 1865 612907 (SRJ)/ +44 (0) 1865 612912 (LMY)<br>*Email*: sarah.rowland-jones@ndm.ox.ac.uk/louis-marie.yindom@ndm.ox.ac.uk

2 Executive summary

Chronic pulmonary disease (CLD) is the most common manifestation of HIV/AIDS among children, accounting for more than 50% of HIV-associated mortality. Recently, a novel form of CLD, affecting more than 30% of African HIV-infected older children was described by Ferrand *et al* in Zimbabwe, high-resolution CT scanning findings showed predominantly small airways disease consistent with constrictive obliterative bronchiolitis (OB). OB is a life-threatening condition that results from small airways inflammation and fibrosis, can progress to hypoxic respiratory failure and cor pulmonale, and may impair lung growth in children. The aetiology of OB is incompletely understood but is thought to be due to an interaction of immune-mediated mechanisms and processes such as infection or ischaemia, leading to tissue injury followed by aberrant fibro-proliferative remodelling in the small airways. HIV infection results in chronic systemic immune activation and this is believed to be a key mechanism of pathogenesis of several chronic complications of HIV and may also predispose to CLD. Azithromycin has anti-inflammatory activity and treatment of CLD with this agent may lead to suppression of generalized immune activation.

This specific aims of this project are to:

1: Primary objective: To investigate whether adjuvant treatment with azithromycin results in improvement in lung function in HIV-infected children on ART for at least six months with chronic lung disease..

2: **Secondary objectives:**

a) To investigate the intervention effect on exacerbations of lung disease and morbidity
b) To investigate adverse events related to azithromycin treatment

3: Sub-studies:

Laboratory

a) To determine the effect of azithromycin therapy on antimicrobial resistance in bacteria colonizing the respiratory tract.
b) To investigate the diversity and composition of the respiratory and gut microbiome in HIV-infected children with and without CLD, and by trial arm.
c) To investigate the levels of biomarkers between those with and without CLD and the effect of azithromycin on biomarkers of systemic inflammation in HIV-infected children with CLD.

Cardiac

a) Describe the cardiac symptoms and echocardiograph findings of HIV-infected children on ART for at least six months with chronic lung disease,
b) Determine the prevalence of right-sided cardiac dysfunction and/or pulmonary hypertension in HIV-infected children with chronic lung disease, who are stable on antiretroviral therapy, determined by echocardiography at baseline.
c) To investigate whether adjuvant treatment with azithromycin results in improvement in right-sided cardiac function and/or pulmonary hypertension in HIV-infected children on ART for at least six months with chronic lung disease, , determined by echocardiography at 12 months.

In total, 400 children aged 6-19 years, living with HIV and diagnosed with CLD will be enrolled at Harare Children´s Hospital in Harare (Zimbabwe) and Queen Elizabeth Central Hospital in Blantyre (Malawi). These will receive weekly treatment with azithromycin or placebo during 12 months. Another 100 children living with HIV but with no CLD will be enrolled as a comparison group for laboratory sub-studies.

Lung function will be assessed using spirometry and the Forced Expiratory Volume in the first second (FEV1) will be the primary outcome. The mean FEV1 z-score will be compared between trial arms 12 months after initiation of azithromycin treatment.

Results will help in the evidence-based management of these patients and will give insight of the pathogenesis of CLD. Results will be disseminated to relevant national and international stakeholders and for publication in peer review journals.

3 Synopsis

Trial Title	Broncho-pulmonary function in response to azithromycin treatment for chronic lung disease in HIV-infected children.	
Internal ref. no. (or short title)	BREATHE	
Clinical Phase	III	
Trial Design	Multi-site, individually randomised, double-blinded, placebo-controlled trial of weekly azithromycin for 12 months	
Trial Participants	Children aged 6-19 years living with HIV and with diagnosis of chronic lung disease. Another 100 children living with HIV but with no chronic lung disease in a comparison arm.	
Planned Sample Size	400 cases and 100 in the comparison arm	
Treatment duration	12 months	
Follow up duration	12-18 months	
Planned Trial Period	September 2015-September 2019	
	**Objectives**	**Outcome Measures/Endpoints**
Primary trial outcome	To investigate whether adjuvant treatment with azithromycin results in improvement in lung function in HIV-infected children on ART with chronic lung disease,	Compare between trial arms<br>Mean (sd) Forced Expiratory Volume in one second z score (FEV1z) after 12 months of initiation of the trial drug
Secondary trial outcomes	To investigate the intervention effect on exacerbations of lung disease and morbidity<br><br>To investigate adverse events related to azithromycin treatment	Compare between trial arms<br>a) Mean FEV1 z-score 18 months after treatment initiation with azithromycin<br>b) Time to death<br>c) Time to first acute exacerbation<br>d) No of hospitalisations by 12 and 18 months after azithromycin initiation<br>e) Number of exacerbations by 12 and 18 months after azithromycin initiation<br>f) Mean (sd) weight-for-age z-score by 12 and 18 months<br>*g)* No. of mild, moderate and severe adverse events<br>*h)* Incidence of Malaria episodes (Malawi only) in the 12 months after treatment initiation<br>*i)* Incidence of blood stream infections due to *Salmonella typhi and non-typhi* in the 12 months after treatment initiation<br>*j)* Incidence of gastroenteritis episodes in the 12 months after treatment initiation

Cont.	Objectives	Outcome Measures/Endpoints
Laboratory sub-studies	To determine the effect of azithromycin therapy on antimicrobial resistance in bacteria colonizing the respiratory tract	Prevalence of colonization with macrolide (and multidrug-resistant) *Streptococcus pneumoniae*, *Staphylococcus aureus* and *Haemophilus influenzae* in the two trial arms at 12 months after initiation of therapy with azithromycin
	To investigate the diversity and composition of the respiratory microbiome in HIV-infected children with and without CLD, and by trial arm in those with CLD	Composition and diversity of the respiratory bacterial microbiome (determined by culture of clinically relevant organisms and sequencing of 16s rRNA gene amplicons) at 12 months by trial arm AND between those with and without CLD at baseline
	To investigate the diversity and composition of the gut microbiome in HIV-infected children with and without CLD, and by trial arm in those with CLD	Composition and diversity of the gut bacterial microbiome (determined by culture of clinically relevant organisms and sequencing of 16s rRNA gene amplicons) by trial arm AND between those with and without CLD at baseline
	To investigate the effect of azithromycin on biomarkers of systemic inflammation in HIV-infected children with CLD and levels of biomarkers in those with and without CLD	Association between biomarker levels and FEV1 z-score; Comparison of levels of biomarkers of systemic and pulmonary inflammation between the two trial arms at 12 and 18 months after initiation of therapy with azithromycin
Cardiac sub-study	Describe the cardiac symptoms and echocardiograph findings of HIV-infected children on ART with chronic lung disease,<br><br>Determine the prevalence of right sided cardiac dysfunction and/or pulmonary hypertension in HIV-infected children on ART with chronic lung disease, determined by echocardiography at baseline<br><br>To investigate whether adjuvant treatment with azithromycin results in improvement in right-sided cardiac function and/or pulmonary hypertension in HIV-infected children on ART with chronic lung disease, determined by echocardiography at 12 months	a) Baseline echocardiographic parameters<br>b) Baseline prevalence of right sided cardiac dilatation and dysfunction<br>c) Prevalence of right sided cardiac dilatation and dysfunction at 12 months after initiation of therapy with azithromycin between the two trial arms

Investigational Medicinal Product(s)	Azithromycin<br>Placebo
Formulation	Tablets 250 mg
Dose	According to weight bands (30 mg/kg/week):<br>• 10-19.9 kg: 250 mg<br>• 20-29.9 kg: 500 mg<br>• 30-39.9 kg: 750 mg<br>• ≥40 kg: 1250 mg
Route of Administration	Oral

4 Abbreviations

AIDS	Acquired immune deficiency syndrome
AE	Adverse Event
ART	Anti-retroviral therapy
BRTI	Biomedical Research and Training Institute
CAG	Community advisory group
CLD	Chronic lung disease
CTU	Clinical Trials Unit
CoM	College of Medicine
DOT	Direct observed therapy
ECG	Electrocardiogram
FEV1	Forced expiratory volume in the first second
FVC	Forced vital capacity
HIV	Human immunodeficiency virus
LIP	Lymphocytic interstitial pneumonitis
LSHTM	London School of Hygiene and Tropical Medicine
LSTM	Liverpool School of Tropical Medicine
MLW	Malawi-Liverpool-Wellcome Trust
NTM	Non-tuberculous mycobacteria
OB	Obliterative bronchiolitis
RCT	Randomised controlled trial
SAR	Serious adverse reaction
SAE	Serious adverse event
SUSAR	Suspected unexpected serious adverse reaction
TB	Tuberculosis

5 Background and Rationale

5.1 Chronic Lung Disease in paediatric HIV infection

Chronic pulmonary disease is the most common manifestation of HIV/AIDS among children, accounting for more than 50% of HIV-associated mortality.[1,2] As children get older, they have an increased risk of developing HIV-associated chronic lung disease (CLD).[3] In the pre-ART era, the most common cause of CLD was lymphocytic interstitial pneumonitis (LIP) found in 30-40% of HIV-infected children.[4,5] LIP responds well to ART and the prevalence has significantly declined with increased availability of ART.[6]

Recently, a novel form of CLD, affecting more than 30% of African HIV-infected older children was described by Ferrand *et al* in Zimbabwe.[7] Children classically present with a chronic cough (often leading to presumptive treatment for tuberculosis), hypoxia and significantly reduced exercise tolerance.[7] Similar findings were found in a follow-up study in Malawi.[8] Unexpectedly, high-resolution CT scanning findings showed predominantly small airways disease consistent with constrictive obliterative bronchiolitis (OB), with LIP being an exceptional finding.[9] In both studies, even participants with pronounced respiratory impairment looked well at rest, not all had cough, and plain radiological abnormalities were subtle, consistent with OB.[10] Importantly, no association was observed between abnormal lung function, ART use or duration or CD4 count in either study, suggesting that this form of HIV-related CLD, unlike LIP, may not be responsive to ART. Notably, nearly half of ART-naïve participants with CD4 counts that did not meet criteria for taking ART also had CLD and 12% of patients in the Zimbabwe series had co-existing pulmonary hypertension.[7]

OB is a life-threatening condition that results from small airways inflammation and fibrosis, can progress to hypoxic respiratory failure and cor pulmonale, and may impair lung growth in children.[10] OB has not been recognized in Western cohorts of HIV-infected children or in adults with longstanding infection. Possible reasons include genetic susceptibility,[11] environmental variability (e.g. exposure to particulate matter), pathogen milieu, low rates of vaccine-preventable diseases, and early treatment with ART (since infancy).[11]

5.2 Pathogenesis of HIV-associated chronic lung disease

The aetiology of OB is incompletely understood but is thought to be due to an interaction of immune-mediated mechanisms and processes such as infection or ischaemia, leading to tissue injury followed by aberrant fibro-proliferative remodelling in the small airways.[12] HIV infection results in chronic systemic immune activation, and this is believed to be a key mechanism of pathogenesis of several chronic complications of HIV and may also predispose to CLD.[13-15] The underlying mechanism of immune activation in HIV is an increase in gut permeability leading to the translocation of microbial products (such as lipopolysaccharide), which leads to chronic activation of circulating monocytes.[14] As detailed below, azithromycin has anti-inflammatory activity and treatment of CLD with this agent may lead to suppression of generalized immune activation, assessed using specific plasma biomarkers, as well as a fall in pulmonary inflammation assessed by the levels of sputum neutrophil content.[16]

In addition, studies suggest that the specific composition of the lower respiratory tract microbiome may influence the development or progression of CLD.[17-21] This may be due to a direct effect of microbial products or through the role of microorganisms in maintaining immune homeostasis, but raises therapeutic modulation of the airway microbiota as a potential therapeutic option for patients with inflammatory airway disease.[21] Recent data show clear differences between the lung microbiota in HIV-infected and uninfected individuals,[22,23] Therefore, an altered respiratory microbiome in HIV-infected children may play a key role in the development and progression of CLD, and therapies which affect this microbiome may modulate disease outcome.

5.3 The use of azithromycin in treating chronic lung disease

Azithromycin is an orally available macrolide antibiotic with broad-spectrum activity. In addition, macrolides exhibit anti-inflammatory and immunomodulatory properties. Azithromycin has shown a significant therapeutic and anti-inflammatory effect in several chronic lung diseases.[24-31] It has unique pharmacokinetic properties with considerable intracellular uptake and slow hepatic excretion, resulting in a long half-life which enables infrequent dosing.[32-34] In a meta-analysis of four randomised controlled trials (RCT) in patients with cystic fibrosis, use of azithromycin was associated with significant improvement in lung function, reduction in pulmonary exacerbations, less frequent need for oral antibiotics compared to patients receiving placebo.[28] Three RCTs in patients with non -cystic fibrosis bronchiectasis showed similar findings.[24-26] Observational studies also show a benefit in OB in the context of lung transplantation, but trials are lacking.[35,36] In HIV-infected individuals, azithromycin resulted in a 61% reduction in *Mycobacterium avium* complex infections.[37] In African children, periodic mass drug administration of azithromycin used for trachoma control has been associated with unexpected benefits on child survival: with a 50% reduction in mortality in children aged 1-9 years reported from Ethiopia. A lasting benefit may be attributable to action against major lower respiratory tract pathogens, and some bacterial pathogens that cause diarrhoea.[38,39]

Azithromycin is remarkably safe. Some trials report nausea and diarrhoea as the major side-effects, although this is not a consistent finding.[24,26-28,40,41] A recent study reported a prolonged QTc interval from older patients with a high prevalence of cardiac disease, with potential for an estimated 0.047 additional deaths per 10,000 azithromycin courses.[42] A major concern with using azithromycin, however, is the emergence of new pathogens and antimicrobial resistance among the airway microbiome. While the former was not found in studies,[24,26,27] there was a significant increase in the proportion of macrolide-resistant commensal oropharyngeal organisms.[24,26-28,43]

5.4 Study Rationale

Despite efforts to eliminate mother to child transmission, 240,000 infants were newly infected with HIV at the end of 2013 globally, and the coverage of antiretroviral therapy (ART) in children was only 24%.[44,45] Thus, HIV will continue to place a heavy burden on paediatric clinical services in sub-Saharan Africa, where 90% of the world's HIV-infected children live.[46] Hitherto, the main focus of HIV programs has been on meeting the need for massive scale-up of paediatric ART and on improving infant survival. Now, attention needs to shift to the growing cohort of older HIV-infected survivors, a group that has been almost entirely neglected until the last few years.[47,48] If our finding, that over a third of older African children in HIV care have CLD, is also applicable to children growing up in the era of early infant

diagnosis and care, then the urgency with which evidence-based management guidelines are needed becomes starkly apparent: this is a very common condition for which no evidence base exists to guide management. Our group has investigated bronchodilators, and short course, high-dose, steroids in patients with CLD who are on ART and isoniazid preventive therapy, with no suggestions of benefit.[49] Unlike in younger age groups, CLD in older HIV-infected children does not appear to respond to ART. Proceeding to a Phase III RCT of azithromycin to treat CLD in children living with HIV is justified because of the need for prolonged ART treatment before benefit is anticipated, the minimal potential for harm, and the unusually strong *a priori* case for likely benefit from azithromycin outlined above.

5.5 Hypothesis & Approach

We propose a randomised clinical trial of adjuvant treatment with azithromycin to improve symptoms and lung function in HIV-infected children who are on ARTfor at least six months.. Azithromycin is the obvious choice due to its dual anti-inflammatory and antibiotic properties, demonstrated efficacy in treating other chronic lung diseases, and its tolerability. The potential for emergence of resistance will be investigated together with sub-studies that will contribute to our understanding of the aetiology and long-term progression of this important emerging condition.

6 Objectives and Outcome measures/Endpoints

Objectives	Outcomes
Primary Objective	
a) To investigate whether adjuvant treatment with azithromycin results in improvement in lung function in HIV-infected children on ART with chronic lung disease	_Primary Outcome_ compare trial arms<br>Mean (sd) Forced Expiratory Volume in one second z score (FEV1z) after 12 initiation of months of therapy with azithromycin
Secondary Objective	
To investigate the intervention effect on mortality, exacerbations of lung disease, morbidity<br><br>To investigate adverse events related to azithromycin treatment	_Secondary outcomes:_ compare trial arms<br>Mean FEV1z 18 months after treatment initiation with azithromycin<br>Time to death<br>Time to first acute exacerbation<br>No of hospitalisations by 12 and 18 months after azithromycin initiation Number of exacerbations by 12 and 18 months after azithromycin initiation<br>Mean (sd) weight-for-age z-score by 12 and 18 months<br>No. of mild, moderate and severe adverse events<br>No. of Malaria episodes (Malawi only) at 12 months after treatment initiation<br>No. of blood stream infections due to *Salmonella typhi and non-typhi* at 12 months after treatment initiation<br>No. of gastroenteritis episodes at 12 months after treatment initiation

Objectives	Outcomes
Laboratory sub- studies	
a) To determine the effect of azithromycin therapy on antimicrobial resistance in bacteria colonizing the respiratory tract	Prevalence of colonization with macrolide (and multidrug) -resistant) *Streptococcus pneumoniae*, *Staphylococcus aureus* and *Haemophilus influenzae* in the two trial arms at 12 months of initiation of treatment with azithromycin
b) To investigate the diversity and composition of the respiratory microbiome in HIV-infected children with CLD, specifically:<br>i. whether the microbiome differs between children with and without CLD<br>ii. whether azithromycin has an effect on the microbiome<br>iii. whether response to azithromycin is associated with specific patterns of microbial colonization in the respiratory tract	Composition and diversity of the respiratory bacterial microbiome (determined by culture of clinically relevant organisms and sequencing of 16s rRNA gene amplicons) in:<br>i. HIV-infected children with and without chronic lung disease (baseline)<br>ii. Children in azithromycin and placebo groups (12 and 18 months)<br>iii. Children receiving azithromycin therapy who do and do not show improvement in lung function (12 and 18 months)
c) To investigate the diversity and composition of the gut microbiome in HIV-infected children with CLD, specifically:<br>i. whether the microbiome differs between children with and without CLD<br>ii. whether azithromycin has an effect on the microbiome	Composition and diversity of the gut bacterial microbiome (determined by culture of clinically relevant organisms and sequencing of 16s rRNA gene amplicons) in:<br>i. HIV-infected children with and without chronic lung disease (baseline)<br>ii. Children in azithromycin and placebo groups (12 and 18 months)
d) To investigate the effect of azithromycin on biomarkers of systemic inflammation in HIV-infected children with CLD, specifically:<br>i. the correlation between extent of systemic inflammation and CLD severity<br>ii. whether azithromycin results in improvement in biomarkers of systemic and pulmonary inflammation	Association between biomarker levels and FEV1 z-score<br>i. Comparison of biomarker levels among children with and without CLD<br>ii. Comparison of levels of biomarkers of systemic and pulmonary inflammation between the two trial arms at 12 and 18 months after initiation of azithromycin therapy
Cardiac sub-study	
a) Describe the cardiac symptoms and echocardiograph findings of HIV-infected children on ART with chronic lung disease	Baseline echocardiographic parameters
b) Determine the prevalence of right sided cardiac dysfunction and/or pulmonary hypertension in HIV-infected children on ART with chronic lung disease, , determined by echocardiography at baseline	Baseline prevalence of right sided cardiac dilatation and dysfunction
c) To investigate whether adjuvant treatment with azithromycin results in improvement in right sided cardiac function and/or pulmonary hypertension in HIV-infected children on ART with chronic lung disease, determined by echocardiography at enrolment and 12 months.	Prevalence of right sided cardiac dilatation and dysfunction at 12 months after initiation of azithromycin therapy by intervention arm

7 Trial design

A multi-site, individually randomised, double-blinded, placebo-controlled trial of weekly azithromycin for 12 months with follow-up up to 18 months after enrolment in 400 children living with HIV with CLD. Recruitment will be done over a 24-month period, participants recruited during the first 12 months will be followed up during 18 months and participants recruited during the last 12 months will be followed up for 12 months. An additional 100 children living with HIV but with no CLD will be enrolled in a comparison group, with a total study sample of 500 children. Recruitment will be done to achieve the 400 cases across sites.

8 Participant identification

8.1 Trial participants

Children aged 6-19 years and resident in greater Harare (Zimbabwe site) and within Blantyre District (Malawi site) , to be recruited in the following sites:

1. Harare Children's Hospital HIV Clinic, Harare, Zimbabwe
2. Queen Elizabeth Hospital and all primary Health Centres within Blantyre District, Malawi

8.2 Inclusion criteria

1. Diagnosis of chronic lung disease (defined as FEV1 z-score <-1.0 and lack of reversibility with salbutamol (<12% improvement in FEV1 after administration of salbutamol). The z-score will be derived using GLI reference ranges.
2. Age 6-19 years
3. Perinatally-acquired HIV infection the most likely source of transmission
4. On first or second-line ART for at least 6 months
5. A firm home address accessible for visiting and intending to remain there for 18 months
6. Willing to agree to participate in the study and to give samples of blood, stool and sputum
7. Defined and stable guardian or care-giver. For participants aged over 18 years having a guardian will be not required.
8. HIV status disclosed to child for those aged older than 12 years (13-17 years)

8.3 Exclusion criteria

1. Any condition (except HIV) that may prove fatal during the study period (e.g. malignancy, end-stage HIV disease or other conditions deemed likely fatal by the trial physician)
2. Diagnosis of active pulmonary TB
3. Pregnant or breast-feeding
4. Condition likely to lead to lack of understanding of study procedures or to uncooperative behaviour e.g. neurocognitive disease, developmental delay or psychiatric illness
5. History of prolonged QTc syndrome or current or planned therapy with drugs likely to cause cardiac dysrhythmias

6. Prolonged QTc demonstrated after ECG performance.
7. Acute respiratory tract infection during enrolment (patients will be eligible once their acute infection is treated)
8. Creatinine clearance of <30mls/minute
9. ALT more than 2 times the upper limit of normal
10. History of cholestatic jaundice or hepatic dysfunction associated with previous use of azithromycin
11. Known hypersensitivity to azithromycin, erythromycin or any macrolide or ketolide drug.
12. Concomitant prolonged use of digoxin and/or fluconazole
13. Children living in the same household as a trial participant (to avoid inadvertent mix-up of trial drugs)

Children who have undergone screening with spirometry can be re-screened in the following cases:

- Were unable to provide a good quality spirometric output at previous screening
- Children screened according to the previous protocol version and found ineligible due to high viral load and z score >-1.64 and <-1.0 may be re-screened.

8.4 Enrolment of comparison group participants

A comparison group of 100 participants who meet all of the inclusion criteria for trial participants *except chronic lung disease* (defined as FEV1 z-score ≥ 0 SD and no clinical symptoms of acute or chronic respiratory pathology) will be eligible for inclusion in a separate group specifically for the laboratory sub studies. These participants will not be randomized into the trial, but will be asked to provide an induced sputum sample, NP swabs and stool sample/rectal swab for microbiological investigations and a blood sample for immunological studies at enrolment. As these participants will not be taking any medication, screening for eligibility to take drugs or TB screening will not be performed.

Participants will be frequency matched in a 1:1 in Malawi and 1:2 in Zimbabwe ratios of comparison: cases of CLD, according to age ranges (6-9 years; 10-12 years; 13-16 years; 17-19 years) and duration of ARV treatment of cases (6 months-2 years; 2-4 years; 4-6 years).

9 Trial Procedures

9.1 Recruitment

Potentially eligible children will be identified by, or referred to, study team members at the study site clinics.

9.2 Informed consent

Caregivers of potentially eligible children will be approached and informed about the study (verbally and by giving a written information sheet with the study details). Interested parents or guardians will be asked for written permission for the child to participate. All children will be asked for written assent to participate with two different information sheets depending on the age of the child. For less than 13 years of age a simplified information sheet will be used not mentioning HIV to avoid accidental disclosure, for children over 12 years (13-17 years) knowing HIV status is an inclusion criteria so a more comprehensive information sheet will be used talking openly about HIV.

Participants aged over 18 years will be asked for written permission directly and the presence of a caregiver will be not compulsory in this case.

9.3 Screening and eligibility assessment

Perinatally HIV-infected patients known to be on ART ≥ 6 months will be invited to be screened for inclusion in the trial. Perinatal HIV infection will be accepted as the most likely source of HIV if the following case definitions are met; these include self-report of no sexual debut or blood transfusions, a history of orphanhood due to maternal HIV disease and/or of a sibling death due to HIV, and characteristic clinical features (≥1 of stunting, history of recurrent minor infections (skin upper respiratory tract) in childhood, and planar warts).

A multi-step screening procedure will be performed. The Forced Oscillation Technique (FOT) is a non-invasive, non-operator dependent technique that could be used as a pre-screening test before spirometry is performed to confirm eligibility. If the child meets the case definition of CLD (FEV1 z-score < -1.0) further tests will be performed including a urine pregnancy test (for females who have reached menarche), a serum creatinine and ALT, an ECG and screening for TB. For TB screening one sputum sample will be obtained (either spontaneously or through induction) and tested using Gene Xpert.

9.4 Randomisation, blinding and code-breaking

Eligible patients will be randomly assigned to receive either Azithromycin or placebo in a 1:1 ratio, by block randomisation with variable length blocks stratified by site (Zimbabwe, Malawi). Block length will vary between block sizes 2, 4, 6, 8, 10 distributed in proportion to elements of Pascal's triangle, i.e. in the ratio 1:4:6:4:1, using the ralloc command in Stata. There will be no further stratification applied. The randomisation schedule and allocation list will be generated using Stata, by an independent statistician based in London. The allocation list will be sent directly to the pharmacist who will prepare the study medication. Participants and study personnel (including the site data managers and the trial statistician) will therefore be masked to treatment allocation. The pharmacist will use randomly- allocated serial numbers to prepare the correctly dosed study medication and will thus also remain blinded. The allocation of all grade 4 SARs or SUSARs will be unmasked and discussed by the trial management group and the DSMB. Unmasking of grade 3 SAR will be considered individually by the trial management group.

9.5 *Assessment of trial outcomes*

9.5.1 Primary and secondary outcomes:

FEV1 will be measured using spirometry. The baseline spirometry will be performed at screening. The spirometric data will undergo QC in real time through assessment of the flow-volume loops. A quality grading system for the spirometry values will be determined using the flow volume loops that will be generated when spirometry is performed. The criteria for an acceptable quality will be established and FEV1 value will only be recorded for acceptable quality spirometric manoeuvres (as assessed by the flow-volume loops and consistency of traces obtained). Spirometry will be repeated until acceptable quality flow-volume traces are generated.

The mean FEV1 z-scores (generated using GLI reference standards) will be compared between trial arms 12 months after initiation of azithromycin treatment (primary outcome), adjusted for site and baseline FEV1 z-score.

Other comparisons between the trial arms will include the mean FEV1 z-score at 18 months after initiation of treatment, the number of patients who interrupt a study drug due to adverse events, time to death and to first acute exacerbation. The numbers of acute exacerbations and hospitalisations, and weight gain (measured as change in weight-for-age z-score adjusted for baseline) will be measured at 12 and 18 months (to investigate durability) after treatment initiation. The number of malaria episodes, *Salmonella* blood stream infections and number of gastroenteritis episodes will also be compared between trial arms at 12 months of treatment initiation.

Secondary outcomes at 18 months will be ascertained in those who complete 18 months of follow up (approximately 70 % of participants)

9.5.2 Laboratory sub-studies

Samples for the laboratory sub-studies will be shipped for analysis: microbiology to the University of Cape Town (South Africa), immunology to the University of Oxford (UK) and drug resistance to Malawi-Liverpool-Wellcome Trust Clinical Research Programme (Malawi).

i. Conventional culture will be performed for common bacterial pathogens on baseline, 12 and 18 month sputum and nasopharyngeal samples in trial participants and the proportion of participants colonized with clinically relevant organisms (including *S. aureus, S. pneumoniae, H. influenzae, P. aeruginosa)* in each trial comparison group will be assessed. Antibiotic susceptibility testing will be performed on relevant cultured isolates (including *S. pneumoniae, H. influenzae, S. aureus*) using Vitek-2 (bioMerieux, France) or disk diffusion testing; where relevant penicillin MICs will be determined for *S. pneumoniae* using E-test. The proportion of isolates resistant to macrolides will be compared between trial arms at 12 months to determine the impact of azithromycin therapy on antimicrobial resistance. Pneumococci isolated from NP swabs will undergo further phenotypic and genotypic characterisation, to allow us to define the molecular pathway to macrolide resistance. Total RNA will be extracted from a subset of NP swabs for pneumococcal transcriptomic profile analysis.'

ii. Detailed assessment of the broader bacterial microbial population will be performed on sputum samples and stool samples using Illumina MiSeq (Illumina, Inc. Madison WI) sequencing of bacterial 16S rRNA gene amplicons. The diversity and composition of the bacterial microbiome

will be determined using summary measures of species diversity such as the Shannon Index and Simpson Index, the relative abundances of the dominant species, and the relative abundances of the known pathogenic species.

iii. The proportion of participants colonised with pathogenic organisms, and the microbiome diversity and composition will be compared between:

   a. HIV-infected children with and without CLD to determine the association between CLD and the respiratory/gut microbiome
   b. The two trial arms to assess the effect of azithromycin on the respiratory/gut flora
   c. Participants in the azithromycin group who do and do not show an improvement in lung function to determine whether the respiratory microbiome predicts a response to azithromycin therapy

iv. Plasma levels of high-sensitivity C-reactive protein (CRP), D-dimers, and IL-6 (markers of systemic inflammation), and of beta-2 microglobulin (β2m) and sCD14 (indicators of microbial translocation and immune activation), will be measured using a panel of multiplex bead array assays. Levels of bacterial 16S rRNA in DNA extracted from plasma provide a measure of the extent of microbial translocation and will be quantified using RT-PCR. The levels of these markers will be correlated with FEV1 z-score levels in those with and without CLD, and a comparison of levels between the trial arms will be done.

9.5.3 Cardiac sub-study

The 400 study participants will also undergo echocardiography at enrolment and 12 months of initiation of azithromycin therapy to evaluate changes in pulmonary pressure (if any) and presence of right ventricular dilatation related to study treatment.

9.6 Baseline assessments and follow up visits

9.6.1 Schedule of follow-up and investigations in trial participants

Procedures	Screening	Enrollment	Time post-enrolment							
			0	2 weeks	3 months	6 months	9 months	12 months	15 months	18 months
Demographics and clinical history	✓	✓								
Drug history	✓	✓		✓	✓	✓	✓	✓	✓	✓
Spirometry	✓					✓		✓		✓
Shuttle walk test[a]		✓				✓		✓		✓
Height, weight	✓	✓		✓	✓	✓	✓	✓	✓	✓
ECG,	✓			✓	✓	✓				
Serum creatinine & ALT	✓									
Pregnancy test [b]	✓									
Sputum sample for TB Screening [c]	✓									
Sputum sample[c, d]		✓						✓		✓
Stool sample[d]		✓						✓		✓
Nasopharyngeal swabs[d, e]		✓						✓		✓
Blood sample[d,e]		✓						✓		✓
CD4 count (fingerprick sample)		✓						✓		
HIV viral load[f]		✓						✓		
Cardiac echo[g]		✓						✓		
Supply Trial drug			✓	✓	✓	✓	✓			
Exhaled Nitric Oxide[h]		✓						✓		

[a] Pulse oximetry before, immediately and 1 minute after exercise testing (Shuttle Walk Test[50])

[b] All girls that have reached menarche

[c] Sputum spontaneously expectorated or collected by induction[51]

[d] Sample store for laboratory studies (option of rectal swabs if preferred)

[e] Blood sample for immunology studies (15ml in green top and 1 ml in purple top)

[f] Blood sample for Viral Load (5ml in purple top)

[g] Baseline Echo can be done at enrolment or 2weeks or 3months

[h] Exhaled Nitric Oxide to be done at Harare Site Only

Total blood volumes: 4 ml creatinine and ALT. *Screening*: **4 ml**, *Enrolment*: **15 ml immunology, 5mls HIV Viral load 20ml**, *2 weeks*: **0 ml**, *3 months*: **0 ml**, *6 months*: **0 ml**, *9 months*: **0 ml**, *12 months*: **18 ml**, *15 months:* **0 ml**, *18 months*: **15 ml**

Sample storage: Sputum: STGG and residuals; Stool: store unprocessed; NP swabs: store in STGG and Prime store; Blood: store PBMC, packed red blood cell packet and plasma

Zimbabwe Blood storage: PBMC at baseline and at 12 months in first 100 trial participants only and in 50 participants without CLD. Red blood cell packet stored at baseline in all 200 trial participants and 50 comparison group participants; plasma stored in 50 comparison group participants, and in 200 trial participants at baseline, 12 months and 18 months.

Malawi Blood storage: Store red blood cell packet at baseline in 200 trial participants and in 50 comparison group participants. Plasma stored at baseline, 12 months and 18 months.

Patients will be randomised to either the control regimen or to a weekly weight-based dose of azithromycin (10-19.9 kg: 250 mg; 20-29.9 kg:500mg; 30-39.9 kg:750mg: ≥40 kg:1250mg[52]) for 12 months (30mg/kg/week[53]). Every dose will be given under direct observation (DOT) by a treatment monitor identified within the family.

Assessment procedures at baseline and during the follow-up period are summarised in the table above. Patients will be followed-up 3 monthly up to 6 months after completion of treatment. Visits occurring within 30 days either side of the scheduled visit date will be eligible for inclusion in analysis.

9.6.2 Project plan design

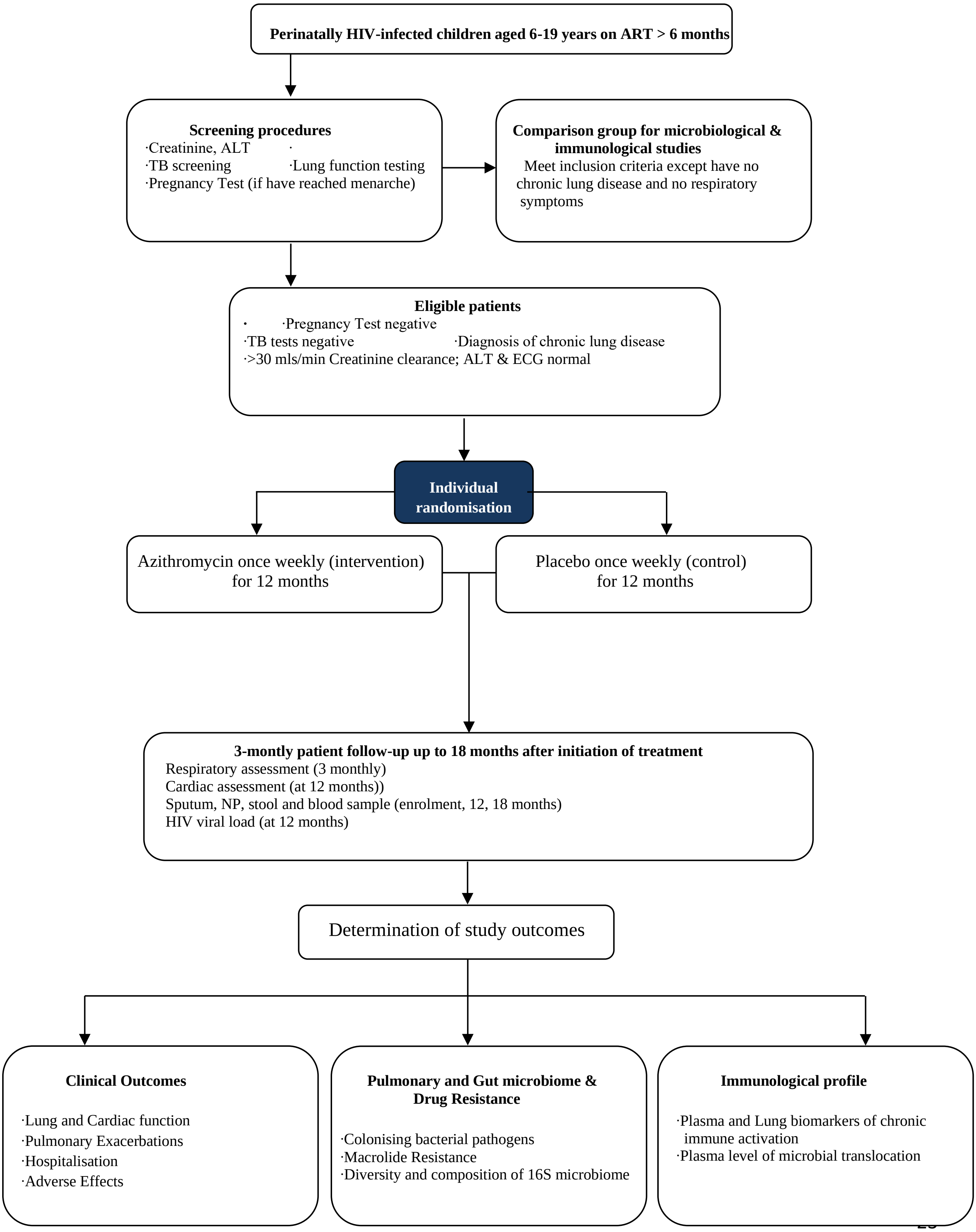

9.6.3 Investigation of acute exacerbations (or infections) and adverse events

Participants will be advised to attend the study clinic outside the scheduled visits if they develop an acute exacerbation (defined as new or worsening respiratory symptoms +/- symptoms and signs of an infection i.e. fever or night sweats with or without any other systemic symptoms). The cause of exacerbation will be result in the following investigations: induced or expectorated sputum and nasopharyngeal swab specimens will be collected. These tests will not be used for clinical decision-making. Management of acute respiratory exacerbations will be with Amoxicillin-Clavulanate (50 mg/kg Amoxicillin) for 10 days. Those with a history of severe allergy to penicillin will be treated with levofloxacin or chloramphenicol (if levofloxacin not available). Those with a minor allergy to penicillin will be treated with oral cefuroxime. In those who do not respond to treatment to standard antimicrobial treatment, chest radiography will be performed (as defined below), and TB investigations carried out: sputum will be examined for mycobacteria through fluorescent microscopy for acid-fast bacilli and liquid culture MGIT. Positive mycobacterial cultures will be identified and speciated as Mycobacterium *tuberculosis* or Mycobacteria other than TB (MOTT). Positive mycobacterial cultures will be stored for more detailed speciation.

Criteria for admission will be: difficulty in breathing and increased oxygen requirements defined as oxygen saturation in room air below 90%.

All febrile episodes will be investigated as follows: blood culture, stool sample and malaria parasites and/or malaria rapid diagnostic test. (Malaria diagnosis for Malawi site and for Harare site only in there is a history of exposure (travel outside Harare). Participants will be treated according to the existing country-specific protocols.

Severe diarrhoeal episodes (defined as DAIDS Grade 3) will be investigated for *Clostridium difficile.* A stool sample will be stored for later aetiologic studies. Participants presenting with jaundice will have an ALT and hepatitis A IgM and Hepatitis B surface antigen (HbsAg) done.

9.6.3 Non-CLD Comparison group

This group of participants will be assessed only at enrolment and not followed up further. Samples will be taken for laboratory sub-studies: sputum, NP swabs and stool/rectal swab for microbiology, and a blood sample for immunology and HIV viral load.

9.7 Discontinuation/Withdrawal of participants from trial treatment

A member of the study team will conduct a home visit for those who miss a study visit. A patient will be considered to have withdrawn from the study if he/she does not attend for a study visit after a home visit or refuses to take the study drug on two or more occasions without there being a reasonable cause. The trial will be terminated early if there is an unacceptable level of adverse events, including development of drug resistance, or in the of an unacceptably high failure rate occurring in both trial arms. In case of a SAR or SUSAR, the study drug will be stopped in all those with Grade 4 events. Individual consideration from the trial management group will be required before considering stopping the study drugs in grade 3 SAR or SUSAR.

9.8 Definition of end of trial

The trial will be considered closed 12 months after the last patient is enrolled and all follow-up and laboratory reports have been received. Early termination could occur if there is an unacceptable level of adverse events in the Azithromycin arm or evidence of efficacy without doubt.

10 Investigational Medicinal Product

10.1 IMP description

10.1.1 Azithromycin

Azithromycin monohydrate 250 mg tablet film coated.
Manufacturer: Teva Pharmaceuticals, Pennsylvania, USA
Manufacturing site: USA
Packaging: bulk/blister.

10.1.2 Placebo

Placebo for Azithromycin 250 mg tablets film coated,
Manufacturer: Piramal Pharmaceutical Development Services Pvt. Ltd
Manufacturing site: India
Packaging: bulk/blister.

10.2 Storage IMP

Storage requirements for IMP and placebo are ambient temperature (<25ºC). Both will be stored in approved research pharmacies and administered by a pharmacist of record. Once the centre has received approval for participation from their appropriate regulatory body, arrangements will be made for the despatch of the drugs to the designated pharmacy. Each centre will be responsible for the clearance of the drugs from their Customs Department.

10.3 Compliance of the trial treatment

Adherence to treatment will be measured by counting tablets remaining in each visit and maintenance of a DOTS diary by a designated caregiver. A pill count and check of the DOTS diary will be used to monitor adherence to treatment. The DOTS diary will be collected from all participants at completion of treatment (at 12 months).

10.4 Accountability of trial treatment

Drug stocks will be regularly monitored and the remaining stocks checked against the amounts dispensed. Once all study drug has been accounted for, the remaining drug product will be destroyed by a contracted third party certified to do so. The destruction will be accounted for in writing, and the drug product, quantity, batch numbers and date of destruction recorded. A dated certificate of drug destruction will be provided to the sponsor.

10.5 Dispensing

Study staff has the responsibility to ensure that study drugs are dispensed and administered to patients in compliance with the protocol. Procedures consistent with ICH GCP guidelines for recording of drug disposition, drug handling and drug storage will be described in the study site SOPs.

10.6 Concomitant medication

ART and cotrimoxazole will be continued during the course of the study. Drug history will be recorded at enrollment and all follow up visits. Any new drugs commenced during the course of the study will be discussed with the study physician to exclude the possibility of drug interactions, specifically prolongation of the QTc interval.

10.7 Post-trial treatment

All participants will continue normal follow ups in their designated ART clinics, their HIV care being the responsibility of these clinics.

11 Safety Reporting

11.1 Definitions

The definitions of the EU Directive 2001/20/EC Article 2 based on the principles of ICH GCP apply to this trial protocol.

Table	Definition
Adverse Event (AE)	Any untoward medical occurrence in a patient or clinical trial subject to whom a medicinal product has been administered including occurrences that are not necessarily caused by or related to that product.
Adverse Reaction (AR)	Any untoward and unintended response to an investigational medicinal product related to any dose administered.
Unexpected Adverse Reaction (UAR)	An adverse reaction, the nature or severity of which is not consistent with the information about the medicinal product in question set out in the Summary of Product Characteristics (SPC) or Investigator Brochure (IB) for that product.
Serious Adverse Event (SAE) or Serious Adverse Reaction (SAR) or Suspected Unexpected Serious Adverse Reaction (SUSAR)	Respectively any adverse event, adverse reaction or unexpected adverse reaction that:<br>• Results in death<br>• Is life-threatening[a]<br>• Requires hospitalisation or prolongation of existing hospitalisation[b]<br>• Results in persistent or significant disability or incapacity<br>• Consists of a congenital anomaly or birth defect<br>• Is another important medical condition[c]

[a] The term life-threatening in the definition of a serious event refers to an event in which the patient is at risk of death at the time of the event; it does not refer to an event that hypothetically might cause death if it were more severe, for example, a silent myocardial infarction.

[b] Hospitalisation is defined as an inpatient admission, regardless of length of stay, even if the hospitalisation is a precautionary measure for continued observation. Hospitalisations for a pre-existing condition (including elective procedures that have not worsened) do not constitute an SAE.

[c] Medical judgement should be exercised in deciding whether an AE or AR is serious in other situations. The following should also be considered serious: important AEs or ARs that are not immediately life-threatening or do not result in death or hospitalisation but may jeopardise the subject or may require intervention to prevent one of the other outcomes listed in the definition above; for example, a secondary malignancy, an allergic bronchospasm requiring intensive emergency treatment, seizures or blood dyscrasias that do not result in hospitalisation or development of drug dependency.

Severity will be according to the modified DAIDS Classification (19.3)

11.1.1 Known important/frequent Azithromycin side effects

	DAIDS grading			
	**1 MILD**	**2 MODERATE**	**3 SEVERE**	**4 POTENTIALLY LIFE-THREATENING**
	Symptoms causing no or minimal interference with usual social & functional activities	Symptoms causing greater than minimal interference with usual social & functional activities	Symptoms causing inability to perform usual social & functional activities	Symptoms causing inability to perform basic self-care functions OR Medical or operative intervention indicated to prevent permanent impairment, persistent disability, or death
**Side-effects**	**1**	**2**	**3**	**4**
**Acute Allergic Reaction**	Localized urticaria (wheals) with no medical intervention indicated	Localized urticaria with intervention indicated OR Mild angioedema with no intervention indicated	Generalized urticaria OR Angioedema with intervention indicated OR Symptoms of mild bronchospasm	Acute anaphylaxis OR Life-threatening bronchospasm OR Laryngeal edema
**Rash** ***Specify type, if applicable***	Localized rash	Diffuse rash OR Target lesions	Diffuse rash AND Vesicles or limited number of bullae or superficial ulcerations of mucous membrane limited to one site	Extensive or generalized bullous lesions OR Ulceration of mucous membrane involving two or more distinct mucosal sites OR Stevens-Johnson syndrome OR Toxic epidermal necrolysis
**Side-effects**	**1**	**2**	**3**	**4**
**Arrhythmia (by ECG or physical examination)** ***Specify type, if applicable***	No symptoms AND No intervention indicated	No symptoms AND Non-urgent intervention indicated	Non-life-threatening symptoms AND Non-urgent intervention indicated	Life-threatening arrhythmia OR Urgent intervention indicated
**Prolonged QTc Interval**	0.45 to 0.47 seconds	> 0.47 to 0.50 seconds	> 0.50 seconds OR ≥ 0.06 seconds above baseline	Life-threatening consequences (e.g., Torsade de pointes, other associated serious ventricular dysrhythmia)

	DAIDS grading			
	1 MILD	2 MODERATE	3 SEVERE	4 POTENTIALLY LIFE-THREATENING
Side-effects *Cont.*	1	2	3	4
**Diarrhea ≥ *1 year of age***	Transient or intermittent episodes of unformed stools OR Increase of ≤ 3 stools over baseline per 24-hour period	Persistent episodes of unformed to watery stools OR Increase of 4 to 6 stools over baseline per 24-hour period	Increase of ≥ 7 stools per 24-hour period OR IV fluid replacement indicated	Life-threatening consequences (e.g., hypotensive shock)
**Tinnitus**	Symptoms causing no or minimal interference with usual social & functional activities with intervention not indicated	Symptoms causing greater than minimal interference with usual social & functional activities with intervention indicated	Symptoms causing inability to perform usual social & functional activities	NA
**Nausea**	Transient (< 24 hours) or intermittent AND No or minimal interference with oral intake	Persistent nausea resulting in decreased oral intake for 24 to 48 hours	Persistent nausea resulting in minimal oral intake for > 48 hours OR Rehydration indicated (e.g., IV fluids)	Life-threatening consequences (e.g., hypotensive shock)
**Vomiting**	Transient or intermittent AND No or minimal interference with oral intake	Frequent episodes with no or mild dehydration	Persistent vomiting resulting in orthostatic hypotension OR Aggressive rehydration indicated (e.g., IV fluids)	Life-threatening consequences (e.g., hypotensive shock)
**Laboratory**	**1**	**2**	**3**	**4**
**ALT or SGPT, High** ***Report only one***	1.25 to < 2.5 x ULN	2.5 to < 5.0 x ULN	5.0 to < 10.0 x ULN	≥ 10.0 x ULN
**Creatinine Clearance or eGFR, Low** ***Report only one***	NA	< 90 to 60 ml/min or ml/min/1.73 m2 OR 10 to < 30% decrease from baseline	< 60 to 30 ml/min or ml/min/1.73 m2 OR ≥ 30 to < 50% decrease from baseline	< 30 ml/min or ml/min/1.73 m2 OR ≥ 50% decrease from baseline or dialysis needed

11.2 Causality

When reporting on serious adverse events, the trial investigator will state whether they believe that the event is causally associated with any of the trial treatments and the strength of the causal relationship. They will also state whether the adverse event was expected and what if any action was taken.

Relationship	Description	SAE Type
Unrelated	There is no evidence of any causal relationship	Unrelated SAE
Unlikely	There is little evidence to suggest that there is a causal relationship (for example, the event did not occur within a reasonable time after administration of the trial medication). There is another reasonable explanation for the event (for example, the patient's clinical condition, other concomitant treatment).	Unrelated SAE
Possible	There is some evidence to suggest a causal relationship (for example, because the event occurs within a reasonable time after administration of the trial medication). However, the influence of other factors may have contributed to the event (for example, the patient's clinical condition, other concomitant treatments).	SAR
Probable	There is evidence to suggest a causal relationship and the influence of other factors is unlikely.	SAR
Definitely	There is clear evidence to suggest a causal relationship and other possible contributing factors can be ruled out.	SAR

11.3 Procedures for recording adverse events

- Patients will be closely monitored for signs and symptoms of drug toxicity and will be asked about side effects at each study visit.
- All patients will have repeat ECG at 2 weeks, 3 and 6 months follow-up visit and an ALT done if they present with jaundice. C-difficile test will be done if participants present with DAIDS 3 diarrhoea
- The study drug will be discontinued if an AR is grade 4 and considered in grade 3 ARs events, but follow-up will continue as per protocol.
- In case of reported death in the community, the caregiver will be followed up and questioned to elucidate the cause of death.

11.4 Procedures for reporting serious adverse events

All serious adverse events (SAEs) and Grade 3 and 4 ARs (including those suspected), will be recorded on the relevant regulatory reporting forms and reported immediately to the site Principal Investigator who is responsible for ensuring the reporting of the event (within 2 working days) to the relevant ethics committees, regulatory authorities, the DSMB and the sponsor.

11.5 Safety Monitoring Committee

A DSMB will be constituted to provide independent review of the study conduct, progress and findings. The proposed DSMB will comprise 3 members including a chairperson who will be responsible for collating and communicating the views of the DSMB. The DSMB will consist of an independent statistician and two clinicians, at least one of them a paediatrician, with research experience and expertise in the management of HIV.

The proposed data safety monitoring plan will be discussed in a teleconference including the DSMB members and the key investigators prior to the study starting. The proposed schedule will include annual reports to be presented to the DSMB. The reports will review study approvals, progress and obstacles, and include statistics of enrolment, refusals to participate/non-enrolment, adverse events, withdrawals and trial efficacy measures, by site. The DSMB will be requested to meet via teleconference within 1 month of receiving these regular reports and to provide written feedback to the principal investigator.

11.6 Pregnancy

Azithromycin is a FDA category B drug, so animal studies have failed to demonstrate a negative effect on pregnancy and fetus. Still pregnant women will be withdrawn from the study mainly due to the effect on lung function testing.

If a participant becomes pregnant during the course of the study the study drug will be stopped and treatment allocation unblinded and referred for antenatal care regardless of treatment arm. The participant will continue follow up, to monitor pregnancy. The infant shall be referred to a paediatrician for assessment at birth and a 6 week visit.

12 Statistical Considerations

12.1 Sample size justification

Our original sample size calculations were based on estimates obtained from 30 participants (with FEV1z<-1.64) enrolled in a study conducted in Zimbabwe of HIV-infected children of a similar age-group treated with ART, with mean -2.44 and SD 0.536.

We have revised our sample size calculations using records of those screened in the BREATHE trial to Jan 2017 for two reasons; 1) to establish the standard deviation of FEV1z scores in the screened population (which include 368 screened) and 2) to revisit the estimates of the mean and standard deviation using the revised eligibility cut-point of FEV1z<-1.

Using baseline FEV1 z-scores from 136 subjects with FEV1z<-1, the mean was estimated to be -2.04 and SD 0.82. We have revised our sample size calculations to present a range of scenarios for the standard deviation in the figure below.

The following assumptions were used:

1. Mean FEV1-zscore of -2.04 among control group
2. Patients randomised in equal proportions to the two regimens
3. Up to 25% of participants un-assessable due loss to follow-up or death or suboptimal spirometric traces
4. Conservative assumption of no change in FEV1-z over the study period in the control arm (lung function anticipated to decline over time without treatment)
5. Difference in trial arm mean FEV1 z-scores ranging from 0.15 to 0.3, an effect assumed to be of clinical relevance
6. SD ranging from 0.55 to 0.82 to assess the impact of variability on the difference in mean we have power to detect

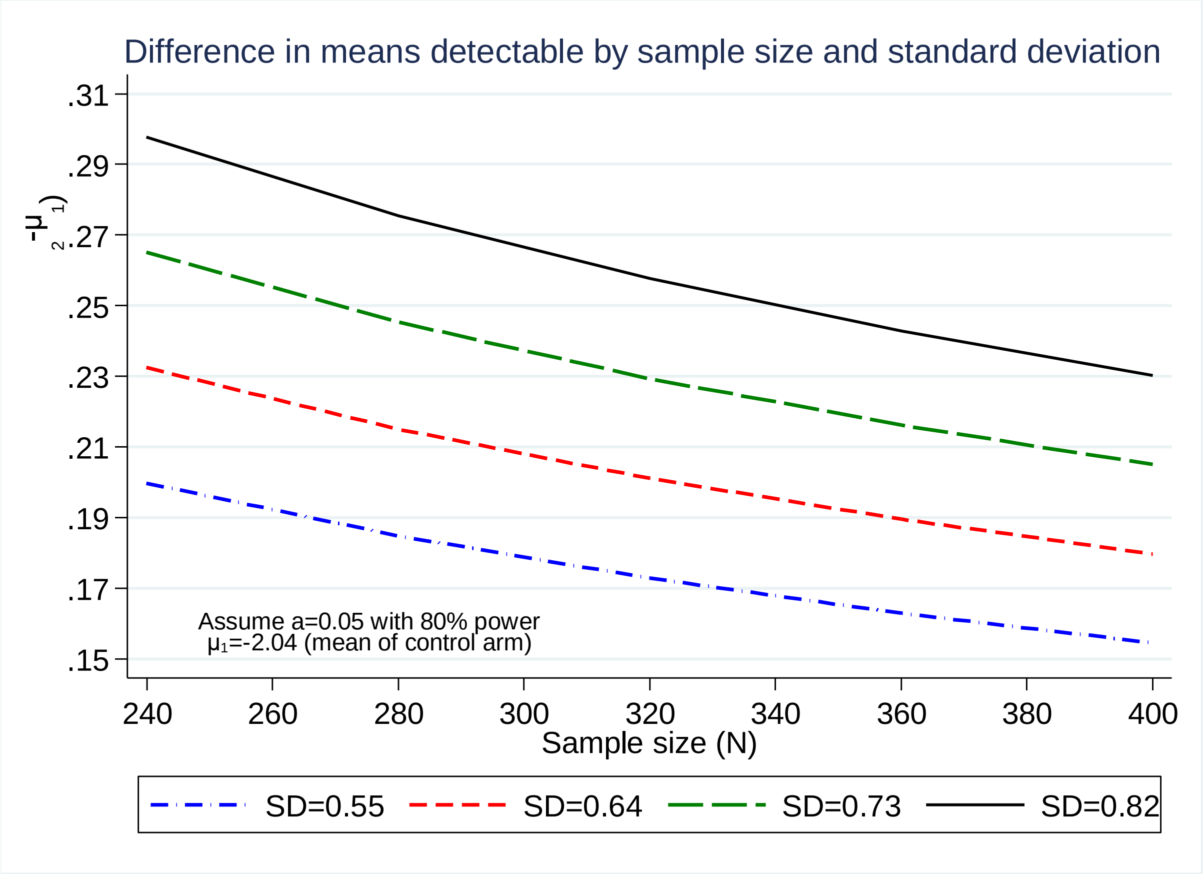

Therefore, a sample size of 400 recruited participants and 300 participants with outcome data (25% unassessable) will enable 80% power to detect a difference in trial arm means ranging between 0.18 and 0.27, an effect size (difference in means/SD) of 0.32.

To recruit the required sample size of 400 children with CLD and no active TB an estimated 2000 children will be screened at each site. This assumes that 15% will have chronic lung disease without active TB[54] and 30% will be excluded for different reasons (active TB, refusals or spirometry tracings of sub-optimal quality).

12.2 Data analysis plan

The primary analysis will be modified intention-to-treat. Secondary analyses will include a per protocol analysis. The mean FEV1 z-score at 12 months (primary outcome) and at 18 months after treatment will be compared between treatment groups, adjusting for site and baseline FEV1 z-score, using linear regression to estimate the mean difference and corresponding 95% confidence interval (CI). Time-to-first exacerbation and time-to-death will be assessed using Cox proportional hazards regression and graphically displayed using Kaplan-Meier estimates. Between-group comparisons of binary outcomes (e.g. for hypoxia and colonization with drug resistant organisms) will be analysed with logistic regression, to estimate odds ratios and 95% CI. Count data (e.g. number of hospitalisations) will be analysed using Poisson regression. All analyses will adjust for site. Continuous outcomes will be additionally adjusted for the baseline measure of the outcome. Missing outcome data will be imputed using multiple imputation in Stata, according to plausible assumptions about missingness, if appropriate. Pre-specified effect modification analyses will include site and baseline severity of lung disease.

12.3 Microbiome and Immunology analysis

The effect of the intervention on the respiratory and gut microbiome will be assessed using weighted generalized ridge regression models. A model will be estimated at each time point where samples are available. This approach is able to efficiently select variables in a high dimensional setting with high collinearity as is the case in microbiome profiles. Logistic regression modelling will be used to develop a predictive model of response to azithromycin therapy based on microbiome profile elements as well as important covariates such as age and repeated infection. We will leverage cross-validation approaches to identifying robust predictive models and assess their performance as predictors using receiver-operator characteristic (ROC) curve analyses. Median levels of biomarkers will be calculated and compared between arms using a Mann-Whitney U test. The association between biomarker levels and FEV1 z-scores will be investigated using linear regression. A similar approach will be employed to determine the impact of treatment on pulmonary macrophage phagocytic function.

13 Data Management

13.1 Source Data

Source documents are where data are first recorded, and from which participants' CRF data are obtained. These include, but are not limited to, hospital records (from which medical history and previous and concurrent medication may be summarised into the CRF), clinical and office charts, laboratory and pharmacy records, diaries, radiographs, and correspondence.

CRF entries will be considered source data if the CRF is the site of the original recording (e.g. there is no earlier written or electronic record of data). All documents will be stored safely in confidential conditions and held for 15 years after the end of the trial in accordance with GCP principles. On all trial-specific documents, other than the signed consent forms household locator form and, the participant will be referred to by the trial participant number/code, not by name. The consent forms

and locator forms will be kept separate from the CRFs to avoid linkage between participant name and the study ID.

13.2 Access to data

Direct access will be granted to authorised representatives from the Sponsor, host institution and the regulatory authorities to permit trial-related monitoring, audits and inspections.

13.3 Data recording and record keeping

In Malawi TELEFORM (an optical recognition system for data entry) will be used for data management. Appropriate CRFs will be designed for each type of data to be collected. Where possible, categorical responses will be converted into numerical codes on the CRF. Completed CRFs will be scanned using TELEFORM. In Zimbabwe clinical forms will be managed using electronic data management with OPTIFORM or ODK eCRFs loaded onto tablets and incorporating predefined consistency checks. The laboratory forms will be managed using the TELEFORM system. In both countries data will be extracted, processed and saved to a Microsoft Access database, before being exported to Stata and merged into a single database for analysis. To enable more efficient data management the CRFS will be developed from a common template in both countries and use equivalent checks. Spirometry data will be downloaded directly from the spirometer and z-scores will be calculated by referral to a standard population.

The participants will be identified by a unique trial specific number and/or code in any database. The name and any other identifying detail will be removed from all data files before analysis.

13.4 Data cleaning and validation

Quality control will be applied at each stage of data handling in accordance with GCP requirements to ensure that all data are reliable and have been processed correctly. Quality control measures will include

1. Manual checking of all CRFs for completeness, accuracy and legibility before scanning
2. Automatic pre-programmed validation checks after scanning
3. A security system that prevents unauthorized access to the data
4. Regular backup of the data
5. Documentation of all data queries and corrections

14 Trial Monitoring

A Trial Steering Committee (TSC) will oversee trial progress, regularly review relevant information from other sources (e.g. other related trials) and consider recommendations of the DSMB. The DSMB will advise the TSC Chair if, in its view, the findings provide:

a) Evidence based on a-priori criteria (defined by the DSMB) that azithromycin is clearly indicated or clearly contraindicated in terms of efficacy, and any benefit outweighs any serious adverse effects of treatment or vice versa, and
b) Evidence that might reasonably be expected to influence the patient management of many clinicians who are already aware of the results of any other studies.

Internal monitoring will be conducted in both sites according to study specific SOP.

15 Ethical Considerations and Approval

The Investigator will ensure that this trial is conducted in accordance with the principles of the Declaration of Helsinki and in full conformity with relevant regulations and with the ICH Guidelines for Good Clinical Practice (CPMP/ICH/135/95) July 1996.

The protocol, informed consent form, participant information sheet and any proposed advertising material will be submitted to an appropriate Research Ethics Committee: COMREC (Malawi), Medical Research Council of Zimbabwe and BRTI Ethics Committee (Zimbabwe), LSHTM Ethics Committee (UK) and the University of Tromso Ethics Committee (Norway), regulatory authorities, and host institution(s) for written approval.

The Investigator will submit and, where necessary, obtain approval from the above parties for all substantial amendments to the original approved documents.

Risks and Benefits*:* Participants will benefit from optimised management of infections and there are minimal risks associated with study procedures.[55] Expenses related to transport for study visits will be reimbursed.

Confidentiality: The data team will sign a confidentiality agreement prohibiting disclosure of any patient-level information. The study databases will be password protected, and all electronic communications involving study data will be encrypted. The patients' data and specimens will be identified by trial number only.

Informed Consent*:* Written informed consent in the local vernacular will be sought from guardians of all patients with written assent from participants. Consent/assent will be sought for conducting screening procedures, with separate consent/assent to participate in the trial, including administration of the study drug, study and home visits and study tests.

Disclosure of HIV Status*:* Counselling will be provided to support age-appropriate disclosure to participants. Study procedures will ensure that inadvertent disclosure does not occur.

Ethical approval*:* Ethical approval for the study protocol will be sought from the Medical Ethics Committee of each participating clinical site as well as from the London School of Hygiene and Tropical Medicine, UK and from the University of Tromso, Norway.

16 Regulatory approval

Principal Investigators will be expected to obtain, in writing, approval to participate from their Regulatory Authority.

17 Inspection and Audits

The study may be subject to audit by the London School of Hygiene and Tropical Medicine under their remit as Sponsor, the Study Coordination Centre and other regulatory bodies to ensure adherence to GCP.

18 Finance and Insurance

18.1 Funding

This trial is funded by the Medical Research Council of Norway.
Each participating centre will be supported according to the submissions of their budgetary requirements.
Reimbursements will be made according to a Contract/Memorandum of Understanding (MOU) signed between the sponsor and the participating centres.

18.2 Insurance

London School of Hygiene & Tropical Medicine holds Public Liability ("negligent harm") and Clinical Trial ("non-negligent harm") insurance policies which apply to this trial. Patients will be indemnified, independent of proof of fault, through a separate policy taken out by the trial sponsor and fronted in the specific sites according to Regulatory Authority regulations.
All personnel involved in the trial will be expected to be indemnified by their employing authority.

19 Publication

Data will be primarily shared with scientific collaborators, but we will as far as possible facilitate data sharing with any group requesting access to individual patient records, using anonymised data. Where appropriate, and with the proper safeguards, data will be made freely available through the partners (Malawi-Liverpool Wellcome Trust (MLW) Programme website).

Beside the original data, anonymised databases will be created and stored with all relevant data to facilitate any data transfer requests that are made subsequently. Ethical clearance will be sought before data are transferred to other groups for secondary analysis. Priority will be given to local investigators and publicly funded international investigators with data management and sharing policies in line with those of the MLW programme, the College of Medicine and the MRCZ. The governance of data and materials generated by the programme, and the monitoring of their use either locally or abroad, is an area that is currently under detailed review by ourselves and our partners at

the University of Malawi, College of Medicine. The program plans to appoint a research governance officer, whose remit will include the oversight of this process.

We will engage our partners in the respective Ministries of Health at the trial sites before starting our research and will report our findings to them regularly.

Results will be disseminated to communities through our Community Advisory Groups, presented at national and international meetings, and published in journals in compliance with the Open Access policy of the CoM.

Established and developing policies on community engagement and communication will also assist us in disseminating our research findings. Public engagement within Malawi and Zimbabwe would mainly be through a) the national strategy and b) community advisory groups. There has been an increasing emphasis on communication with study populations and the general public. The aim is to improve the public understanding of our work and purpose, and to better facilitate the flow of ideas from the public to researchers and back again. Focus will be on the communities where the researchers work; developing, evaluating, and implementing communication tools in these different environments. Community Advisory Groups (CAGs) will be established at both sites and these will promote better understanding of the research by local communities and a more comprehensive understanding by the researchers of community ideas and perceptions. In this manner a stronger conduit to facilitate the bidirectional flow of information between communities and researchers will be built.

20 References

1. Ferrand RA, Bandason T, Musvaire P, et al. Causes of acute hospitalization in adolescence: burden and spectrum of HIV-related morbidity in a country with an early-onset and severe HIV epidemic: a prospective survey. PLoS Med 2010;7:e1000178.
2. Lucas SB, Peacock CS, Hounnou A, et al. Disease in children infected with HIV in Abidjan, Cote d'Ivoire. BMJ 1996;312:335-8.
3. Norton KI, Kattan M, Rao JS, et al. Chronic radiographic lung changes in children with vertically transmitted HIV-1 infection. AJR AmJRoentgenol 2001;176:1553-8.
4. Jeena PM, Coovadia HM, Thula SA, Blythe D, Buckels NJ, Chetty R. Persistent and chronic lung disease in HIV-1 infected and uninfected African children. Aids 1998;12:1185-93.
5. Zar HJ. Chronic lung disease in human immunodeficiency virus (HIV) infected children. Pediatr Pulmonol 2008;43:1-10.
6. Gona P, Van Dyke RB, Williams PL, et al. Incidence of opportunistic and other infections in HIV-infected children in the HAART era. Jama 2006;296:292-300.
7. Ferrand RA, Desai SR, Hopkins C, et al. Chronic lung disease in adolescents with delayed diagnosis of vertically acquired HIV infection. Clin Infect Dis 2012;55:145-52.
8. Rylance J, Mwalukomo T, Rylance S, et al. Lung function and bronchodilator response in perinatally HIV-infected Malawian adolescents. 19th Conference on Retroviruses and Opportunistic Infections; 2012; Seattle, WA.
9. Hansell DM, Rubens MB, Padley SP, Wells AU. Obliterative bronchiolitis: individual CT signs of small airways disease and functional correlation. Radiology 1997;203:721-6.
10. Lynch JP, 3rd, Weigt SS, DerHovanessian A, Fishbein MC, Gutierrez A, Belperio JA. Obliterative (constrictive) bronchiolitis. Semin Respir Crit Care Med 2012;33:509-32.
11. Colom AJ, Teper AM, Vollmer WM, Diette GB. Risk factors for the development of bronchiolitis obliterans in children with bronchiolitis. Thorax 2006;61:503-6.
12. Sato M, Keshavjee S. Bronchiolitis obliterans syndrome: alloimmune-dependent and -independent injury with aberrant tissue remodeling. Semin Thorac Cardiovasc Surg 2008;20:173-82.
13. Grossman Z, Meier-Schellersheim M, Sousa AE, Victorino RM, Paul WE. CD4+ T-cell depletion in HIV infection: are we closer to understanding the cause? Nat Med 2002;8:319-23.
14. Brenchley JM, Price DA, Schacker TW, et al. Microbial translocation is a cause of systemic immune activation in chronic HIV infection. Nat Med 2006;12:1365-71.
15. Kelesidis T, Kendall MA, Yang OO, Hodis HN, Currier JS. Biomarkers of microbial translocation and macrophage activation: association with progression of subclinical atherosclerosis in HIV-1 infection. J Infect Dis 2012;206:1558-67.
16. Verleden GM, Vanaudenaerde BM, Dupont LJ, Van Raemdonck DE. Azithromycin reduces airway neutrophilia and interleukin-8 in patients with bronchiolitis obliterans syndrome. Am J Respir Crit Care Med 2006;174:566-70.
17. Hilty M, Burke C, Pedro H, et al. Disordered microbial communities in asthmatic airways. PLoS ONE 2010;5:e8578.
18. Sze MA, Dimitriu PA, Hayashi S, et al. The lung tissue microbiome in chronic obstructive pulmonary disease. Am J Respir Crit Care Med 2012;185:1073-80.
19. Charlson ES, Diamond JM, Bittinger K, et al. Lung-enriched organisms and aberrant bacterial and fungal respiratory microbiota after lung transplant. Am J Respir Crit Care Med 2012;186:536-45.
20. Willner DL, Hugenholtz P, Yerkovich ST, et al. Reestablishment of recipient-associated microbiota in the lung allograft is linked to reduced risk of bronchiolitis obliterans syndrome. Am J Respir Crit Care Med 2013;187:640-7.
21. Gollwitzer ES, Marsland BJ. Microbiota abnormalities in inflammatory airway diseases - potential for therapy. Pharmacol Ther 2013.
22. Morris A, Beck JM, Schloss PD, et al. Comparison of the respiratory microbiome in healthy nonsmokers and smokers. Am J Respir Crit Care Med 2013;187:1067-75.

23. Lozupone C, Cota-Gomez A, Palmer BE, et al. Widespread colonization of the lung by Tropheryma whipplei in HIV infection. Am J Respir Crit Care Med 2013;187:1110-7.
24. Altenburg J, de Graaff CS, Stienstra Y, et al. Effect of azithromycin maintenance treatment on infectious exacerbations among patients with non-cystic fibrosis bronchiectasis: the BAT randomized controlled trial. Jama 2013;309:1251-9.
25. Wong C, Jayaram L, Karalus N, et al. Azithromycin for prevention of exacerbations in non-cystic fibrosis bronchiectasis (EMBRACE): a randomised, double-blind, placebo-controlled trial. Lancet 2012;380:660-7.
26. Serisier DJ, Martin ML, McGuckin MA, et al. Effect of long-term, low-dose erythromycin on pulmonary exacerbations among patients with non-cystic fibrosis bronchiectasis: the BLESS randomized controlled trial. Jama 2013;309:1260-7.
27. Albert RK, Connett J, Bailey WC, et al. Azithromycin for prevention of exacerbations of COPD. N Engl J Med 2011;365:689-98.
28. Southern KW, Barker PM, Solis-Moya A, Patel L. Macrolide antibiotics for cystic fibrosis. Cochrane Database Syst Rev 2012;11:CD002203.
29. Khalid M, Al Saghir A, Saleemi S, et al. Azithromycin in bronchiolitis obliterans complicating bone marrow transplantation: a preliminary study. Eur Respir J 2005;25:490-3.
30. Verleden GM, Dupont LJ. Azithromycin therapy for patients with bronchiolitis obliterans syndrome after lung transplantation. Transplantation 2004;77:1465-7.
31. Lee J, Cha SI, Park TI, Park JY, Jung TH, Kim CH. Adjunctive effects of cyclosporine and macrolide in rapidly progressive cryptogenic organizing pneumonia with no prompt response to steroid. Intern Med 2011;50:475-9.
32. Amsden GW, Gray CL. Serum and WBC pharmacokinetics of 1500 mg of azithromycin when given either as a single dose or over a 3 day period in healthy volunteers. J Antimicrob Chemother 2001;47:61-6.
33. Wildfeuer A, Laufen H, Zimmermann T. Distribution of orally administered azithromycin in various blood compartments. Int J Clin Pharmacol Ther 1994;32:356-60.
34. Scaglione F, Rossoni G. Comparative anti-inflammatory effects of roxithromycin, azithromycin and clarithromycin. J Antimicrob Chemother 1998;41 Suppl B:47-50.
35. Jain R, Hachem RR, Morrell MR, et al. Azithromycin is associated with increased survival in lung transplant recipients with bronchiolitis obliterans syndrome. J Heart Lung Transplant 2010;29:531-7.
36. Gottlieb J, Szangolies J, Koehnlein T, Golpon H, Simon A, Welte T. Long-term azithromycin for bronchiolitis obliterans syndrome after lung transplantation. Transplantation 2008;85:36-41.
37. Uthman MM, Uthman OA, Yahaya I. Interventions for the prevention of mycobacterium avium complex in adults and children with HIV. Cochrane Database Syst Rev 2013;4:CD007191.
38. Keenan JD, Ayele B, Gebre T, et al. Childhood mortality in a cohort treated with mass azithromycin for trachoma. Clin Infect Dis 2011;52:883-8.
39. Porco TC, Gebre T, Ayele B, et al. Effect of mass distribution of azithromycin for trachoma control on overall mortality in Ethiopian children: a randomized trial. Jama 2009;302:962-8.
40. Saiman L, Marshall BC, Mayer-Hamblett N, et al. Azithromycin in patients with cystic fibrosis chronically infected with Pseudomonas aeruginosa: a randomized controlled trial. Jama 2003;290:1749-56.
41. Saiman L, Anstead M, Mayer-Hamblett N, et al. Effect of azithromycin on pulmonary function in patients with cystic fibrosis uninfected with Pseudomonas aeruginosa: a randomized controlled trial. Jama 2010;303:1707-15.
42. Ray WA, Murray KT, Hall K, Arbogast PG, Stein CM. Azithromycin and the risk of cardiovascular death. N Engl J Med 2012;366:1881-90.
43. Skalet AH, Cevallos V, Ayele B, et al. Antibiotic selection pressure and macrolide resistance in nasopharyngeal Streptococcus pneumoniae: a cluster-randomized clinical trial. PLoS Med 2010;7:e1000377.

44. UNAIDS. Global HIV/AIDS response: Epidemic update and health sector progress towards Universal Access. Geneva, Switzerland: UNAIDS and WHO; 2011.
45. Global update on HIV treatment 2013: Results, impact and opportunities. Geneva, Switzerland: World Health Organization; 2013.
46. Ferrand R, Lowe S, Whande B, et al. Survey of children accessing HIV services in a high prevalence setting: time for adolescents to count? Bull World Health Organ 2010;88:428-34.
47. Fatti G, Bock P, Eley B, Mothibi E, Grimwood A. Temporal trends in baseline characteristics and treatment outcomes of children starting antiretroviral treatment: an analysis in four provinces in South Africa, 2004-2009. J Acquir Immune Defic Syndr 2011;58:e60-7.
48. van Dijk JH, Sutcliffe CG, Munsanje B, et al. HIV-infected children in rural Zambia achieve good immunologic and virologic outcomes two years after initiating antiretroviral therapy. PLoS One 2011;6:e19006.
49. Mwalukomo T, Rylance SJ, Webb EL, et al. Clinical Characteristics and Lung Function in Older Children Vertically Infected With Human Immunodeficiency Virus in Malawi. Journal of the Pediatric Infectious Diseases Society 2015.
50. Bradley J, Howard J, Wallace E, Elborn S. Validity of a modified shuttle test in adult cystic fibrosis. Thorax 1999;54:437-9.
51. Wong HH, Fahy JV. Safety of one method of sputum induction in asthmatic subjects. Am J Respir Crit Care Med 1997;156:299-303.
52. Steinkamp G, Schmitt-Grohe S, Doring G, et al. Once-weekly azithromycin in cystic fibrosis with chronic Pseudomonas aeruginosa infection. Respir Med 2008;102:1643-53.
53. Wilms EB, Touw DJ, Heijerman HG, van der Ent CK. Azithromycin maintenance therapy in patients with cystic fibrosis: a dose advice based on a review of pharmacokinetics, efficacy, and side effects. Pediatr Pulmonol 2012;47:658-65.
54. Nglazi MD, Kranzer K, Holele P, et al. Treatment outcomes in HIV-infected adolescents attending a community-based antiretroviral therapy clinic in South Africa. BMC Infect Dis 2012;12:21.
55. Zar HJ, Tannenbaum E, Hanslo D, Hussey G. Sputum induction as a diagnostic tool for community-acquired pneumonia in infants and young children from a high HIV prevalence area. Pediatr Pulmonol 2003;36:58-62.

21 Appendices

21.1 Toxicity Grading (DAIDS)

Division of AIDS table for grading the severity of adverse events version 2.0 (published in November 2014)
ULN = Upper Limit of Normal ; LLN = Lower Limit of Normal

General Instructions:
If the need arises to grade a clinical adverse event (AE) that is not identified in the DAIDS AE grading table, us the category "Estimating Severity Grade" located at the top of the table.

If the severity of an AE could fall under either one of two grades (e.g. the severity of an AE could be either Grade 2 or Grade 3) select the higher of the two grades for the AE.

Definitions:
Basic Self-care Functions Adult: Activities such as bathing, dressing, toileting, transfer/movement, continence, and feeding.

Usual Social & Functional Activities Adult: Adaptive tasks and desirable activities, such as going to work, shopping, cooking, use of transportation, pursuing a hobby etc.

Estimating Severity grade for parameters not identified in the grading table: The functional table below should be used to grade the severity of an AE that is not specifically identified in the grading table. In addition, all deaths related to an AE are to be classified as grade 5.

PARAMETER	GRADE 1 MILD	GRADE 2 MODERATE	GRADE 3 SEVERE	GRADE 4 POTENTIALLY LIFE-THREATENING
**Clinical adverse event NOT identified elsewhere in the grading table**	Mild symptoms causing no or minimal interference with usual social & functional activities with intervention not indicated	Moderate symptoms causing greater than minimal interference with usual social & functional activities with intervention indicated	Severe symptoms causing inability to perform usual social & functional activities with intervention or hospitalization indicated	Potentially life-threatening symptoms causing inability to perform basic self-care functions with intervention indicated to prevent permanent impairment, persistent disability, or death

1. *Major clinical conditions:*

CARDIOVASCULAR

PARAMETER	GRADE 1 MILD	GRADE 2 MODERATE	GRADE 3 SEVERE	GRADE 4 POTENTIALLY LIFE-THREATENING
**Arrhythmia (by ECG or physical examination)** ***Specify type, if applicable***	No symptoms AND No intervention indicated	No symptoms AND Non-urgent intervention indicated	Non-life-threatening symptoms AND Non-urgent intervention indicated	Life-threatening arrhythmia OR Urgent intervention indicated
**Blood Pressure Abnormalities1** ***Hypertension (with the lowest reading taken after repeat testing during a visit)*** ***≥ 18 years of age***	140 to < 160 mmHg systolic OR 90 to < 100 mmHg diastolic	≥ 160 to < 180 mmHg systolic OR ≥ 100 to < 110 mmHg diastolic	≥ 180 mmHg systolic OR ≥ 110 mmHg diastolic	Life-threatening consequences in a participant not previously diagnosed with hypertension (e.g., malignant hypertension) OR Hospitalization indicated
< 18 years of age	> 120/80 mmHg	≥ 95th to < 99th percentile + 5 mmHg adjusted for age, height, and gender (systolic and/or diastolic)	≥ 99th percentile + 5 mmHg adjusted for age, height, and gender (systolic and/or diastolic)	Life-threatening consequences in a participant not previously diagnosed with hypertension (e.g., malignant hypertension) OR Hospitalization indicated

PARAMETER	GRADE 1 MILD	GRADE 2 MODERATE	GRADE 3 SEVERE	GRADE 4 POTENTIALLY LIFE-THREATENING
Hypotension	No symptoms	Symptoms corrected with oral fluid replacement	Symptoms AND IV fluids indicated	Shock requiring use of vasopressors or mechanical assistance to maintain blood pressure
**Cardiac Ischemia or Infarction** ***Report only one***	NA	NA	New symptoms with ischemia (stable angina) OR New testing consistent with ischemia	Unstable angina OR Acute myocardial infarction
**Heart Failure**	No symptoms AND Laboratory or cardiac imaging abnormalities	Symptoms with mild to moderate activity or exertion	Symptoms at rest or with minimal activity or exertion (e.g., hypoxemia) OR Intervention indicated (e.g., oxygen)	Life-threatening consequences OR Urgent intervention indicated (e.g., vasoactive medications, ventricular assist device, heart transplant)
**Hemorrhage (with significant acute blood loss)**	NA	Symptoms AND No transfusion indicated	Symptoms AND Transfusion of ≤ 2 units packed RBCs indicated	Life-threatening hypotension OR Transfusion of > 2 units packed RBCs (for children, packed RBCs > 10 cc/kg) indicated
**Prolonged PR Interval or AV Block** ***Report only one*** ***> 16 years of age***	PR interval 0.21 to < 0.25 seconds	PR interval ≥ 0.25 seconds OR Type I 2nd degree AV block	Type II 2nd degree AV block OR Ventricular pause ≥ 3.0 seconds	Complete AV block
≤ 16 years of age	1st degree AV block (PR interval > normal for age and rate)	Type I 2nd degree AV block	Type II 2nd degree AV block OR Ventricular pause ≥ 3.0 seconds	Complete AV block
**Prolonged QTc Interval**	0.45 to 0.47 seconds	> 0.47 to 0.50 seconds	> 0.50 seconds OR ≥ 0.06 seconds above baseline	Life-threatening consequences (e.g., Torsade de pointes, other associated serious ventricular dysrhythmia)
**Thrombosis or Embolism** ***Report only one***	NA	Symptoms AND No intervention indicated	Symptoms AND Intervention indicated	Life-threatening embolic event (e.g., pulmonary embolism, thrombus)

DERMATOLOGIC

PARAMETER	GRADE 1 MILD	GRADE 2 MODERATE	GRADE 3 SEVERE	GRADE 4 POTENTIALLY LIFE-THREATENING
**Alopecia (scalp only)**	Detectable by study participant, caregiver, or physician AND Causing no or minimal interference with usual social & functional activities	Obvious on visual inspection AND Causing greater than minimal interference with usual social & functional activities	NA	NA
**Bruising**	Localized to one area	Localized to more than one area	Generalized	NA
**Cellulitis**	NA	Non-parenteral treatment indicated (e.g., oral antibiotics, antifungals, antivirals)	IV treatment indicated (e.g., IV antibiotics, antifungals, antivirals)	Life-threatening consequences (e.g., sepsis, tissue necrosis)
**Hyperpigmentation**	Slight or localized causing no or minimal interference with usual social & functional activities	Marked or generalized causing greater than minimal interference with usual social & functional activities	NA	NA
**Hypopigmentation**	Slight or localized causing no or minimal interference with usual social & functional activities	Marked or generalized causing greater than minimal interference with usual social & functional activities	NA	NA
**Petechiae**	Localized to one area	Localized to more than one area	Generalized	NA
**Pruritus3 (without skin lesions)**	Itching causing no or minimal interference with usual social & functional activities	Itching causing greater than minimal interference with usual social & functional activities	Itching causing inability to perform usual social & functional activities	NA
**Rash** ***Specify type, if applicable***	Localized rash	Diffuse rash OR Target lesions	Diffuse rash AND Vesicles or limited number of bullae or superficial ulcerations of mucous membrane limited to one site	Extensive or generalized bullous lesions OR Ulceration of mucous membrane involving two or more distinct mucosal sites OR Stevens-Johnson syndrome OR Toxic epidermal necrolysis

ENDOCRINE AND METABOLIC

PARAMETER	GRADE 1 MILD	GRADE 2 MODERATE	GRADE 3 SEVERE	GRADE 4 POTENTIALLY LIFE-THREATENING
**Diabetes Mellitus**	Controlled without medication	Controlled with medication OR	Uncontrolled despite treatment modification	Life-threatening consequences (e.g.,

		Modification of current medication regimen	OR Hospitalization for immediate glucose control indicated	ketoacidosis, hyperosmolar non-ketotic coma, end organ failure)
**Gynecomastia**	Detectable by study participant, caregiver, or physician AND Causing no or minimal interference with usual social & functional activities	Obvious on visual inspection AND Causing pain with greater than minimal interference with usual social & functional activities	Disfiguring changes AND Symptoms requiring intervention or causing inability to perform usual social & functional activities	NA
**Hyperthyroidism**	No symptoms AND Abnormal laboratory value	Symptoms causing greater than minimal interference with usual social & functional activities OR Thyroid suppression therapy indicated	Symptoms causing inability to perform usual social & functional activities OR Uncontrolled despite treatment modification	Life-threatening consequences (e.g., thyroid storm)
**Hypothyroidism**	No symptoms AND Abnormal laboratory value	Symptoms causing greater than minimal interference with usual social & functional activities OR Thyroid replacement therapy indicated	Symptoms causing inability to perform usual social & functional activities OR Uncontrolled despite treatment modification	Life-threatening consequences (e.g., myxedema coma)
**Lipoatrophy**	Detectable by study participant, caregiver, or physician AND Causing no or minimal interference with usual social & functional activities	Obvious on visual inspection AND Causing greater than minimal interference with usual social & functional activities	Disfiguring changes	NA
**Lipohypertrophy**	Detectable by study participant, caregiver, or physician AND Causing no or minimal interference with usual social & functional activities	Obvious on visual inspection AND Causing greater than minimal interference with usual social & functional activities	Disfiguring changes	NA

GASTROINTESTINAL

PARAMETER	GRADE 1 MILD	GRADE 2 MODERATE	GRADE 3 SEVERE	GRADE 4 POTENTIALLY LIFE-THREATENING
**Anorexia**	Loss of appetite without decreased oral intake	Loss of appetite associated with decreased oral intake without significant weight loss	Loss of appetite associated with significant weight loss	Life-threatening consequences OR Aggressive intervention indicated (e.g., tube feeding, total parenteral nutrition)

**Ascites**	No symptoms	Symptoms AND Intervention indicated (e.g., diuretics, therapeutic paracentesis)	Symptoms recur or persist despite intervention	Life-threatening consequences
**Bloating or Distension** ***Report only one***	Symptoms causing no or minimal interference with usual social & functional activities	Symptoms causing greater than minimal interference with usual social & functional activities	Symptoms causing inability to perform usual social & functional activities	NA
**Cholecystitis**	NA	Symptoms AND Medical intervention indicated	Radiologic, endoscopic, or operative intervention indicated	Life-threatening consequences (e.g., sepsis, perforation)
**Constipation**	NA	Persistent constipation requiring regular use of dietary modifications, laxatives, or enemas	Obstipation with manual evacuation indicated	Life-threatening consequences (e.g., obstruction)
**Diarrhea ≥ 1** ***year of age***	Transient or intermittent episodes of unformed stools OR Increase of ≤ 3 stools over baseline per 24-hour period	Persistent episodes of unformed to watery stools OR Increase of 4 to 6 stools over baseline per 24-hour period	Increase of ≥ 7 stools per 24-hour period OR IV fluid replacement indicated	Life-threatening consequences (e.g., hypotensive shock)
< 1 year of age	Liquid stools (more unformed than usual) but usual number of stools	Liquid stools with increased number of stools OR Mild dehydration	Liquid stools with moderate dehydration	Life-threatening consequences (e.g., liquid stools resulting in severe dehydration, hypotensive shock)
**Dysphagia or Odynophagia** ***Report only one and specify location***	Symptoms but able to eat usual diet	Symptoms causing altered dietary intake with no intervention indicated	Symptoms causing severely altered dietary intake with intervention indicated	Life-threatening reduction in oral intake
**Gastrointestinal Bleeding**	Not requiring intervention other than iron supplement	Endoscopic intervention indicated	Transfusion indicated	Life-threatening consequences (e.g., hypotensive shock)
**PARAMETER**	**GRADE 1 MILD**	**GRADE 2 MODERATE**	**GRADE 3 SEVERE**	**GRADE 4 POTENTIALLY LIFE-THREATENING**
**Mucositis or Stomatitis** ***Report only one and specify location***	Mucosal erythema	Patchy pseudomembranes or ulcerations	Confluent pseudomembranes or ulcerations OR Mucosal bleeding with minor trauma	Life-threatening consequences (e.g., aspiration, choking) OR Tissue necrosis OR Diffuse spontaneous mucosal bleeding
**Nausea**	Transient (< 24 hours) or intermittent AND No or minimal interference with oral intake	Persistent nausea resulting in decreased oral intake for 24 to 48 hours	Persistent nausea resulting in minimal oral intake for > 48 hours OR Rehydration indicated (e.g., IV fluids)	Life-threatening consequences (e.g., hypotensive shock)
**Pancreatitis**	NA	Symptoms with hospitalization not indicated	Symptoms with hospitalization indicated	Life-threatening consequences (e.g.,

				circulatory failure, hemorrhage, sepsis)
**Perforation (colon or rectum)**	NA	NA	Intervention indicated	Life-threatening consequences
**Proctitis**	Rectal discomfort with no intervention indicated	Symptoms causing greater than minimal interference with usual social & functional activities OR Medical intervention indicated	Symptoms causing inability to perform usual social & functional activities OR Operative intervention indicated	Life-threatening consequences (e.g., perforation)
**Rectal Discharge**	Visible discharge	Discharge requiring the use of pads	NA	NA
**Vomiting**	Transient or intermittent AND No or minimal interference with oral intake	Frequent episodes with no or mild dehydration	Persistent vomiting resulting in orthostatic hypotension OR Aggressive rehydration indicated (e.g., IV fluids)	Life-threatening consequences (e.g., hypotensive shock)

MUSCULOSKELETAL

PARAMETER	GRADE 1 MILD	GRADE 2 MODERATE	GRADE 3 SEVERE	GRADE 4 POTENTIALLY LIFE-THREATENING
**Arthralgia**	Joint pain causing no or minimal interference with usual social & functional activities	Joint pain causing greater than minimal interference with usual social & functional activities	Joint pain causing inability to perform usual social & functional activities	Disabling joint pain causing inability to perform basic self-care functions
**Arthritis**	Stiffness or joint swelling causing no or minimal interference with usual social & functional activities	Stiffness or joint swelling causing greater than minimal interference with usual social & functional activities	Stiffness or joint swelling causing inability to perform usual social & functional activities	Disabling joint stiffness or swelling causing inability to perform basic self-care functions
**Myalgia (generalized)**	Muscle pain causing no or minimal interference with usual social & functional activities	Muscle pain causing greater than minimal interference with usual social & functional activities	Muscle pain causing inability to perform usual social & functional activities	Disabling muscle pain causing inability to perform basic self-care functions
**Osteonecrosis**	NA	No symptoms but with radiographic findings AND No operative intervention indicated	Bone pain with radiographic findings OR Operative intervention indicated	Disabling bone pain with radiographic findings causing inability to perform basic self-care functions
**Osteopenia6 ≥ *30 years of age***	BMD t-score -2.5 to -1	NA	NA	NA
< 30 years of age	BMD z-score -2 to -1	NA	NA	NA
**Osteoporosis6 *≥ 30 years of age***	NA	BMD t-score < -2.5	Pathologic fracture (e.g., compression fracture causing loss of vertebral height)	Pathologic fracture causing life-threatening consequences
< 30 years of age	NA	BMD z-score < -2	Pathologic fracture (e.g., compression fracture causing loss of vertebral height)	Pathologic fracture causing life-threatening consequences

NEUROLOGIC

PARAMETER	GRADE 1 MILD	GRADE 2 MODERATE	GRADE 3 SEVERE	GRADE 4 POTENTIALLY LIFE-THREATENING
**Acute CNS Ischemia**	NA	NA	Transient ischemic attack	Cerebral vascular accident (e.g., stroke with neurological deficit)
**Altered Mental Status (for Dementia, see *Cognitive, Behavioral, or Attentional Disturbance* below)**	Changes causing no or minimal interference with usual social & functional activities	Mild lethargy or somnolence causing greater than minimal interference with usual social & functional activities	Confusion, memory impairment, lethargy, or somnolence causing inability to perform usual social & functional activities	Delirium OR Obtundation OR Coma
**Ataxia**	Symptoms causing no or minimal interference with usual social & functional activities OR No symptoms with ataxia detected on examination	Symptoms causing greater than minimal interference with usual social & functional activities	Symptoms causing inability to perform usual social & functional activities	Disabling symptoms causing inability to perform basic self-care functions
**Cognitive, Behavioral, or Attentional Disturbance (includes dementia and attention deficit disorder)** ***Specify type, if applicable***	Disability causing no or minimal interference with usual social & functional activities OR Specialized resources not indicated	Disability causing greater than minimal interference with usual social & functional activities OR Specialized resources on part-time basis indicated	Disability causing inability to perform usual social & functional activities OR Specialized resources on a full-time basis indicated	Disability causing inability to perform basic self-care functions OR Institutionalization indicated
**Developmental Delay *< 18 years of age*** ***Specify type, if applicable***	Mild developmental delay, either motor or cognitive, as determined by comparison with a developmental screening tool appropriate for the setting	Moderate developmental delay, either motor or cognitive, as determined by comparison with a developmental screening tool appropriate for the setting	Severe developmental delay, either motor or cognitive, as determined by comparison with a developmental screening tool appropriate for the setting	Developmental regression, either motor or cognitive, as determined by comparison with a developmental screening tool appropriate for the setting
**Headache**	Symptoms causing no or minimal interference with usual social & functional activities	Symptoms causing greater than minimal interference with usual social & functional activities	Symptoms causing inability to perform usual social & functional activities	Symptoms causing inability to perform basic self-care functions OR Hospitalization indicated OR Headache with significant impairment of alertness or other neurologic function
**PARAMETER**	**GRADE 1 MILD**	**GRADE 2 MODERATE**	**GRADE 3 SEVERE**	**GRADE 4 POTENTIALLY LIFE-THREATENING**

**Neuromuscular Weakness (includes myopathy and neuropathy)** ***Specify type, if applicable***	Minimal muscle weakness causing no or minimal interference with usual social & functional activities OR No symptoms with decreased strength on examination	Muscle weakness causing greater than minimal interference with usual social & functional activities	Muscle weakness causing inability to perform usual social & functional activities	Disabling muscle weakness causing inability to perform basic self-care functions OR Respiratory muscle weakness impairing ventilation
**Neurosensory Alteration (includes paresthesia and painful neuropathy)** ***Specify type, if applicable***	Minimal paresthesia causing no or minimal interference with usual social & functional activities OR No symptoms with sensory alteration on examination	Sensory alteration or paresthesia causing greater than minimal interference with usual social & functional activities	Sensory alteration or paresthesia causing inability to perform usual social & functional activities	Disabling sensory alteration or paresthesia causing inability to perform basic self-care functions
**Seizures** ***New Onset Seizure ≥ 18 years of age***	NA	NA	1 to 3 seizures	Prolonged and repetitive seizures (e.g., status epilepticus) OR Difficult to control (e.g., refractory epilepsy)
< 18 years of age (includes new or pre-existing febrile seizures)	Seizure lasting < 5 minutes with < 24 hours postictal state	Seizure lasting 5 to < 20 minutes with < 24 hours postictal state	Seizure lasting ≥ 20 minutes OR > 24 hours postictal state	Prolonged and repetitive seizures (e.g., status epilepticus) OR Difficult to control (e.g., refractory epilepsy)
Pre-existing Seizure	NA	Increased frequency from previous level of control without change in seizure character	Change in seizure character either in duration or quality (e.g., severity or focality)	Prolonged and repetitive seizures (e.g., status epilepticus) OR Difficult to control (e.g., refractory epilepsy)
**Syncope**	Near syncope without loss of consciousness (e.g., pre-syncope)	Loss of consciousness with no intervention indicated	Loss of consciousness AND Hospitalization or intervention required	NA

PREGNANCY, PUERPERIUM AND PERINATAL

PARAMETER	GRADE 1 MILD	GRADE 2 MODERATE	GRADE 3 SEVERE	GRADE 4 POTENTIALLY LIFE-THREATENING
**Fetal Death or Stillbirth (report using mother's participant ID)** ***Report only one***	**NA**	NA	Fetal loss occurring at ≥ 20 weeks gestation	NA
**Preterm Delivery7 (report using mother's participant ID)**	**Delivery at 34 to < 37 weeks gestational age**	Delivery at 28 to < 34 weeks gestational age	Delivery at 24 to < 28 weeks gestational age	Delivery at < 24 weeks gestational age
**Spontaneous Abortion or Miscarriage8 (report using mother's participant ID)** ***Report only one***	**Chemical pregnancy**	Uncomplicated spontaneous abortion or miscarriage	Complicated spontaneous abortion or miscarriage	NA

PSYCHIATRIC

PARAMETER	GRADE 1 MILD	GRADE 2 MODERATE	GRADE 3 SEVERE	GRADE 4 POTENTIALLY LIFE-THREATENING
**Insomnia**	Mild difficulty falling asleep, staying asleep, or waking up early	Moderate difficulty falling asleep, staying asleep, or waking up early	Severe difficulty falling asleep, staying asleep, or waking up early	NA
**Psychiatric Disorders (includes anxiety, depression, mania, and psychosis)** ***Specify disorder***	Symptoms with intervention not indicated OR Behavior causing no or minimal interference with usual social & functional activities	Symptoms with intervention indicated OR Behavior causing greater than minimal interference with usual social & functional activities	Symptoms with hospitalization indicated OR Behavior causing inability to perform usual social & functional activities	Threatens harm to self or others OR Acute psychosis OR Behavior causing inability to perform basic self-care functions
**Suicidal Ideation or Attempt** ***Report only one***	Preoccupied with thoughts of death AND No wish to kill oneself	Preoccupied with thoughts of death AND Wish to kill oneself with no specific plan or intent	Thoughts of killing oneself with partial or complete plans but no attempt to do so OR Hospitalization indicated	Suicide attempted

RESPIRATORY

PARAMETER	GRADE 1 MILD	GRADE 2 MODERATE	GRADE 3 SEVERE	GRADE 4 POTENTIALLY LIFE-THREATENING
**Acute Bronchospasm**	Forced expiratory volume in 1 second or peak flow reduced to ≥ 70 to < 80% OR Mild symptoms with intervention not indicated	Forced expiratory volume in 1 second or peak flow 50 to < 70% OR Symptoms with intervention indicated OR Symptoms causing greater than minimal interference with usual social & functional activities	Forced expiratory volume in 1 second or peak flow 25 to < 50% OR Symptoms causing inability to perform usual social & functional activities	Forced expiratory volume in 1 second or peak flow < 25% OR Life-threatening respiratory or hemodynamic compromise OR Intubation
**Dyspnea or Respiratory Distress** ***Report only one***	Dyspnea on exertion with no or minimal interference with usual social & functional activities OR Wheezing OR Minimal increase in respiratory rate for age	Dyspnea on exertion causing greater than minimal interference with usual social & functional activities OR Nasal flaring OR Intercostal retractions OR Pulse oximetry 90 to < 95%	Dyspnea at rest causing inability to perform usual social & functional activities OR Pulse oximetry < 90%	Respiratory failure with ventilator support indicated (e.g., CPAP, BPAP, intubation)

SENSORY

PARAMETER	GRADE 1 MILD	GRADE 2 MODERATE	GRADE 3 SEVERE	GRADE 4 POTENTIALLY LIFE-THREATENING
**Hearing Loss ≥** ***12 years of age***	NA	Hearing aid or intervention not indicated	Hearing aid or intervention indicated	Profound bilateral hearing loss (> 80 dB at 2 kHz and above) OR Non-serviceable hearing (i.e., >50 dB audiogram and <50% speech discrimination)

PARAMETER	GRADE 1 MILD	GRADE 2 MODERATE	GRADE 3 SEVERE	GRADE 4 POTENTIALLY LIFE-THREATENING
< 12 years of age (based on a 1, 2, 3, 4, 6 and 8 kHz audiogram)	> 20 dB hearing loss at ≤ 4 kHz	> 20 dB hearing loss at > 4 kHz	> 20 dB hearing loss at ≥ 3 kHz in one ear with additional speech language related services indicated (where available) OR Hearing loss sufficient to indicate	Audiologic indication for cochlear implant and additional speech-language related services indicated (where available)

			therapeutic intervention, including hearing aids	
**Tinnitus**	Symptoms causing no or minimal interference with usual social & functional activities with intervention not indicated	Symptoms causing greater than minimal interference with usual social & functional activities with intervention indicated	Symptoms causing inability to perform usual social & functional activities	NA
**Uveitis**	No symptoms AND Detectable on examination	Anterior uveitis with symptoms OR medical intervention indicated	Posterior or pan-uveitis OR Operative intervention indicated	Disabling visual loss in affected eye(s)
**Vertigo**	Vertigo causing no or minimal interference with usual social & functional activities	Vertigo causing greater than minimal interference with usual social & functional activities	Vertigo causing inability to perform usual social & functional activities	Disabling vertigo causing inability to perform basic self-care functions
**Visual Changes (assessed from baseline)**	Visual changes causing no or minimal interference with usual social & functional activities	Visual changes causing greater than minimal interference with usual social & functional activities	Visual changes causing inability to perform usual social & functional activities	Disabling visual loss in affected eye(s)

SYSTEMIC

PARAMETER	GRADE 1 MILD	GRADE 2 MODERATE	GRADE 3 SEVERE	GRADE 4 POTENTIALLY LIFE-THREATENING
**Acute Allergic Reaction**	Localized urticaria (wheals) with no medical intervention indicated	Localized urticaria with intervention indicated OR Mild angioedema with no intervention indicated	Generalized urticaria OR Angioedema with intervention indicated OR Symptoms of mild bronchospasm	Acute anaphylaxis OR Life-threatening bronchospasm OR Laryngeal edema
**Chills**	Symptoms causing no or minimal interference with usual social & functional activities	Symptoms causing greater than minimal interference with usual social & functional activities	Symptoms causing inability to perform usual social & functional activities	NA
**Cytokine Release Syndrome**	Mild signs and symptoms AND Therapy (i.e., antibody infusion) interruption not indicated	Therapy (i.e., antibody infusion) interruption indicated AND Responds promptly to symptomatic treatment OR Prophylactic medications indicated for ≤ 24 hours	Prolonged severe signs and symptoms OR Recurrence of symptoms following initial improvement	Life-threatening consequences (e.g., requiring pressor or ventilator support)
**Fatigue or Malaise** ***Report only one***	Symptoms causing no or minimal interference with usual social & functional activities	Symptoms causing greater than minimal interference with usual social & functional activities	Symptoms causing inability to perform usual social & functional activities	Incapacitating symptoms of fatigue or malaise causing inability to perform basic self-care functions
**Fever (non-axillary temperatures only)**	38.0 to < 38.6°C or 100.4 to < 101.5°F	≥ 38.6 to < 39.3°C or ≥ 101.5 to < 102.7°F	≥ 39.3 to < 40.0°C or ≥ 102.7 to < 104.0°F	≥ 40.0°C or ≥ 104.0°F
**Pain10 (not associated with study agent injections and not specified elsewhere)** ***Specify location***	Pain causing no or minimal interference with usual social & functional activities	Pain causing greater than minimal interference with usual social & functional activities	Pain causing inability to perform usual social & functional activities	Disabling pain causing inability to perform basic self-care functions OR Hospitalization indicated
**Serum Sickness**	Mild signs and symptoms	Moderate signs and symptoms AND Intervention indicated (e.g., antihistamines)	Severe signs and symptoms AND Higher level intervention indicated (e.g., steroids or IV fluids)	Life-threatening consequences (e.g., requiring pressor or ventilator support)
**Underweight12** ***> 5 to 19 years of age***	NA	WHO BMI z-score < -2 to ≤ -3	WHO BMI z-score < -3	WHO BMI z-score < -3 with life-threatening consequences

PARAMETER	GRADE 1 MILD	GRADE 2 MODERATE	GRADE 3 SEVERE	GRADE 4 POTENTIALLY LIFE-THREATENING

2 to 5 years of age	NA	WHO Weight-for-height z-score < -2 to ≤ -3	WHO Weight-for-height z-score < -3	WHO Weight-for-height z-score < -3 with life-threatening consequences
< 2 years of age	NA	WHO Weight-for-length z-score < -2 to ≤ -3	WHO Weight-for-length z-score < -3	WHO Weight-for-length z-score < -3 with life-threatening consequences
**Weight Loss (excludes postpartum weight loss)**	NA	5 to < 9% loss in body weight from baseline	≥ 9 to < 20% loss in body weight from baseline	≥ 20% loss in body weight from baseline OR Aggressive intervention indicated (e.g., tube feeding, total parenteral nutrition)

URINARY

PARAMETER	GRADE 1 MILD	GRADE 2 MODERATE	GRADE 3 SEVERE	GRADE 4 POTENTIALLY LIFE-THREATENING
**Urinary Tract Obstruction**	NA	Signs or symptoms of urinary tract obstruction without hydronephrosis or renal dysfunction	Signs or symptoms of urinary tract obstruction with hydronephrosis or renal dysfunction	Obstruction causing life-threatening consequences

SITE REACTIONS TO INJECTIONS AND INFUSIONS

PARAMETE R	GRADE 1 MILD	GRADE 2 MODERATE	GRADE 3 SEVERE	GRADE 4 POTENTIALLY LIFE-THREATENING
**Injection Site Pain or Tenderness** ***Report only one***	Pain or tenderness causing no or minimal limitation of use of limb	Pain or tenderness causing greater than minimal limitation of use of limb	Pain or tenderness causing inability to perform usual social & functional activities	Pain or tenderness causing inability to perform basic self-care function OR Hospitalization indicated
**Injection Site Erythema or Redness13** ***Report only one*** ***> 15 years of age***	2.5 to < 5 cm in diameter OR 6.25 to < 25 cm2 surface area AND Symptoms causing no or minimal interference with usual social & functional activities	≥ 5 to < 10 cm in diameter OR ≥ 25 to < 100 cm2 surface area OR Symptoms causing greater than minimal interference with usual social & functional activities	≥ 10 cm in diameter OR ≥ 100 cm2 surface area OR Ulceration OR Secondary infection OR Phlebitis OR Sterile abscess OR Drainage OR Symptoms causing inability to perform usual social & functional activities	Potentially life-threatening consequences (e.g., abscess, exfoliative dermatitis, necrosis involving dermis or deeper tissue)
≤ 15 years of age	≤ 2.5 cm in diameter	> 2.5 cm in diameter with < 50% surface area of the extremity segment involved (e.g., upper arm or thigh)	≥ 50% surface area of the extremity segment involved (e.g., upper arm or thigh) OR Ulceration OR Secondary infection OR Phlebitis OR Sterile abscess OR Drainage	Potentially life-threatening consequences (e.g., abscess, exfoliative dermatitis, necrosis involving dermis or deeper tissue)
**Injection Site Induration or Swelling** ***Report only one*** ***> 15 years of age***	Same as for **Injection Site Erythema or Redness,** *> 15 years of age*	Same as for **Injection Site Erythema or Redness,** *> 15 years of age*	Same as for **Injection Site Erythema or Redness,** *> 15 years of age*	Same as for **Injection Site Erythema or Redness,** *> 15 years of age*
≤ 15 years of age	Same as for **Injection Site Erythema or Redness,** *≤ 15 years of age*	Same as for **Injection Site Erythema or Redness,** *≤ 15 years of age*	Same as for **Injection Site Erythema or Redness,** *≤ 15 years of age*	Same as for **Injection Site Erythema or Redness,** *≤ 15 years of age*
**Injection Site Pruritus**	Itching localized to the injection site that is relieved spontaneously or in < 48 hours of treatment	Itching beyond the injection site that is not generalized OR Itching localized to the injection site requiring ≥ 48 hours treatment	Generalized itching causing inability to perform usual social & functional activities	NA

2. Laboratory values

CHEMISTRIES

PARAMETER	GRADE 1 MILD	GRADE 2 MODERATE	GRADE 3 SEVERE	GRADE 4 POTENTIALLY LIFE-THREATENING
**Acidosis**	NA	pH ≥ 7.3 to < LLN	pH < 7.3 without life-threatening consequences	pH < 7.3 with life-threatening consequences
**Albumin, Low (g/dL; *g/L*)**	3.0 to < LLN *30 to < LLN*	≥ 2.0 to < 3.0 *≥ 20 to < 30*	< 2.0 *< 20*	NA
**Alkaline Phosphatase, High**	1.25 to < 2.5 x ULN	2.5 to < 5.0 x ULN	5.0 to < 10.0 x ULN	≥ 10.0 x ULN
**Alkalosis**	NA	pH > ULN to ≤ 7.5	pH > 7.5 without life-threatening consequences	pH > 7.5 with life-threatening consequences
**ALT or SGPT, High** ***Report only one***	1.25 to < 2.5 x ULN	2.5 to < 5.0 x ULN	5.0 to < 10.0 x ULN	≥ 10.0 x ULN
**Amylase (Pancreatic) or Amylase (Total), High** ***Report only one***	1.1 to < 1.5 x ULN	1.5 to < 3.0 x ULN	3.0 to < 5.0 x ULN	≥ 5.0 x ULN
**AST or SGOT, High** ***Report only one***	1.25 to < 2.5 x ULN	2.5 to < 5.0 x ULN	5.0 to < 10.0 x ULN	≥ 10.0 x ULN
**Bicarbonate, Low (mEq/L; *mmol/L*)**	16.0 to < LLN *16.0 to < LLN*	11.0 to < 16.0 *11.0 to < 16.0*	8.0 to < 11.0 *8.0 to < 11.0*	< 8.0 *< 8.0*
**Bilirubin** ***Direct Bilirubin14, High*** ***> 28 days of age***	NA	NA	> ULN	> ULN with life-threatening consequences (e.g., signs and symptoms of liver failure)
≤ 28 days of age	ULN to ≤ 1 mg/dL	> 1 to ≤ 1.5 mg/dL	> 1.5 to ≤ 2 mg/dL	> 2 mg/dL
Total Bilirubin, High ***> 28 days of age***	1.1 to < 1.6 x ULN	1.6 to < 2.6 x ULN	2.6 to < 5.0 x ULN	≥ 5.0 x ULN
≤ 28 days of age	See Appendix A. Total Bilirubin for Term and Preterm Neonates	See Appendix A. Total Bilirubin for Term and Preterm Neonates	See Appendix A. Total Bilirubin for Term and Preterm Neonates	See Appendix A. Total Bilirubin for Term and Preterm Neonates
**Calcium, High (mg/dL; *mmol/L*)** ***≥ 7 days of age***	10.6 to < 11.5 *2.65* to *< 2.88*	11.5 to < 12.5 *2.88* to < *3.13*	12.5 to < 13.5 *3.13* to < *3.38*	≥ 13.5 ≥ *3.38*
< 7 days of age	11.5 to < 12.4 *2.88* to < *3.10*	12.4 to < 12.9 *3.10* to < *3.23*	12.9 to < 13.5 *3.23* to < *3.38*	≥ 13.5 ≥ *3.38*
**Calcium (Ionized), High**	> ULN to < 6.0 > *ULN to < 1.5*	6.0 to < 6.4 *1.5 to < 1.6*	6.4 to < 7.2 *1.6 to < 1.8*	≥ 7.2 ≥ *1.8*

**(mg/dL; *mmol/L*)**				
**PARAMETER**	**GRADE 1 MILD**	**GRADE 2 MODERATE**	**GRADE 3 SEVERE**	**GRADE 4 POTENTIALLY LIFE-THREATENING**
**Calcium, Low (mg/dL; *mmol/L*) *≥ 7 days of age***	7.8 to < 8.4 *1.95* to < *2.10*	7.0 to < 7.8 *1.75* to < *1.95*	6.1 to < 7.0 *1.53* to < *1.75*	< 6.1 *< 1.53*
< 7 days of age	6.5 to < 7.5 *1.63* to < *1.88*	6.0 to < 6.5 *1.50* to < *1.63*	5.50 to < 6.0 *1.38* to < *1.50*	< 5.50 < *1.38*
**Calcium (Ionized), Low (mg/dL; *mmol/L*)**	< LLN to 4.0 *< LLN to 1.0*	3.6 to < 4.0 *0.9 to < 1.0*	3.2 to < 3.6 *0.8 to < 0.9*	< 3.2 *< 0.8*
**Cardiac Troponin I, High**	NA	NA	NA	Levels consistent with myocardial infarction or unstable angina as defined by the local laboratory
**Creatine Kinase, High**	3 to < 6 x ULN	6 to < 10 x ULN	10 to < 20 x ULN	≥ 20 x ULN
**Creatinine, High**	1.1 to 1.3 x ULN	> 1.3 to 1.8 x ULN OR Increase of > 0.3 mg/dL above baseline	> 1.8 to < 3.5 x ULN OR Increase of 1.5 to < 2.0 x above baseline	≥ 3.5 x ULN OR Increase of ≥ 2.0 x above baseline
**Creatinine Clearance or eGFR, Low *Report only one***	NA	< 90 to 60 ml/min or ml/min/1.73 m2 OR 10 to < 30% decrease from baseline	< 60 to 30 ml/min or ml/min/1.73 m2 OR ≥ 30 to < 50% decrease from baseline	< 30 ml/min or ml/min/1.73 m2 OR ≥ 50% decrease from baseline or dialysis needed
**Glucose (mg/dL; *mmol/L*) *Fasting, High***	110 to 125 *6.11 to < 6.95*	> 125 to 250 *6.95 to < 13.89*	> 250 to 500 *13.89 to < 27.75*	> 500 ≥ *27.75*
Nonfasting, High	116 to 160 *6.44 to < 8.89*	> 160 to 250 *8.89 to < 13.89*	> 250 to 500 *13.89 to < 27.75*	> 500 ≥ *27.75*
**Glucose, Low (mg/dL; *mmol/L*) *≥ 1 month of age***	55 to 64 *3.05 to 3.55*	40 to < 55 *2.22 to < 3.05*	30 to < 40 *1.67 to < 2.22*	< 30 < *1.67*
< 1 month of age	50 to 54 *2.78 to 3.00*	40 to < 50 *2.22 to < 2.78*	30 to < 40 *1.67 to < 2.22*	< 30 < *1.67*
**Lactate, High**	ULN to < 2.0 x ULN without acidosis	≥ 2.0 x ULN without acidosis	Increased lactate with pH < 7.3 without life-threatening consequences	Increased lactate with pH < 7.3 with life-threatening consequences
**Lipase, High**	1.1 to < 1.5 x ULN	1.5 to < 3.0 x ULN	3.0 to < 5.0 x ULN	≥ 5.0 x ULN
**Lipid Disorders (mg/dL; *mmol/L*) *Cholesterol, Fasting, High***	200 to < 240 *5.18 to < 6.19*	240 to < 300 *6.19 to < 7.77*	≥ 300 ≥ *7.77*	NA

≥ 18 years of age				
**PARAMETER**	**GRADE 1 MILD**	**GRADE 2 MODERATE**	**GRADE 3 SEVERE**	**GRADE 4 POTENTIALLY LIFE-THREATENING**
< 18 years of age	170 to < 200 *4.40 to < 5.15*	200 to < 300 *5.15 to < 7.77*	≥ 300 ≥ *7.77*	NA
LDL, Fasting, High ≥ 18 years of age	130 to < 160 *3.37 to < 4.12*	160 to < 190 *4.12 to < 4.90*	≥ 190 ≥ *4.90*	NA
> 2 to < 18 years of age	110 to < 130 *2.85 to < 3.34*	130 to < 190 *3.34 to < 4.90*	≥ 190 ≥ *4.90*	NA
Triglycerides, Fasting, High	150 to 300 *1.71 to 3.42*	>300 to 500 *>3.42 to 5.7*	>500 to < 1,000 *>5.7 to 11.4*	> 1,000 *> 11.4*
**Magnesium16, Low (mEq/L; *mmol/L*)**	1.2 to < 1.4 *0.60 to < 0.70*	0.9 to < 1.2 *0.45 to < 0.60*	0.6 to < 0.9 *0.30 to < 0.45*	< 0.6 *< 0.30*
**Phosphate, Low (mg/dL; *mmol/L*) *> 14 years of age***	2.0 to < LLN *0.81 to < LLN*	1.4 to < 2.0 *0.65 to < 0.81*	1.0 to < 1.4 *0.32 to < 0.65*	< 1.0 *< 0.32*
1 to 14 years of age	3.0 to < 3.5 *0.97 to < 1.13*	2.5 to < 3.0 *0.81 to < 0.97*	1.5 to < 2.5 *0.48 to < 0.81*	< 1.5 *< 0.48*
< 1 year of age	3.5 to < 4.5 *1.13 to < 1.45*	2.5 to < 3.5 *0.81 to < 1.13*	1.5 to < 2.5 *0.48 to < 0.81*	< 1.5 *< 0.48*
**Potassium, High (mEq/L; *mmol/L*)**	5.6 to < 6.0 *5.6 to < 6.0*	6.0 to < 6.5 *6.0 to < 6.5*	6.5 to < 7.0 *6.5 to < 7.0*	≥ 7.0 ≥ *7.0*
**Potassium, Low (mEq/L; *mmol/L*)**	3.0 to < 3.4 *3.0 to < 3.4*	2.5 to < 3.0 *2.5 to < 3.0*	2.0 to < 2.5 *2.0 to < 2.5*	< 2.0 *< 2.0*
**Sodium, High (mEq/L; *mmol/L*)**	146 to < 150 *146 to < 150*	150 to < 154 *150 to < 154*	154 to < 160 *154 to < 160*	≥ 160 ≥ *160*
**Sodium, Low (mEq/L; *mmol/L*)**	130 to < 135 *130 to < 135*	125 to < 130 *125 to < 135*	121 to < 125 *121 to < 125*	≤ 120 ≤ *120*
**Uric Acid, High (mg/dL; *mmol/L*)**	7.5 to < 10.0 *0.45 to < 0.59*	10.0 to < 12.0 *0.59 to < 0.71*	12.0 to < 15.0 *0.71 to < 0.89*	≥ 15.0 ≥ *0.89*

HEMATOLOGY

PARAMETER	GRADE 1 MILD	GRADE 2 MODERATE	GRADE 3 SEVERE	GRADE 4 POTENTIALLY LIFE-THREATENING
**Absolute CD4+ Count, Low (cell/mm3; *cells/L*)**	300 to < 400 *300 to < 400*	200 to < 300 *200 to < 300*	100 to < 200 *100 to < 200*	< 100 *< 100*

> 5 years of age (not HIV infected)				

PARAMETER	GRADE 1 MILD	GRADE 2 MODERATE	GRADE 3 SEVERE	GRADE 4 POTENTIALLY LIFE-THREATENING
**Absolute Lymphocyte Count, Low (cell/mm3; *cells/L*)** ***> 5 years of age (not HIV infected)***	600 to < 650 *0.600 x 109 to < 0.650 x 109*	500 to < 600 *0.500 x 109 to < 0.600 x 109*	350 to < 500 *0.350 x 109 to < 0.500 x 109*	< 350 *< 0.350 x 109*
**Absolute Neutrophil Count (ANC), Low (cells/mm3; *cells/L*)** ***> 7 days of age***	800 to 1,000 *0.800 x 109 to 1.000 x 109*	600 to 799 *0.600 x 109 to 0.799 x 109*	400 to 599 *0.400 x 109 to 0.599 x 109*	< 400 *< 0.400 x 109*
2 to 7 days of age	1,250 to 1,500 *1.250 x 109 to 1.500 x 109*	1,000 to 1,249 *1.000 x 109 to 1.249 x 109*	750 to 999 *0.750 x 109 to 0.999 x 109*	< 750 *< 0.750 x 109*
≤ 1 day of age	4,000 to 5,000 *4.000 x 109 to 5.000 x 109*	3,000 to 3,999 *3.000 x 109 to 3.999 x 109*	1,500 to 2,999 *1.500 x 109 to 2.999 x 109*	< 1,500 *< 1.500 x 109*
**Fibrinogen, Decreased (mg/dL; *g/L*)**	100 to < 200 *1.00 to < 2.00* OR 0.75 to < 1.00 x LLN	75 to < 100 *0.75 to < 1.00* OR ≥ 0.50 to < 0.75 x LLN	50 to < 75 *0.50 to < 0.75* OR 0.25 to < 0.50 x LLN	< 50 *< 0.50* OR < 0.25 x LLN OR Associated with gross bleeding
**Hemoglobin17, Low (g/dL; *mmol/L*)18** ***≥ 13 years of age (male only)***	10.0 to 10.9 *6.19 to 6.76*	9.0 to < 10.0 *5.57 to < 6.19*	7.0 to < 9.0 *4.34 to < 5.57*	< 7.0 *< 4.34*
≥ 13 years of age (female only)	9.5 to 10.4 *5.88 to 6.48*	8.5 to < 9.5 *5.25 to < 5.88*	6.5 to < 8.5 *4.03 to < 5.25*	< 6.5 *< 4.03*
57 days of age to < 13 years of age (male and female)	9.5 to 10.4 *5.88 to 6.48*	8.5 to < 9.5 *5.25 to < 5.88*	6.5 to < 8.5 *4.03 to < 5.25*	< 6.5 *< 4.03*
36 to 56 days of age (male and female)	8.5 to 9.6 *5.26 to 5.99*	7.0 to < 8.5 *4.32 to < 5.26*	6.0 to < 7.0 *3.72 to < 4.32*	< 6.0 *< 3.72*
22 to 35 days of age (male and female)	9.5 to 11.0 *5.88 to 6.86*	8.0 to < 9.5 *4.94 to < 5.88*	6.7 to < 8.0 *4.15 to < 4.94*	< 6.7 *< 4.15*
8 to ≤ 21 days of age (male and female)	11.0 to 13.0 *6.81 to 8.10*	9.0 to < 11.0 *5.57 to < 6.81*	8.0 to < 9.0 *4.96 to < 5.57*	< 8.0 *< 4.96*
≤ 7 days of age (male and female)	13.0 to 14.0 *8.05 to 8.72*	10.0 to < 13.0 *6.19 to < 8.05*	9.0 to < 10.0 *5.59 to < 6.19*	< 9.0 *< 5.59*

PARAMETER	GRADE 1 MILD	GRADE 2 MODERATE	GRADE 3 SEVERE	GRADE 4 POTENTIALLY LIFE-THREATENING
**INR, High (not on anticoagulation therapy)**	1.1 to < 1.5 x ULN	1.5 to < 2.0 x ULN	2.0 to < 3.0 x ULN	≥ 3.0 x ULN
**Methemoglobin (% hemoglobin)**	5.0 to < 10.0%	10.0 to < 15.0%	15.0 to < 20.0%	≥ 20.0%
**PTT, High (not on anticoagulation therapy)**	1.1 to < 1.66 x ULN	1.66 to < 2.33 x ULN	2.33 to < 3.00 x ULN	≥ 3.00 x ULN
**Platelets, Decreased (cells/mm3; *cells/L*)**	100,000 to < 124,999 *100.000 x 109 to < 124.999 x 109*	50,000 to < 100,000 *50.000 x 109 to < 100.000 x 109*	25,000 to < 50,000 *25.000 x 109 to < 50.000 x 109*	< 25,000 *< 25.000 x 109*
**PT, High (not on anticoagulation therapy**	1.1 to < 1.25 x ULN	1.25 to < 1.50 x ULN	1.50 to < 3.00 x ULN	≥ 3.00 x ULN
**WBC, Decreased (cells/mm3; *cells/L*) *> 7 days of age***	2,000 to 2,499<br>*2.000 x 109 to 2.499 x 109*	1,500 to 1,999<br>*1.500 x 109 to 1.999 x 109*	1,000 to 1,499<br>*1.000 x 109 to 1.499 x 109*	< 1,000<br>*< 1.000 x 109*
≤ 7 days of age	5,500 to 6,999<br>*5.500 x 109 to 6.999 x 109*	4,000 to 5,499<br>*4.000 x 109 to 5.499 x 109*	2,500 to 3,999<br>*2.500 x 109 to 3.999 x 109*	< 2,500<br>*< 2.500 x 109*

URINALYSIS

PARAMETER	GRADE 1 MILD	GRADE 2 MODERATE	GRADE 3 SEVERE	GRADE 4 POTENTIALLY LIFE-THREATENING
**Glycosuria (random collection tested by dipstick)**	Trace to 1+ or ≤ 250 mg	2+ or > 250 to ≤ 500 mg	> 2+ or > 500 mg	NA
**Hematuria (not to be reported based on dipstick findings or on blood believed to be of menstrual origin)**	6 to < 10 RBCs per high power field	≥ 10 RBCs per high power field	Gross, with or without clots OR With RBC casts OR Intervention indicated	Life-threatening consequences
**Proteinuria (random collection tested by dipstick)**	1+	2+	3+ or higher	NA

APPENDIX A: TOTAL BILIRUBIN TABLE FOR TERM AND PRETERM NEONATES

PARAMETER	GRADE 1 MILD	GRADE 2 MODERATE	GRADE 3 SEVERE	GRADE 4 POTENTIALLY LIFE-THREATENING

**Total Bilirubin19, High (mg/dL; *µmol/L*)20 *Term Neonate21*** ***< 24 hours of age***	4 to < 7 *68.4 to < 119.7*	7 to < 10 *119.7 to < 171*	10 to < 17 *171* to < *290.7*	≥ 17 ≥ *290.7*
24 to < 48 hours of age	5 to < 8 *85.5 to < 136.8*	8 to < 12 *136.8 to < 205.2*	12 to < 19 *205.2 to < 324.9*	≥ 19 ≥ *324.9*
48 to < 72 hours of age	8.5 to < 13 *145.35 to < 222.3*	13 to < 15 *222.3 to < 256.5*	15 to < 22 *256.5 to < 376.2*	≥ 22 ≥ *376.2*
72 hours to < 7 days of age	11 to < 16 *188.1 to < 273.6*	16 to < 18 *273.6 to < 307.8*	18 to < 24 *307.8 to < 410.4*	≥ 24 ≥ *410.4*
7 to 28 days of age (breast feeding)	5 to < 10 *85.5 to < 171*	10 to < 20 *171 to < 342*	20 to < 25 *342 to < 427.5*	≥ 25 ≥ *427.5*
7 to 28 days of age (not breast feeding)	1.1 to < 1.6 x ULN	1.6 to < 2.6 x ULN	2.6 to < 5.0 x ULN	≥ 5.0 x ULN
Preterm Neonate20 35 to < 37 weeks gestational age	Same as for ***Total Bilirubin, High, Term Neonate*** (based on days of age).	Same as for ***Total Bilirubin, High, Term Neonate*** (based on days of age).	Same as for ***Total Bilirubin, High, Term Neonate*** (based on days of age).	Same as for ***Total Bilirubin, High, Term Neonate*** (based on days of age).
32 to < 35 weeks gestational age and < 7 days of age	NA	NA	10 to < 14 *171 to < 239.4*	≥ 14 ≥ *239.4*
28 to < 32 weeks gestational age and < 7 days of age	NA	NA	6 to < 10 *102.6 to < 171*	≥ 10 ≥ *171*
< 28 weeks gestational age and < 7 days of age	NA	NA	5 to < 8 *85.5 to < 136.8*	≥ 8 ≥ *136.8*
7 to 28 days of age (breast feeding)	5 to < 10 *85.5 to < 171*	10 to < 20 *171 to < 342*	20 to < 25 *342 to < 427.5*	≥ 25 ≥ *427.5*
7 to 28 days of age (not breast feeding)	1.1 to < 1.6 x ULN	1.6 to < 2.6 x ULN	2.6 to < 5.0 x ULN	≥ 5.0 x ULN

Statistical Analysis Plan: BREATHE

Final version: Date 15 August 2019

22 Table of Contents

1 Background 62
Trial design 63
2 Objectives............ 63
3 Analysis principles 63
4 Missing data 64
5 Recruitment 64

6 Characteristics of the study population 64
7 Delivery of the intervention (and adherence) 65
8 Sample size considerations 65
9 Outcome variables 66
9.1 Primary outcome 66
9.2 Secondary outcomes 66
9.3 Exploratory outcomes 66
9.4 Reporting of harms 66
10 Analysis methods 67
11 Review of data prior to unmasking 68
12 Process of unmasking 68

Signed

Helen Weiss, Co-Investigator Sep 24th 2019

Rashida Ferrand, PI: September 24th 2019

23 Background

The statistical analysis plan (SAP) for the BREATHE (Broncho-pulmonary function in response to azithromycin treatment for chronic lung disease in HIV-infected children and adolescents) trial is presented here. The plan defines *a priori* the analyses that will be completed for the primary and secondary outcomes of the study, including sub-group analysis. The plan adheres to CONSORT guidelines (1), International Conference on Harmonisation requirements (ICH) and Good Clinical Practice (GCP) guidelines (E6(R1)). The trial is registered with clinicaltrials.gov (NCT02426112) and the protocol has been published (2).

A previous observational study found significant lung function impairment among perinatally HIV-infected children and adolescents in Zimbabwe established on antiretroviral therapy (ART) (3). Lung function impairment was mainly obstructive with no reversibility. Given the use of azithromycin in managing other chronic lung diseases it was hypothesised that prophylactic azithromycin is effective, through its antimicrobial and anti-inflammatory properties, in preventing worsening of lung function and in reducing exacerbations in children and adolescents receiving ART who have HIV associated chronic lung disease. Therefore the aim of the BREATHE trial is to estimate the effect of azithromycin on lung function among individuals aged 6-19 years receiving ART who have HIV-associated chronic lung disease in Zimbabwe and Malawi. Secondary aims are to assess the effect of azithromycin on

general growth and health. This statistical analysis plan (SAP) has been finalised prior to unmasking of treatment allocation.

Trial design

The trial is individually-randomised and placebo-controlled with allocation ratio 1:1, stratified by site (Harare, Zimbabwe, and Blantyre, Malawi). Study participants, care providers, outcome assessors and statisticians remain masked to treatment allocation per protocol. Care providers could be unmasked upon medical need of a participant. Azithromycin or placebo of identical appearance were dispensed by study pharmacists on the basis of participant's weight on the date of dispensing with dose dependant on weight-band. Tablets were to be taken weekly for a course of 48 weeks. Participants attended a study visit two weeks after enrolment into the trial, then at 12, 24, 36, 48, and in a subset, 60, and 72 weeks after enrolment. The trial aimed at recruiting 400 participants and started recruiting in June 2016. Recruitment stopped in September 2018 as planned.

24 Objectives

1: Primary objective: To investigate whether adjuvant treatment with azithromycin results in improvement in lung function in HIV-positive children and adolescents on ART for at least six months with chronic lung disease. The mean FEV1 z-score at 12 months (primary outcome) will be compared between participants who were randomised to receive azithromycin and participants who were randomised to receive placebo.

2: **Secondary objective:** To investigate the intervention effect on exacerbations of lung disease and morbidity.

25 Analysis principles

Primary and secondary outcomes from all randomised participants will be analysed based on an intention to treat (ITT) principle (4) which adjusts for trial site as a binary covariate. That is, participants will be analysed in the group to which they were randomised regardless of whether they received the assigned treatment. Participants who withdraw consent retrospectively for the use of their data will not contribute to analyses.

The primary analysis population will include all eligible participants who were randomised (5), and a sensitivity analysis population will exclude the small number of enrolled participants who were found during data cleaning to not meet the FEV1 inclusion criteria.

Per-protocol analysis will also be presented. The per-protocol population, i.e. participants who adhered to their randomised treatment for a "minimal" number of weeks, has been defined prior to unmasking the study, as those not missing more than 8 doses of the 49 total doses of study medication. For time to event outcomes, the per-protocol population will be defined separately in three month blocks as no more than 2 doses missed per block.

Analysis of harms (adverse event data) will be carried out on the safety (on-treatment) population in both arms. That is, participants will be compared according to the trial arm they were dispensed, for the time they received it regardless of adherence to study medication. Definition of the on-treatment population will be finalised following clinical review of protocol deviations prior to unmasking.

It is expected that due to the randomisation process, there will be few differences in baseline characteristics between study arms. To increase statistical power, baseline values of continuous outcomes will be adjusted for as analysis of covariance and any factors which are imbalanced between trial arms will be adjusted for.

26 Missing data

The amount (number, percentage) of data that is missing will be reported for each of the key outcome variables and covariates. Baseline characteristics of participants with and without missing outcome data will be compared. T-tests or linear regression will be used for normally distributed continuous covariates comparing the mean values of those with and without missing data, Mann Whitney nonparametric test for continuous skewed covariates, and chi squared tests and percentages for categorical covariates.

Possible reasons for missing outcome data and any implications for interpretation of study findings will be discussed. An analysis assuming missing at random (6) may be carried out depending on the patterns and amount of missing data, where covariates associated with missing outcome data will be included in adjusted models. This approach will be decided prior to unmasking after the review of the amount of missing data.

27 Recruitment

The flow of participants through each stage of the study will be illustrated using a diagram as per CONSORT guidelines (Figure 1). Participants undergo three stages to determine eligibility. First patients are assessed as to whether they meet eligibility criteria for age, time on ART, vertical transmission, time on ART, known and stable address, guardian consent, not taking contraindicated medication and without a current exacerbation. If met, patients are then asked to perform a spirometry examination. If able to perform the exam, they are also assessed for reversibility (<12% improvement in FEV1 after spacer-inhaled salbutamol 200 mcg). The final stage to determine eligibility are the clinical assessments including a cardiac examination, and laboratory assessments of sputum and blood. Laboratory results could take up to two weeks to carry out, and only once these are received and eligibility confirmed, written consent is obtained.

28 Characteristics of the study population

Characteristics of participants measured at baseline will be described by trial arm according to the analysis principles already described (Table 1). Statistical tests for imbalance between trial arms will not be carried out because any difference is due to chance by definition if the randomisation has been conducted correctly.

29 Delivery of the intervention (and adherence)

Adherence will be estimated for each participant over their entire study period up until the point they exited the study or consumed the last dose of study medication, whichever came first (Table 2).

Adherence will be defined as the proportion of weeks the participant took the study medication to which they were randomised, to schedule, and at the correct dose for their weight. It will be reported by tablet count of returned tablets, and will be cross-checked against participant's treatment diaries and self-report of missed doses (in cases of discrepancy).

The number of tablets returned will be compared to the number dispensed and the number of tablets expected to be taken (based on weight-based dose and time between visits).

The following assumptions have been made when calculating adherence

- The first dose of study medication was taken on the date first prescribed under supervision of the study nurse with subsequent doses due on the same day of the week.
- Doses will be assumed to have been taken correctly on the scheduled administration day of the week between visits.
- If a participant ran out of medication, they were still considered adherent if medication was received from the pharmacy within three days of the scheduled administration day.
- A ***missed dose*** was defined either as a participant not having any study medication for a given scheduled administration day, or by returning more tablets than were expected to be taken; in the latter case the number of doses missed will be calculated as the number returned divided by the weekly dose.
- ***Incorrect dosing*** was defined as either being prescribed fewer tablets than the protocol's dosing regimen or by returning an amount which divided into the weekly dose with a remainder.
- ***Over-dosing*** was defined as returning fewer tablets than expected. In this case it was assumed that study medication was taken correctly between visits.

Numbers of missed doses, late doses, incorrect doses, over-dosing and periods of unknown adherence (due to missed visit, treatment diary not observed, or withdrawal from the study) will be documented, in addition to the number of weeks where the study medication was taken correctly. Sensitivity analyses will be carried out on periods of unknown adherence following clinical review with varying assumptions made, or that those periods are excluded from both the numerator and denominator in calculations. Optimal adherence will be defined at clinical review prior to unblinding.

30 Sample size considerations

It was estimated that a target sample size of 400 participants, from an estimated 2000 screened individuals, would achieve 80% power for the primary outcome with the following assumptions:

1) A 1:1 allocation ratio between placebo and intervention arms
2) Up to 25% (100) participants un-assessable due to losses to follow-up, deaths or sub-optimal spirometric traces

3) No change to FEV_1 z-scores among participants receiving placebo

In the original protocol estimates of the expected mean and standard deviation (SD) in the placebo arm were obtained from previous work by the study investigators in Zimbabwe to be -2.44 (SD 0.54). The target sample size would allow detection of a 0.17 z-score difference between study arms, an effect size of 0.32.

The sample size justification was subsequently revised and has been reported (2) using estimates of the expected mean and SD from 368 participants screened for the BREATHE trial by January 2017, primarily because the eligibility cut-off for FEV_1 z-score was raised. The expected mean in the placebo arm was revised to be -2.04 (SD 0.82). The target sample size would allow detection of a 0.27 z-score difference between study arms, an effect size of 0.32.

31 Outcome variables

31.1 Primary outcome

The primary outcome is mean Forced Expiratory Volume in one second (FEV_1) z-score (7, 8) after 48 weeks of initiation of the study drug (measurement allowed four weeks either side from 44 weeks up to 52 weeks) compared between trial arms.

31.2 Secondary outcomes

a) Time to first respiratory exacerbation
b) Time to death
c) Incidence of acute exacerbations by 48 weeks
d) Incidence of hospitalisations by 48 weeks
e) Mean (sd) weight-for-age z-score (9) at 48 weeks
f) Incidence of infectious episodes (*Salmonella typhi, malaria* (Malawi only), gastroenteritis) by 48 weeks

31.3 Exploratory outcomes

a) FEV1 at six months, and trajectories of FEV1 z-score, FVC and FEV1/FVC ratio: absolute values, percent predicted and z-scores
b) Weight-for-age z-score at six months
c) Height-for-age-z-score and BMI-for-age-z-score at 48 weeks
d) Underweight (weight for age z-score<-2), stunted (height-for-age-z-score<-2), and low BMI (BMI-for-age-z-score<-2) at 48 weeks
e) Durability of effect (Mean FEV1 z-score at 18 months for those followed up to this time)*

*In the original protocol, this was stipulated as a secondary outcome.

31.4 Reporting of harms

a) Descriptive statistics will be presented for number of adverse events, and severe adverse events defined by DAIDS criteria and a cumulative incidence graph will show how AE have occurred over time

32 Analysis methods

For continuous variables with normally distributed residuals, the mean and standard deviation will be presented. For skewed continuous variables, either geometric mean or the median and inter-quartile range (IQR) will be presented, and for categorical variables the number, total, and percentage in each category. For event data, the number of events, person-time at risk and rate will be presented. Significance tests will be two-sided with 5% level of significance and reported using overall Wald p-values.

For continuous outcomes, mean values and standard deviations for each trial arm will be reported. Linear regression will be used to compare trial arms, to estimate the mean difference and corresponding 95% confidence interval (CI), adjusting for site and baseline value of the measure as a continuous covariate.

For time-to-event outcomes, the median time-to-event and IQR for each trial arm will be reported. Kaplan-Meier plots of cumulative risk will be used for graphical presentation. Cox regression will be used to compare trial arms, to estimate the hazard ratio and corresponding 95% CI, adjusting for site. Follow-up time will stop at the first event unless the participant is censored. Participants will be censored at last clinic visit if lost to follow-up, at date of notification of withdrawal from the study, at reported date of death, or at 49 weeks after receiving their prescription for the study medication (the end of study medication) if an event has not occurred. Any participant with an event (or who is censored) on the first day of follow-up will be included in analysis by setting their time to half a day. The time scale used for time-to-event analyses will be time on study and will commence on the day the participant received their first prescription for study medication.

For multiple-event outcomes, the number of events, person time at risk, rate and 95% CI will be reported for each trial arm. Cox regression with robust standard errors, to allow for the lack of independence among multiple events on the same participant, will be used to compare trial arms, to estimate the incidence rate ratio and corresponding 95% CI, adjusting for site. Follow-up time will stop one week after the last dose was consumed after receiving their prescription for the study medication unless a participant is lost-to-follow-up, withdraws from the study or dies. Censoring will occur at last clinic visit, date of notification of withdrawal, or reported date of death in that case. No person time will be removed during an episode as the participant will still be considered at risk of developing another event. In the case of low numbers of events (<10 events overall, or no events in one trial arm), statistical tests comparing trial arms will not be carried out but summary measures will still be reported.

For binary outcomes, the number with the outcome, total measured and prevalence will be reported by trial arm. Logistic regression will be used to compare trial arms to estimate the odds ratio and corresponding 95% CI, adjusting for site.

Model assumptions will be checked using standard methods.

Pre-specified subgroup analyses will be carried out by (1) trial site (2) baseline severity of lung disease (<-2 vs ≥-2 z-score) and (3) baseline viral load (<1000 copies/ml vs >=1000 copies/ml) (4) baseline weight-for-age z-score<-2 vs ≥-2 and (5) stunted at baseline or not (height for age z-score <-2 vs ≥-2) and baseline age group (< median age vs >= median age). Effect modification will be examined by incorporating an interaction term between subgroups and trial arm.

Analysis of microbiology and immunology outcomes are not described here, but are reported in the trial protocol and separate analysis plans will be developed prior to unmasking.

Results will be reported as in Tables 3 and 4.

33 Review of data prior to unmasking

Prior to unmasking the trial, the *per protocol* and *safety* (*on treatment)* populations will be defined after clinical review of protocol deviations and analysis of adherence data. Start and end dates of adverse events and severity will be reviewed and finalised prior to unmasking. A validation of FEV z-scores will be undertaken on a 5% sample based on traces recorded on the laptops connected to the spirometry machines. Samples will be accepted if all within 0.1 z-scores of database.

1) Review of protocol deviations and adherence data to define "per protocol" analysis i.e. the definition of an adherent participant and optimal dose
2) Review of protocol deviations to define "safety" population ("on treatment population") for analysis of adverse events, that is to be clear about how we are defining treatment arm switches when participants were accidently prescribed drug for the wrong trial arm (a secondary analysis to intention to treat)
3) Review of adverse events start and end dates and severity
4) Validation of FEV z-score calculations on a randomly selected 5% sample, for very low FEV z-scores (<-4), and for z-scores which change by more than 1 SD of either an increase or a decrease

34 Process of unmasking

A final cleaned dataset will be produced after the last follow-up visit has been completed. Distributional assumptions will be tested, exclusions will be defined, missing data patterns will be explored, and the analysis plan will be finalised. A masked analysis will then be conducted by the trial statistician with the allocated arms labelled A and B as provided by the independent statistician. The masked results will be discussed and an interpretation agreed at an investigator's meeting in September 2019. An envelope containing the treatment allocation will be opened after this discussion at the investigator's meeting.

References

1. Schulz KF, Altman DG, Moher D, Group C. CONSORT 2010 statement: updated guidelines for reporting parallel group randomised trials. BMJ. 2010;340:c332.

2. Gonzalez-Martinez C, Kranzer K, McHugh G, Corbett EL, Mujuru H, Nicol MP, et al. Azithromycin versus placebo for the treatment of HIV-associated chronic lung disease in children and adolescents (BREATHE trial): study protocol for a randomised controlled trial. Trials. 2017;18(1):622.
3. Desai SR, Nair A, Rylance J, Mujuru H, Nathoo K, McHugh G, et al. HIV-Associated Chronic Lung Disease in Children and Adolescents in Zimbabwe: Chest Radiographic and High-Resolution Computed Tomography Findings. Clin Infect Dis. 2017.
4. Kahan BC, Morris TP. Analysis of multicentre trials with continuous outcomes: when and how should we account for centre effects? Stat Med. 2013;32(7):1136-49.
5. Fergusson D, Aaron SD, Guyatt G, Hebert P. Post-randomisation exclusions: the intention to treat principle and excluding patients from analysis. BMJ. 2002;325(7365):652-4.
6. Sterne JAC, White IR, Carlin JB, Spratt M, Royston P, Kenward MG, et al. Multiple imputation for missing data in epidemiological and clinical research: potential and pitfalls. Bmj-British Medical Journal. 2009;339.
7. Arigliani M, Canciani MC, Mottini G, Altomare M, Magnolato A, Loa Clemente SV, et al. Evaluation of the Global Lung Initiative 2012 Reference Values for Spirometry in African Children. Am J Respir Crit Care Med. 2017;195(2):229-36.
8. Quanjer PH, Stanojevic S, Cole TJ, Baur X, Hall GL, Culver BH, et al. Multi-ethnic reference values for spirometry for the 3-95-yr age range: the global lung function 2012 equations. Eur Respir J. 2012;40(6):1324-43.
9. Cole TJ, Freeman JV, Preece MA. British 1990 growth reference centiles for weight, height, body mass index and head circumference fitted by maximum penalized likelihood. Stat Med. 1998;17(4):407-29.

Appendix 1: CONSORT 2010 checklist of information to include when reporting a randomised trial*

Section/Topic	Item No	Checklist item	Reported on page No
**Title and abstract**			
	1a	Identification as a randomised trial in the title	________
	1b	Structured summary of trial design, methods, results, and conclusions (for specific guidance see CONSORT for abstracts)	________
**Introduction**			
Background and objectives	2a	Scientific background and explanation of rationale	________
	2b	Specific objectives or hypotheses	________
**Methods**			
Trial design	3a	Description of trial design (such as parallel, factorial) including allocation ratio	________
	3b	Important changes to methods after trial commencement (such as eligibility criteria), with reasons	________
Participants	4a	Eligibility criteria for participants	________
	4b	Settings and locations where the data were collected	________
Interventions	5	The interventions for each group with sufficient details to allow replication, including how and when they were actually administered	________
Outcomes	6a	Completely defined pre-specified primary and secondary outcome measures, including how and when they were assessed	________
	6b	Any changes to trial outcomes after the trial commenced, with reasons	________
Sample size	7a	How sample size was determined	________
	7b	When applicable, explanation of any interim analyses and stopping guidelines	________

Randomisation:			
Sequence generation	8a	Method used to generate the random allocation sequence	
	8b	Type of randomisation; details of any restriction (such as blocking and block size)	
Allocation concealment mechanism	9	Mechanism used to implement the random allocation sequence (such as sequentially numbered containers), describing any steps taken to conceal the sequence until interventions were assigned	
Implementation	10	Who generated the random allocation sequence, who enrolled participants, and who assigned participants to interventions	
Blinding	11a	If done, who was blinded after assignment to interventions (for example, participants, care providers, those assessing outcomes) and how	
	11b	If relevant, description of the similarity of interventions	
Statistical methods	12a	Statistical methods used to compare groups for primary and secondary outcomes	
	12b	Methods for additional analyses, such as subgroup analyses and adjusted analyses	
**Results**			
Participant flow (a diagram is strongly recommended)	13a	For each group, the numbers of participants who were randomly assigned, received intended treatment, and were analysed for the primary outcome	
	13b	For each group, losses and exclusions after randomisation, together with reasons	
Recruitment	14a	Dates defining the periods of recruitment and follow-up	
	14b	Why the trial ended or was stopped	
Baseline data	15	A table showing baseline demographic and clinical characteristics for each group	
Numbers analysed	16	For each group, number of participants (denominator) included in each analysis and whether the analysis was by original assigned groups	

Outcomes and estimation	17a	For each primary and secondary outcome, results for each group, and the estimated effect size and its precision (such as 95% confidence interval)	______
	17b	For binary outcomes, presentation of both absolute and relative effect sizes is recommended	______
Ancillary analyses	18	Results of any other analyses performed, including subgroup analyses and adjusted analyses, distinguishing pre-specified from exploratory	______
Harms	19	All-important harms or unintended effects in each group (for specific guidance see CONSORT for harms)	______
**Discussion**			
Limitations	20	Trial limitations, addressing sources of potential bias, imprecision, and, if relevant, multiplicity of analyses	______
Generalisability	21	Generalisability (external validity, applicability) of the trial findings	______
Interpretation	22	Interpretation consistent with results, balancing benefits and harms, and considering other relevant evidence	______
**Other information**			
Registration	23	Registration number and name of trial registry	______
Protocol	24	Where the full trial protocol can be accessed, if available	______
Funding	25	Sources of funding and other support (such as supply of drugs), role of funders	______

*We strongly recommend reading this statement in conjunction with the CONSORT 2010 Explanation and Elaboration for important clarifications on all the items. If relevant, we also recommend reading CONSORT extensions for cluster randomised trials, non-inferiority and equivalence trials, non-pharmacological treatments, herbal interventions, and pragmatic trials. Additional extensions are forthcoming: for those and for up to date references relevant to this checklist, see www.consort-statement.org.

Figure 1: CONSORT Flow Diagram BREATHE trial

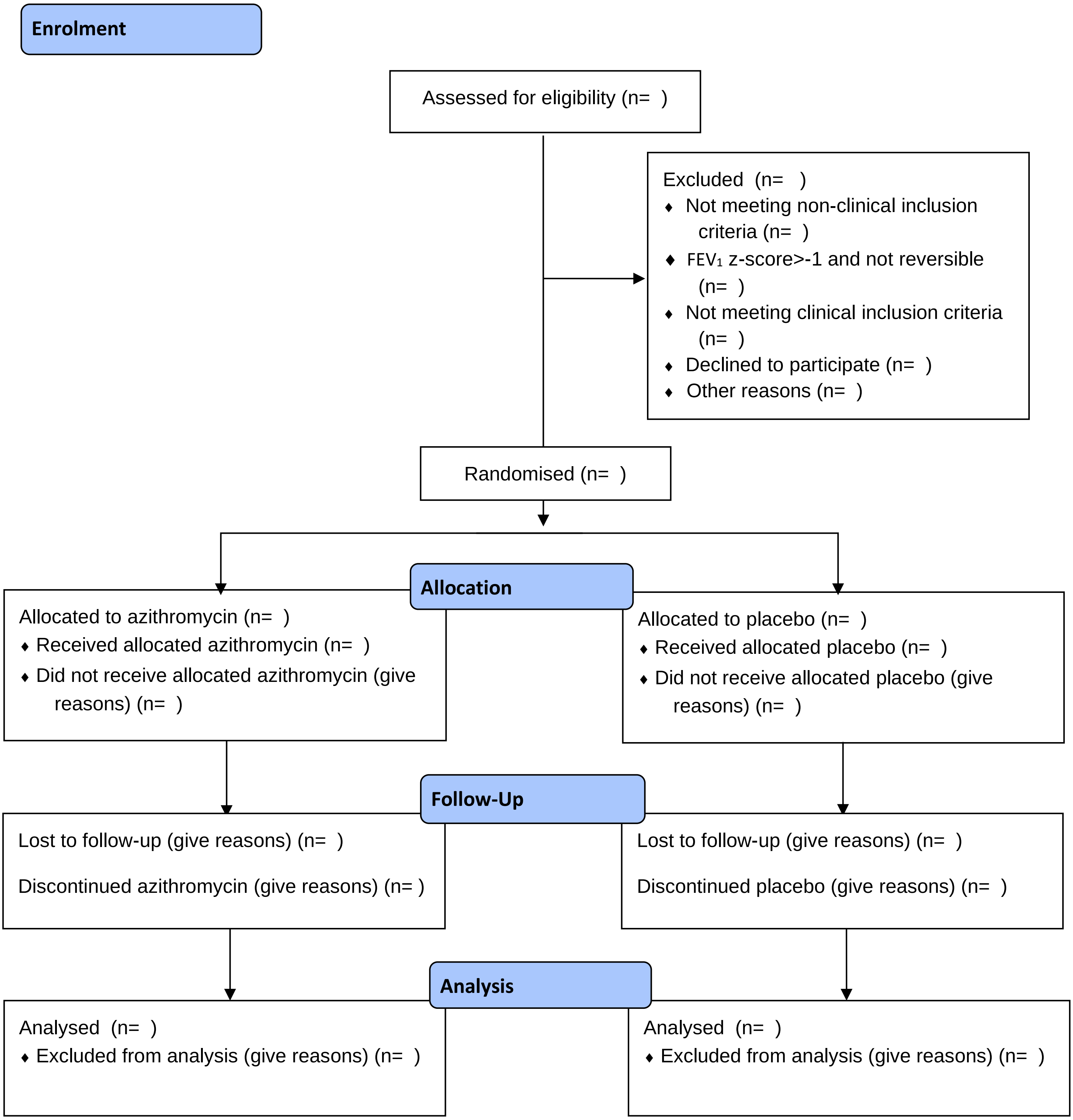

Table 1: Baseline characteristics

Baseline characteristics	Azithromycin arm	Placebo arm
**Demographic**		
Sex, female, n (%)		
Age, median, y		
Attending school currently, n (%)		
Number of days missed in past 1 month, median		
Same age as most children in their class, n (%)		
Ever repeated a grade, n (%)		
**HIV**		
Time since diagnosis, y, median (IQR)		
Age at diagnosis, y, median (IQR)		
Duration on ART, y median (IQR)		
Age at ART start, y, median (IQR)		
HIV VL, median log10 copies/ml (IQR)		
VL<1000 copies/ml, n (%)		
CD4, cells/mm^3 , median (IQR)		
CD4<100 cells/mm3, n (%)		
ART Regimen, n (%)		
EFV/NVP		
ATV/LPV		
Other (Dolutegraivr/Darunavir)		
Taking cotrimoxazole prophylaxis, n (%)		
**Clinical**		
FEV1 z-score, mean (SD)		
FEV_1 (L), mean (SD)		
FEV_1 %, median (IQR)		
FVC (mL), mean (SD)		
FVC %, median (IQR)		
FEV_1/FVC ratio, median (IQR)		
Weight (kg)		
Height (m)		
Weight-for-age z-score, mean (SD)		
Weight- for-age z-score <-2, n (%)		
Height-for-age z-score, mean (SD)		
Height-for-age z-score <-2, n (%)		
BMI-for-age z-score, mean (SD)		
BMI-for-age z-score <-2, n (%)		
Past history of TB, n (%)		
Admitted for chest problems in the past one year, n (%)		
Current cough, n (%)		
Coughing up sputum, n (%)		
Shortness of breath, n (%)		
Resp rate, mean		
Heart rate, mean		
Shuttle walk, duration, mins, secs, mean		

Table 2: Describing adherence

	Azithromycin arm				Placebo arm			
**Adherence measure**	**Completed follow-up**	**Died**	**Patient chose to withdraw**	**Physician stopped study medication**	**Completed follow-up**	**Died**	**Patient chose to withdraw**	**Physician stopped study medication**
**Participants dispensed study medication, n**								
**Person years of follow-up on treatment**								
**Dispensing occasions**								
Total number								
Median (IQR) per participant								
**Dispensing occasions which occurred on the day a dose was due**								
Total number								
% of all occasions								
**Tablet return occasions**								
Total number								
Median (IQR) per participant								
**Tablet return occasions suggesting over-dosing (>110% of tablets)**								
Total number								
% of all occasions								
Median (IQR) per participant								
Median % (IQR) per participant								
**Weeks expected to take medication**								
Total number								
Median (IQR) per participant								
**Weeks adherent total**								

Total number								
% of all weeks								
Median (IQR) per participant								
Median % (IQR) per participant								
**Weeks adherent – on exact dose day**								
Total number								
% of all weeks								
Median (IQR) per participant								
Median % (IQR) per participant								
**Weeks adherent – within 3 days of dose day but not exact day**								
Total number								
% of all weeks								
Median (IQR) per participant								
Median % (IQR) per participant								
**Weeks adherent (suspected over-dosing)**								
Total number								
% of all weeks								
Median (IQR) per participant								
Median % (IQR) per participant								
**Weeks missed because finished tablets**								
Total number								
% of all weeks								
Median (IQR) per participant								
Median % (IQR) per participant								
**Weeks missed as reported by participant**								
Total number								
% of all weeks								
Median (IQR) per participant								
Median % (IQR) per participant								

**Weeks incorrect dosing due to prescription error**								
Total number								
% of all weeks								
Median (IQR) per participant								
Median % (IQR) per participant								
**Weeks incorrect dosing by tablet return**								
Total number								
% of all weeks								
Median (IQR) per participant								
Median % (IQR) per participant								

Table 3: Effect of the intervention on lung function and growth

Outcome	Azithromycin arm	Placebo arm	Adjusted Mean Difference (95% CI)
	Mean (SD)	Mean (SD)	
FEV_1 z-score, mean (SD)			
Weight-for-age z-score, mean (SD)			

Table 4: Effect of the intervention on time to event secondary outcomes

	Azithromycin arm			Placebo arm			
	Events	Person years	Rate/100py (95% CI)	Events	Person years	Rate/100py (95% CI)	Adjusted Rate Ratio (95% CI)
Time to death							
Time to first exacerbation							
All exacerbations							
All hospitalisations							
Malaria episodes							
Salmonella typhi and non-typhi blood stream infections							
All gastroenteritis episodes							

Table 5: Adverse events (safety population)

	**Azithromycin arm**	**Placebo arm**
	**Events**	**Events**
**All adverse events***		
**Related**		
**Unrelated**		
**All severe adverse events**		
**Related**		
**Unrelated**		

* By DAIDS grading

Table 6: Effect of the intervention on exploratory lung function and growth outcomes

Outcome	Azithromycin arm	Placebo arm	Adjusted Mean Difference (95% CI)
	Mean (SD)	Mean (SD)	
FEV_1 (L), mean (SD)			
FEV_1 %, median (IQR)			
FVC (mL), mean (SD)			
FVC %, median (IQR)			
FEV_1/FVC ratio, median (IQR)			
Height-for-age z-score, mean (SD)			
BMI-for-age z-score, mean (SD)			
			Adjusted OR (95% CI)
Not underweight			reference
Underweight			
Not stunted			reference
Stunted			
Not low BMI			reference
Low BMI			
