# Supplementary material for: Effect of Once-Weekly Azithromycin vs Placebo in Children With HIV-Associated Chronic Lung Disease: The BREATHE Randomized Clinical Trial
Source: JAMA Netw Open. 2020 Dec 17;3(12):e2028484. doi: 10.1001/jamanetworkopen.2020.28484 (PMC7747021; doi:10.1001/jamanetworkopen.2020.28484)
Supplement: Supplement 2. — eAppendix. Multiple Imputation Models eTable 1. Baseline Characteristics by Arm for Participants Analyzed for the Primary Outcome eTable 2. Baseline Characteristics Comparing Participants With and Without Primary Outcome eTable 3. Number of Adverse Events eTable 4. Intervention Effect (Adjusted Mean Difference) in Post Hoc Subgroup Analysis by Recruitment Date eReference [file jamanetwopen-e2028484-s002.pdf]

Supplemental Online Content

Ferrand RA, McHugh G, Rehman AM, et al; BREATHE Trial Group. Effect of once-weekly azithromycin vs placebo in children with HIV-associated chronic lung disease: the BREATHE randomized clinical trial. *JAMA Netw Open*. 2020;3(12):e2028484. doi:10.1001/jamanetworkopen.2020.28484

eAppendix. Multiple Imputation Models

eTable 1. Baseline Characteristics by Arm for Participants Analyzed for the Primary Outcome

eTable 2. Baseline Characteristics Comparing Participants With and Without Primary Outcome

eTable 3. Number of Adverse Events

eTable 4. Intervention Effect (Adjusted Mean Difference) in Post Hoc Subgroup Analysis by Recruitment Date

eReference

This supplemental material has been provided by the authors to give readers additional information about their work.

© 2020 Ferrand RA et al. *JAMA Network Open.*

eAppendix: Multiple Imputation Models

In post-hoc sensitivity analysis we used multiple imputation methods to impute missing outcome data for those lost to follow-up (n=5), those who withdrew (n=18), those measured outside of the 44- to 52-week assessment window (n=7) and those who did not have their lung function measured at 48 weeks (n=6), but not for those who died (n=3); we included 344 participants in this post-hoc analysis. We used the "mi ice" command to generate 10 multiple imputation datasets using chained equations (MICE) to allow for the inclusion of categorical auxiliary variables. We took an inclusive approach and included site, sex, enrolment FEV_1 z-score, log of enrolment CD4 cell count, age category, log10 of viral load at enrolment, enrolment anthropometry (weight for age z-score, height-for-age z-score and BMI-for-age z-score), and ART regimen (first or second line) as auxiliary variables based on their association with both missingness (with formal statistical testing $p<0.1$; eTable 2) and lung function (without formal statistical testing). We did not include current cough or abnormal heart rate in imputation models because of small numbers of events producing model instability. Estimation was conducted using "mi estimate" with robust standard errors.

© 2020 Ferrand RA et al. *JAMA Network Open.*

eTable 1: Baseline Characteristics by Arm for Participants Analyzed for the Primary Outcome

Demographic Characteristics	AZM arm N=162	Placebo arm N=146
Median Age (IQR) – year	14·7 (12·6 – 16·5)	15·9 (13·0 – 18·1)
Female sex: no. (%)	73 (45·1)	73 (50·0)
Currently in school: no. (%)[a]	139 (85·8)	117 (80·1)
**HIV Characteristics**		
Median Age at diagnosis (IQR) – year	7·2 (3·5 – 9·9)	8·2 (4·8 – 11·1)
Cotrimoxazole prophylaxis: no. (%)	146 (90.1)	130 (89.0)
Median Duration on ART (IQR) - year	5·9 (3·7 – 8·7)	6·4 (3·9 – 8·3)
Median HIV viral load log 10 copies/ml (IQR)[a]	2·4 (1·6- 4·0)	2·6 (1·6- 4·0)
HIV viral load<1000 copies/ml: no. (%)[a]	96 (59·3)	82 (56·2)
Median CD4 cell count /mm^3 (IQR)	617 (439 – 793)	555·5 (325 – 779)
**Lung function**		
Mean FEV1 z-score (SD)	-2·01 (0·77)	-1·99 (0·77)
Mean FEV1 (SD) – litres	1·59 (0·50)	1·72 (0·53)
Mean FEV1 % (SD)	73·2 (10·5)	73·6 (10·5)
Mean FVC z-score (SD)[a]	-1·78 (0·97)	-1·70 (0·93)
Mean FVC (SD) – litres[a]	1·87 (0·58)	2·05 (0·62)
Mean FVC % (SD)[a]	77·7 (12·0)	78·6 (11·6)
Mean FEV1/FVC ratio z-score (SD)[a]	-0·65 (1·14)	-0·75 (1·18)
Mean FEV1/FVC ratio (SD)[a]	0·85 (0·08)	0·84 (0·09)
**Clinical Characteristics**		
Mean weight-for-age z-score (SD)	-2·29 (1·42)	-2·02 (1·31)
Underweight: no. (%)	95 (58·6)	70 (48·0)
Mean height-for-age z-score (SD)	-2·21 (1·18)	-1·97 (1·05)
Stunted: no. (%)	91 (56·2)	65 (44·5)
History of tuberculosis: no. (%)[a]	54 (33·3)	35 (24·0)
Admitted for chest problems in last year: no. (%)	2 (1·2)	3 (2·1)
Current cough: no. (%)	13 (8·0)	13 (8·9)
Coughing up sputum: no.[b]	7	12
Shortness of breath: no. (%)	5 (3·1)	1 (0·7)
Mean Respiratory rate (SD)	22·3 (3·0)	22·5 (2·7)
Abnormal RR: no. (%)[c]	63 (38·9)	72 (49·3)
Mean Oxygen saturation (SD)	96·6 (3·1)	96·8 (2·4)
Oxygen saturation <92%: no (%)	6 (3·7)	9 (6·2)
Mean Heart rate (SD)	87·3 (12·5)	85·4 (11·1)
Abnormal HR: no (%)[c]	5 (3·1)	5 (3·4)
Mean Shuttle walk duration (SD) – minutes:seconds[a]	10:28 (1:58)	10:52 (2:05)

P-values from two-sample t-test for comparison of means, Wilcoxon rank sum test for comparison of medians, chi-squared test for comparison of proportions or Fisher's exact test for comparison of proportions with any variable count ≤ 5.

[a] *Missing values: currently in school n=1 AZM arm and n=2 placebo arm; HIV viral load n=2 AZM arm; FVC n=4 placebo arm and n=3 AZM arm; History of tuberculosis n=1 AZM arm; Shuttle walk n=5 placebo arm n=5 AZM arm*

[b] *Only for those with a current cough*

[c] *Age-defined cut-offs used based on*[1]

© 2020 Ferrand RA et al. *JAMA Network Open.*

eTable 2: Baseline Characteristics Comparing Participants With and Without Primary Outcome

**Demographic Characteristics**	**Primary outcome unavailable N=39**	**Primary outcome available N=308**	**p-value**
Median Age (IQR) – year	15·7 (13·7 – 18·6)	15·2 (12·7 – 17·5)	0·11
Female sex: no. (%)	24 (61·5)	146 (47·4)	0·10
Currently in school: no· (%)[a]	29 (74·4)	256 (83·1)	0·34
**HIV Characteristics**			
Median Age at diagnosis (IQR) – year	8·7 (6·7 – 10·9)	7·5 (3·9 – 10·4)	0·12
Cotrimoxazole prophylaxis: no. (%)	276 (89.6)	37 (94.9)	0.56
Median Duration on ART (IQR) – year	6·7 (4·0 – 8·9)	6·2 (3·8 – 8·4)	0·85
Median HIV viral load log 10 copies/ml (IQR)[a]	3·5 (1·9- 4·7)	2·6 (1·6- 4·0)	0·04
HIV viral load<1000 copies/ml: no. (%)[a]	23 (59·0)	128 (41·8)	0·04
Median CD4 cell count /mm^3 (IQR)	499 (269 – 677)	590 (382 – 786)	0·07
CD4 cell count < 200/mm^3: no. (%)	5 (12·8)	29 (9·4)	0·33
**Lung function**			
Mean FEV1 z-score (SD)	-2·01 (0·63)	-2·00 (0·77)	0·95
Mean FEV1 (SD) – litres	1·68 (0·53)	1·65 (0·52)	0·78
Mean FEV1 % (SD)	73·5 (8·5)	73·4 (10·5)	0·97
Mean FVC z-score (SD)[a]	-1·71 (0·72)	-1·74 (0·95)	0·86
Mean FVC (SD) – litres[a]	2·00 (0·65)	1·96 (0·61)	0·73
Mean FVC % (SD)[a]	78·2 (9·0)	78·1 (11·8)	0·96
Mean FEV1/FVC ratio z-score (SD)[a]	-0·72 (0·91)	-0·70 (1·16)	0·92
Mean FEV1/FVC ratio (SD)[a]	0·85 (0·06)	0·84 (0·08)	0·66
**Clinical Characteristics**			
Mean weight-for-age z-score (SD)	-2·08 (2·08)	-2·16 (1·37)	0·75
Underweight: no. (%)	16 (41·0)	165 (53·6)	0·14
Mean height-for-age z-score (SD)	-2·18 (1·77)	-2·10 (1·12)	0·68
Stunted: no. (%)	19 (48·7)	156 (50·7)	0·82
History of tuberculosis: no. (%)[a]	8 (20·5)	89 (28·9)	0·51
Admitted for chest problems in last year: no· (%)	1 (2·6)	3 (1·7)	0·51
Current cough: no. (%)[a]	5 (12·8)	26 (8·4)	0·05
Coughing up sputum: no.[b]	5	19	-
Shortness of breath: no. (%)	0 (0·0)	6 (2·0)	-
Mean Respiratory rate (SD)	22·4 (4·8)	22·4 (2·8)	0·95
Abnormal RR: no. (%)[c]	16 (41·0)	135 (43·8)	0·74
Mean Oxygen saturation (SD)	96·7 (2·3)	96·7 (2·8)	0·96
Oxygen saturation <92%: no (%)	2 (5·1)	15 (4·9)	1.00
Mean Heart rate (SD)	87·9 (13·3)	86·4 (11·9)	0·47
Abnormal HR: no (%)[c]	4 (10·3)	10 (3·3)	0.06
Mean Shuttle walk duration (SD) – minutes:seconds[a]	10:20 (1:46)	10:39 (2:02)	0·36

P-values from two-sample t-test for comparison of means, Wilcoxon rank sum test for comparison of medians, chi-squared test for comparison of proportions or Fisher's exact test for comparison of proportions with any variable count ≤ 5.
[a] Missing values: currently in school n=1 without and n=3 with data; HIV viral load n=2 with data; FVC n=1 without and n=7 with data; History of tuberculosis n=1 with data; Shuttle walk n=2 without and n=13 with data
[b] Only for those with a current cough
[c]Age-defined cut-offs used based on[1]

© 2020 Ferrand RA et al. *JAMA Network Open.*

eTable 3: Number of Adverse Events

Event	AZM arm n=173	Placebo arm n=174
**Adverse events**		
Any adverse event	96	93
Any serious adverse event[a]	3	17
Any event leading to discontinuation of trial drug[b]	3	4
**Trial drug related: DAIDS grading**		
1	46	19
2	4	2
3	0	0
4	0	0
5	0	0
**Not trial drug related: DAIDS grading**		
1	36	46
2	8	10
3	2	12
4	0	2
5	0	2

Serious adverse event defined as life threatening, resulting in hospitalization or death

[a] *Placebo arm: Meningo-encephalitis n=3, Anemia n=3, Pulmonary tuberculosis n=2, Esophageal candidiasis n=2, , vomiting n=1, weight loss n=4, hyperthyroidism n=1, Efavirenz induced neuropsychiatric symptoms n=1; AZM arm: Herpes simplex infection n=1, Gastritis n=1, Anemia n=1*

[b] *Placebo arm: Prolonged QTc interval n=2, pregnancy n=2; AZM arm: Prolonged QTc interval n=1, Pregnancy n=1, skin rash n=1*

© 2020 Ferrand RA et al. *JAMA Network Open.*

eTable 4: Intervention Effect (Adjusted Mean Difference) in Post Hoc Subgroup Analysis by Recruitment Date

Subgroup	n	Placebo mean (SD)	n	AZM mean (SD)	Adjusted mean difference (95% CI)	p-value for interaction
Enrolled with inclusion criteria FEV_1 z-score <-1.64	38	-2.35 (1.05)	42	-2.38 (0.83)	-0.01 (-0.34, 0.32)	
Enrolled with inclusion criteria FEV_1 z-score <-1	108	-1.81 (0.82)	120	-1.73 (0.86)	0.08 (-0.08, 0.25)	0.61

Footnote:

Protocol v1 dated 15th Dec 2015 defined chronic lung disease as having a FEV_1 z-score<-1.64. Following on from an investigator's meeting in 2017 it was decided that in order to include those with a FEV_1 z-score at the lower limits of normal to increase the FEV_1 z-score eligibility to -1. Protocol v2.1 dated 9th May 2017 in Zimbabwe and 1st June 2017 in Malawi included participants whose FEV_1 z-score<-1. Participants whose FEV_1 z-score measured between -1.0 and -1.64, prior to those dates were invited for rescreening to determine study eligibility according to the new protocol. Post-hoc analysis was conducted after request from peer reviewer.

© 2020 Ferrand RA et al. *JAMA Network Open.*

eReference

1. O'Leary F, Hayen A, Lockie F, Peat J. Defining normal ranges and centiles for heart and respiratory rates in infants and children: a cross-sectional study of patients attending an Australian tertiary hospital paediatric emergency department. Arch Dis Child 2015;100:733-7.

© 2020 Ferrand RA et al. *JAMA Network Open.*
