# Supplementary material for: Effect of Once-Weekly Azithromycin vs Placebo in Children With HIV-Associated Chronic Lung Disease: The BREATHE Randomized Clinical Trial
Source: JAMA Netw Open. 2020 Dec 17;3(12):e2028484. doi: 10.1001/jamanetworkopen.2020.28484 (PMC7747021; doi:10.1001/jamanetworkopen.2020.28484)
Supplement: Supplement 3. — Data Sharing Statement [file jamanetwopen-e2028484-s003.pdf]

Data Sharing Statement

Ferrand. Effect of Once-Weekly Azithromycin vs Placebo in Children With HIV-Associated Chronic Lung Disease. *JAMA Netw Open*. Published December 17, 2020. doi:10.1001/jamanetworkopen.2020.28484

Data
Data available: Yes
Data types: Deidentified participant data, Data dictionary
How to access data: Individual, anonymized participant data and a data dictionary will be available through the LSHTM repository (Data Compass) 12 months after publication of trial results
When available: beginning date: 06-01-2021

Supporting Documents
Document types: Informed consent form
How to access documents: Request to rashida.ferrand@lshtm.ac.uk
When available: With publication

Additional Information
Who can access the data: Available to anyone
Types of analyses: For any purpose
Mechanisms of data availability: Data available only with approval from the Medical Research Council of Zimbabwe and the Malawi College of Medicine Research Ethics Committee.
